# Severity and Etiology of Incident Stroke in Patients Screened for Atrial Fibrillation vs Usual Care and the Impact of Prior Stroke: A Post Hoc Analysis of the LOOP Randomized Clinical Trial

**DOI:** 10.1001/jamaneurol.2022.3031

**Published:** 2022-08-29

**Authors:** Søren Zöga Diederichsen, Kristian Steen Frederiksen, Lucas Yixi Xing, Ketil Jørgen Haugan, Søren Højberg, Axel Brandes, Claus Graff, Morten Salling Olesen, Derk Krieger, Lars Køber, Jesper Hastrup Svendsen

**Affiliations:** 1 Department of Cardiology, Copenhagen University Hospital–Rigshospitalet, Copenhagen, Denmark; 2 Department of Neurology, Danish Dementia Research Centre, Copenhagen University Hospital–Rigshospitalet, Copenhagen, Denmark; 3 Department of Cardiology, Zealand University Hospital Roskilde, Roskilde, Denmark; 4 Department of Cardiology, Copenhagen University Hospital–Bispebjerg, Copenhagen, Denmark; 5 Department of Cardiology, Odense University Hospital, Odense, Denmark; 6 Department of Clinical Research, Faculty of Health Sciences, University of Southern Denmark, Odense, Denmark; 7 Department of Internal Medicine–Cardiology, University Hospital of Southern Denmark–Esbjerg, Esbjerg, Denmark; 8 Department of Health Science and Technology, Aalborg University, Aalborg, Denmark; 9 Department of Biomedical Sciences, Faculty of Health and Medical Sciences, University of Copenhagen, Copenhagen, Denmark; 10 Stroke Unit, Mediclinic City Hospital, Dubai, United Arab Emirates; 11 Department of Clinical Medicine, Faculty of Health and Medical Sciences, University of Copenhagen, Copenhagen, Denmark

## Abstract

**Importance:**

Atrial fibrillation (AF) screening trials have failed to demonstrate a significant reduction in stroke risk. The impact on stroke severity and the importance of prior strokes are unknown.

**Objective:**

To assess stroke characteristics in patients undergoing implantable loop recorder (ILR) screening for AF vs usual care and assess the importance of prior stroke.

**Design, Setting, and Participants:**

This was a post hoc analysis of the Atrial Fibrillation Detected by Continuous Electrocardiogram Monitoring Using Implantable Loop Recorder to Prevent Stroke in High-Risk Individuals (LOOP) randomized clinical trial. Persons 70 years or older without known AF but diagnosed with 1 or more of the following, hypertension, diabetes, heart failure, or prior stroke, were screened for inclusion. Four sites in Denmark recruited participants by letter between January 31, 2014, and May 17, 2016. The median (IQR) follow-up period was 65 (59-70) months. Data were analyzed from April 1 to May 31, 2022.

**Interventions:**

ILR screening for AF and anticoagulation initiation if AF duration of 6 minutes or longer was detected (ILR group) vs usual care (control group).

**Main Outcomes and Measures:**

Adjudicated stroke, classified according to the modified Rankin Scale (mRS) using a score of 3 or more as a cutoff for severe (disabling or lethal) stroke, and according to the Trial of Org 10172 in Acute Stroke Treatment (TOAST) classification for ischemic strokes.

**Results:**

A total of 6205 individuals were screened for inclusion, and 6004 were randomized and included in the analysis; 4503 participants (75%; mean [SD] age, 74.7 [4.1] years; 2375 male [52.7%]) were assigned to the control group and 1501 participants (25%; mean [SD] age, 74.7 [4.1] years; 792 male [52.8%]) were assigned to the ILR group. A total of 794 of 4503 participants (17.6%) in the control group had a history of prior stroke compared with 262 of 1501 participants (17.5%) in the ILR group. During follow-up, AF was diagnosed in 1027 participants (control group, 550 [12%] vs ILR group, 477 [32%]), and anticoagulation was initiated in 89% of these (910). A total of 315 participants (5.2%) had a stroke (control group, 249 [5.5%] vs ILR group, 66 [4.4%]), and the median (IQR) mRS score was 2 (1-3) with no difference across the groups. A total of 272 participants (4.5%) had ischemic stroke (control group, 217 [4.8%] vs ILR group, 55 [3.7%]), and 123 (2.0%) had severe stroke (control group, 100 [2.2%] vs ILR group, 23 [1.5%]), and the hazard ratios comparing the control and ILR groups were 0.76 (95% CI, 0.57-1.03; *P* = .07) and 0.69 (95% CI, 0.44-1.09; *P* = .11), respectively. For participants without prior stroke, the hazard ratios were 0.68 (95% CI, 0.48-0.97; *P* = .04) and 0.54 (95% CI, 0.30-0.97; *P* = .04), respectively.

**Conclusions and Relevance:**

This post hoc analysis of the LOOP randomized clinical trial found that ILR screening for AF did not result in a significant decrease in ischemic or severe strokes compared with usual care. Exploratory subgroup analyses indicated a possible reduction of these outcomes among participants without prior stroke.

**Trial Registration:**

ClinicalTrials.gov Identifier: NCT02036450

## Key Points

**Question:**

Can implantable loop recorder screening for atrial fibrillation reduce the risk of severe stroke in persons with risk factors or with prior stroke?

**Findings:**

In this post hoc analysis of a randomized clinical trial including 6004 participants at high risk of stroke and 1056 with prior stroke, loop recorder screening did not result in a significant reduction in disabling or lethal stroke compared with usual care in all participants or among participants with prior stroke. The vast majority of strokes were ischemic, but cardioembolism was relatively rare.

**Meaning:**

Implantable loop recorder screening for atrial fibrillation in persons at high risk did not result in a significant reduction in the risk of severe stroke.

## Teaser

This post hoc analysis of the Atrial Fibrillation Detected by Continuous Electrocardiogram Monitoring Using Implantable Loop Recorder to Prevent Stroke in High-Risk Individuals (LOOP) randomized clinical trial investigates if implantable loop recorder screening for atrial fibrillation can reduce the risk of severe stroke in individuals with risk factors or prior stroke history.

## Introduction

Stroke is a leading cause of mortality and disability throughout the world.^1,2^ Atrial fibrillation (AF) is an important and often undiagnosed risk factor for stroke. Extended heart-rhythm monitoring increases AF detection in patients with stroke or transient ischemic attack (TIA),^3,4,5,6^ and various screening regimens have demonstrated a yield in AF diagnoses in the broader population.^7^ Still, no trials had reported clinical outcomes following screening until 2021. The Atrial Fibrillation Detected by Continuous Electrocardiogram Monitoring Using Implantable Loop Recorder to Prevent Stroke in High-Risk Individuals (LOOP) study randomly assigned persons 70 years or older and with at least 1 additional stroke risk factor to receive implantable loop recorder (ILR) or usual care,^8^ whereas the Systematic Electrocardiogram Screening for Atrial Fibrillation Among 75-Year-Old Subjects in the Region of Stockholm and Halland, Sweden (STROKESTOP) study randomly assigned Swedish citizens aged 75 years to receive invitation to intermittent rhythm strips or no invitation.^9^ Although these trials failed to demonstrate a significant reduction in stroke risk, they trended toward a clinically relevant benefit, and per-protocol analyses supported this signal.

Observational studies have found that cardioembolic or AF-related strokes have worse prognosis than strokes not related to AF.^10,11^ Therefore, one may speculate that unscreened persons may be worse off when having a stroke than persons screened for AF and treated accordingly. Importantly, increased detection of AF and prescription of anticoagulants could also increase bleeding events and, therefore, a differentiation between stroke characteristics may elucidate any net benefit from AF screening.

The aforementioned LOOP study comprised a well-characterized cohort with a large number of adjudicated strokes. We used this trial to assess the severity and etiology of incident stroke and the importance of prior stroke in persons screened for AF vs usual care.

## Methods

### Study Design

The current study was a post hoc analysis of the LOOP randomized clinical trial conducted at 4 sites in Denmark.^8^ The study design has been published previously (Supplement 1).^12^ Briefly, a sample of individuals 70 years or older with no history of AF but diagnosed with hypertension, diabetes, heart failure, or previous stroke, was randomly identified through national public health care registries and invited to participate. At an initial screening visit, medical history, body weight and height, and blood pressure were obtained along with a standard electrocardiogram to rule out prevalent AF. Eligible participants were randomly assigned in a 1:3 ratio to receive a Reveal LINQ (Medtronic) with remote monitoring and daily review of any arrhythmias (ILR group) vs usual care (control group). AF diagnoses were adjudicated by cardiologists (S.Z.D., K.J.H., S.H., A.B., J.H.S.). In the ILR group, oral anticoagulation was recommended upon detection of AF episode duration of 6 minutes or longer.

Data collection was performed by research nurses supervised by the physicians at each study site. In the ILR group, outcomes were collected during annual on-site study visits with a search of the medical records from all hospital admissions, outpatient visits, and drug prescriptions for the first 3 years, followed by annual phone contact with further review of these records. In the control group, outcomes were collected using a similar strategy but with annual phone contact apart from a single on-site study visit at year 3. At the end of the trial, all participants who were still alive underwent a final assessment within a period of 3 months. Data on race or ethnicity were not systematically gathered because the eligible population was considered rather homogeneous with a vast majority of White individuals. All participants provided written informed consent before enrollment, and the trial was approved by the local Ethics Committee and Data Protection Agency. This LOOP randomized clinical trial followed the Consolidated Standards of Reporting Trials (CONSORT) reporting guidelines.

### Outcomes

All strokes were adjudicated by an event committee consisting of consultant cardiologists and neurologists and further classified by a consultant neurologist (K.S.F.) based on medical records and imaging results. Stroke admissions were classified according to days in hospital grouped as 1 week or less, 1 to 2 weeks, 2 to 3 weeks, and greater than 3 weeks, and discharge outcome grouped as discharge to the patient’s own home, rehabilitation facility, nursing home, or death within 30 days. Stroke severity was classified according to the National Institutes of Health Stroke Scale (NIHSS) at admission using a score of 5 or less, 5 to 16, and 17 or greater as cutoffs for mild, moderate, and severe stroke, respectively,^13,14^ and according to the modified Rankin Scale (mRS) score at 30 days after discharge using a score of 3 or greater as a cutoff for severe (disabling or lethal) stroke opposed to nondisabling or mildly disabling stroke.^15^ Stroke etiology was classified as ischemic vs nonischemic and according to the Trial of Org 10172 in Acute Stroke Treatment (TOAST) classification for ischemic strokes.^16^ In-hospital treatment with thrombolysis, thrombectomy, or carotid intervention was recorded. In participants with more than 1 stroke during follow-up, the event with the highest mRS score was analyzed.

### Statistical Analysis

For summary statistics, continuous variables were presented as mean (SD) for normally distributed variables (and groupwise compared by *t* tests) and median (IQR) for nonnormally distributed variables, whereas categorical variables were presented as frequency (percentage). Event rates were presented as events per 100 person-years (95% CI) and hazard ratios (HRs).

The distributions of hospital stay duration category, discharge outcome, NIHSS category, mRS score, and TOAST classification were visualized according to the raw frequencies for strokes in each study group. Hospital stay duration in days, NIHSS score, and mRS score were groupwise compared using Wilcoxon rank sum test, whereas discharge outcome and TOAST classification were compared using χ^2^ tests. In a supplementary analysis, the mRS was also compared according to diagnosis of AF before or on the day of the event vs no AF within each.

Time-to-event analyses were performed using right censoring at the end of follow-up or death. Cumulative incidences were calculated and groupwise compared using the Aalen-Johansen estimator to account for competing risk of death. Exploratory subgroup analyses were performed according to baseline history of previous stroke, and of prior stroke, TIA, or systemic arterial embolism (SAE), with reports of tests for interaction between randomization group and these baseline conditions. Schoenfeld residuals were evaluated to test the proportional hazards assumption and any violations were reported. These data were analyzed from April 1 to May 31, 2022, using R software, version 4.1.3 (R Foundation) and RStudio, version 1.4.1106. Two-sided *P* values > .05 were considered statistically significant.

## Results

### Study Overview

Between January 31, 2014, and May 17, 2016, 6205 individuals were screened for inclusion and 6004 were randomly assigned: 4503 (75%; mean [SD] age, 74.7 [4.1] years; 2375 male [52.7%]; 2128 female [47.3%]) to the control group, and 1501 (25%; mean [SD] age, 74.7 [4.1] years; 792 male [52.8%]; 709 female [47.2%]) to the ILR group (eFigure 1 in Supplement 2). In addition, in the control and ILR groups, 4066 participants (90.3%) and 1378 participants (91.8%) had a history of hypertension, respectively. At baseline, 794 participants (17.6%) in the control group had a history of prior stroke compared with 262 participants (17.5%) in the ILR group. A total of 1139 participants (25.3%) in the control group and 370 participants (24.7%) in the ILR group had a history of either prior stroke, TIA, or SAE (Table).

**Table.** Baseline Characteristics

Characteristic	No. (%)	
	Control (4503)	ILR (1501)
Sex		
Male	2375 (52.7)	792 (52.8)
Female	2128 (47.3)	709 (47.2)
Age, mean (SD), y	74.7 (4.1)	74.7 (4.1)
Hypertension	4066 (90.3)	1378 (91.8)
Prior stroke	794 (17.6)	262 (17.5)
Prior stroke, TIA, or SAE	1139 (25.3)	370 (24.7)
Diabetes	1288 (28.6)	422 (28.1)
Heart failure	199 (4.4)	67 (4.5)
Prior AMI, CABG, or PCI	614 (13.6)	177 (11.8)
Valvular heart disease	181 (4.0)	63 (4.2)
CHA2DS2-VASc score		
Score, median (IQR)	4 (3-4)	4 (3-4)
2	588 (13.1)	202 (13.5)
3	1494 (33.2)	513 (34.2)
4	1325 (29.4)	419 (27.9)
5	687 (15.3)	244 (16.3)
≥6	409 (9.8)	123 (8.2)
Medical treatment		
Antiplatelets	2204 (48.9)	702 (46.8)
β-Blockers	1172 (26.0)	354 (23.6)
Calcium blockers	1684 (37.4)	562 (37.4)
RA inhibitors	2999 (66.6)	991 (66.0)
Statins	2621 (58.2)	879 (58.6)
Diuretics	1511 (33.6)	495 (33.0)
Physical evaluation		
BMI, mean (SD)^a^	27.6 (4.5)	27.8 (4.7)
Blood pressure, mean (SD), mm Hg		
Systolic	149.8 (19.5)	150.6 (19.2)
Diastolic	83.9 (11.3)	84.7 (11.1)

Abbreviations: AMI, acute myocardial infarction; BMI, body mass index; CABG, coronary artery bypass graft; ILR, implantable loop recorder; PCI, percutaneous coronary intervention; RA, renin-angiotensin; SAE, systemic arterial embolism; TIA, transient ischemic attack.

^a^ Calculated as weight in kilograms divided by height in meters squared.

All patients randomized were included in the analysis, and none were lost to follow-up. The median (IQR) follow-up period was 65 (59-70) months. A total of 1027 participants were diagnosed with AF during follow-up (control group, 550 [12%] vs ILR group, 477 [32%]), of which 910 (89%) initiated anticoagulation (control group, 476 [87%] vs ILR group, 434 [91%]), and 42 (4.6%) later discontinued the treatment.^8^ A total of 315 participants (5.2%) had a stroke during follow-up (control group, 249 [5.5%] vs ILR group, 66 [4.4%]).^8^ Of all 315 patients with incident stroke, 33 (10%) had recurrent stroke during follow-up (control group, 28 [11%] vs ILR group, 5 [7.6%]) and were analyzed according to the event with the highest mRS score. The overall stroke rate in the current study was 1.02 (95% CI, 0.92-1.15) per 100 person-years (control group, 1.08; 95% CI, 0.95-1.23 vs ILR group, 0.86; 95% CI, 0.67-1.10; HR, 0.80; 95% CI, 0.61-1.05; *P* = .10). The rate of recurrent stroke after the index event was 5.11 (95% CI, 3.52-7.18) per 100 person-years, with no difference between the groups (HR, 0.58; 95% CI, 0.22-1.51; *P* = .27) (eFigure 2 in Supplement 2).

### Stroke Severity

Of all 315 stroke admissions, the median (IQR) duration of hospital stay was 6 (4-15) days with no difference in hospital stay across randomization groups (eFigure 3 in Supplement 2). A total of 41 patients (13%) died in-hospital or within 30 days after discharge, whereas 14 (4.4%), 57 (18%), and 203 (64%) were discharged to nursing home, rehabilitation facility, and the patient’s own home, respectively. A higher proportion of patients with stroke in the ILR group were discharged to home compared with the control group, but the difference was not statistically significant (eFigure 3 in Supplement 2).

**Figure 1.**
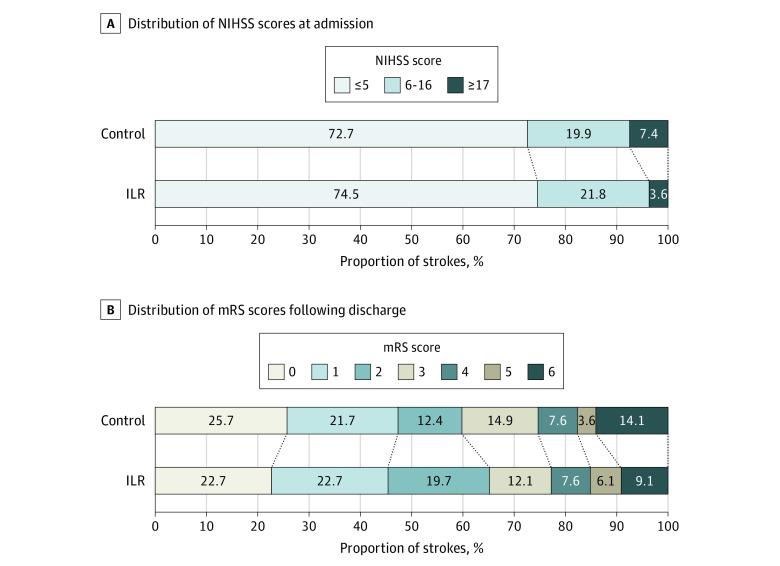
Stroke Severity Grouped by Randomization Group The distribution of National Institutes of Health Stroke Scale (NIHSS) scores at admission (A) and modified Rankin Scale (mRS) scores after discharge from stroke admission (B) are presented in the control and implantable loop recorder (ILR) groups. Wilcoxon rank sum test revealed no difference in NIHSS or mRS, and χ^2^ tests revealed no difference in disabling (mRS ≥3) vs nondisabling strokes or lethal strokes (mRS = 6) vs nonlethal strokes.

A total of 271 patients with stroke (86%) had an available NIHSS score at admission; of these, 198 (73%) had mild, 55 (20%) moderate, and 18 (6.6%) severe stroke, and the median (IQR) score was 3 (1-6) with no significant difference across the randomization groups. All 315 strokes were scored according to the mRS after discharge, and the median (IQR) mRS score was 2 (1-3) with no difference in distribution of mRS score across the groups (Figure 1).

**Figure 2.**
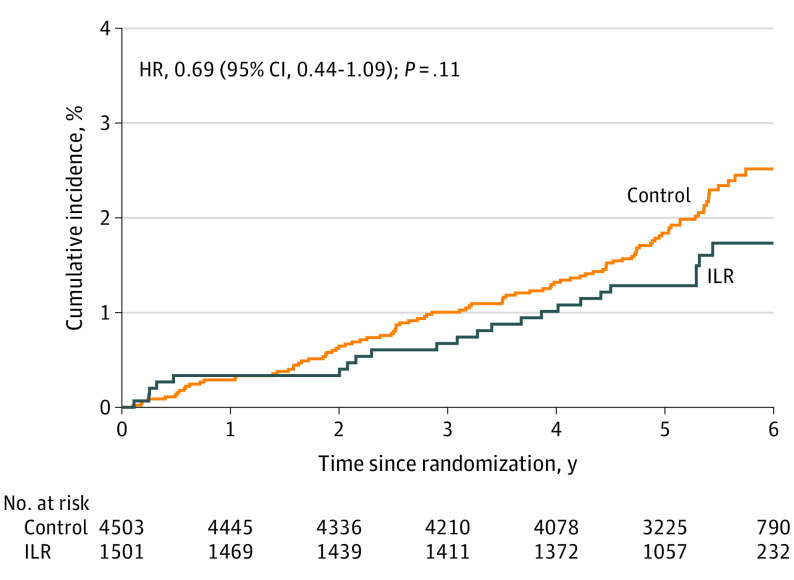
Cumulative Incidence of Severe Stroke The cumulative incidence curves for disabling or lethal stroke (modified Rankin Scale [mRS] score ≥3) in the control and implantable loop recorder (ILR) group are compared. HR indicates hazard ratio.

A total of 123 participants (2.0%) had severe stroke according to the mRS (control group, 100 [2.2%] vs ILR group, 23 [1.5%]), corresponding to 40% and 35% of all strokes in each group, respectively, with an overall event rate of 0.40 (95% CI, 0.33-0.47) per 100 person-years (control group, 0.43; 95% CI, 0.35-0.52 vs ILR group, 0.30; 95% CI, 0.19-0.44; HR, 0.69; 95% CI, 0.44-1.09; *P* = .11) (Figure 2).

A total of 192 participants (3.2%) had mild stroke (control group, 149 [3.3%] vs ILR group, 43 [2.9%]), with an overall event rate of 0.62 (95% CI, 0.54-0.72) per 100 person-years (control group, 0.64; 95% CI, 0.54-0.76 vs ILR group, 0.56; 95% CI, 0.41-0.76; HR, 0.87; 95% CI, 0.62-1.22; *P* = .43) (eFigure 4 in Supplement 2).

### Stroke Etiology

A total of 272 participants (4.5%) had ischemic stroke (control group, 217 [4.8%] vs ILR group, 55 [3.7%]), corresponding to 87% and 83% of all stroke patients in each group, respectively, with an overall rate of 0.87 (95% CI, 0.78-1.00) per 100 person-years (control group, 0.94; 95% CI, 0.82-1.08 vs ILR group, 0.72; 95% CI, 0.54-0.93; HR, 0.76; 95% CI, 0.57-1.03; *P* = .07) (eFigure 5 in Supplement 2).

**Figure 3.**
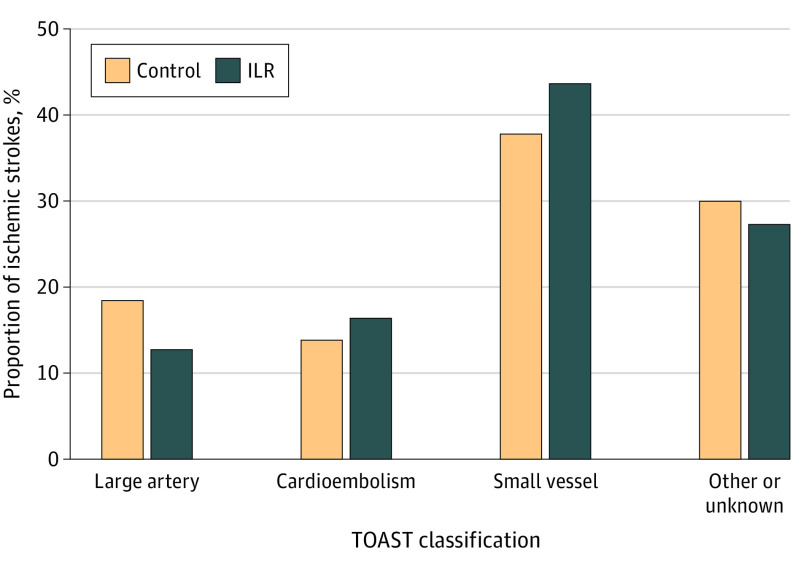
Etiology of Ischemic Strokes The number and proportion of ischemic strokes according to the Trial of Org 10172 in Acute Stroke Treatment (TOAST) classification in the control and implantable loop recorder (ILR) groups are displayed. Other or unknown includes embolic strokes of undetermined source, which was 63 (29%) for the control group and 15 (27%) for the ILR group. χ^2^ Test revealed no difference.

Of all patients with ischemic stroke, 50 (18%) were treated with thrombolysis (control group, 40 [18%] vs ILR group, 10 [18%]), 11 (4.0%) were treated with thrombectomy (control group, 9 [4.2%] vs ILR group, 2 [3.6%]), and 7 were treated with carotid intervention (control group, 5 [2.3%] vs ILR group, 2 [3.6%]), with no significant differences between the groups. The most common TOAST classification was small-vessel disease at 40%, whereas 14% of ischemic strokes were cardioembolic and 25% of undetermined source, and there were no significant differences between the groups (Figure 3).

A total of 117 participants (2.0%) had cardioembolic or embolic stroke of undetermined source (ESUS) (control group, 93 [2.1%] vs ILR group, 24 [1.6%]), with an overall rate of 0.38 (95% CI, 0.31-0.45) per 100 person-years (control group, 0.40; 95% CI, 0.32-0.49 vs ILR group, 0.31; 95% CI, 0.20-0.46; HR, 0.78; 95% CI, 0.50-1.22; *P* = .27) (eFigure 6 in Supplement 2), and 83 participants (1.4%) had severe cardioembolic or ESUS (control group, 67 [1.5%] vs ILR group, 16 [1.1%]), with an overall rate of 0.27 (95% CI, 0.22-0.34) per 100 person-years (control group, 0.29; 95% CI, 0.23-0.37 vs ILR group, 0.21; 95% CI, 0.12-0.33; HR, 0.70; 95% CI, 0.41-1.20; *P* = .20) (eFigure 7 in Supplement 2). A total of 43 patients (0.7%) had hemorrhagic stroke, 45 if analyzed as first event opposed to the event with highest mRS score at recurrent stroke, with no difference between the groups.

### Subgroup Analyses According to Prior Stroke

**Figure 4.**
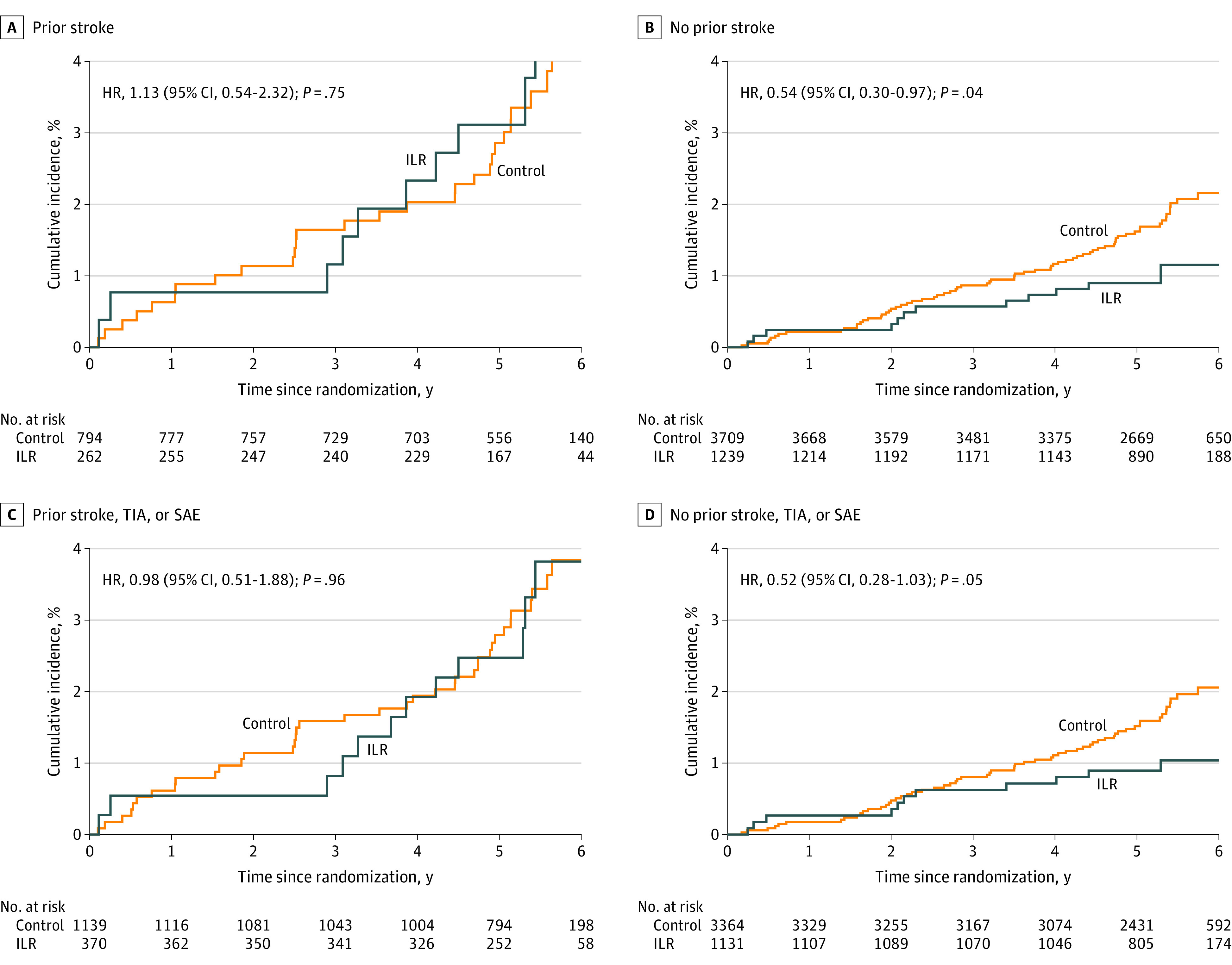
Cumulative Incidence of Severe Stroke Stratified by Stroke History The cumulative incidence curves for disabling or lethal stroke (modified Rankin Scale [mRS] score ≥3) comparing the control and implantable loop recorder (ILR) groups are stratified by prior stroke (A), no prior stroke (B), prior stroke, transient ischemic attack (TIA), or systemic arterial embolism (SAE) (C), and no prior stroke, TIA, or SAE (D). HR indicates hazard ratio.

For the outcome of severe stroke, comparison of the randomization groups yielded an HR of 1.13 (95% CI, 0.54-2.32) among participants with prior stroke (n = 1056) vs 0.54 (95% CI, 0.30-0.97) among participants without prior stroke (n = 4948; *P* value for interaction = .12) and an HR of 0.98 (95% CI, 0.51-1.88) among participants with prior stroke, TIA, or SAE (n = 1509) vs 0.52 (95% CI, 0.28-1.03) among participants without prior stroke, TIA, or SAE (n = 4495; *P* value for interaction = .18) (Figure 4). Corresponding estimates for the outcome of mild stroke were an HR of 0.95 (95% CI, 0.48-1.87) with prior stroke vs 0.85 (95% CI, 0.57-1.26) without prior stroke (*P* value for interaction = .78) and an HR of 0.97 (95% CI, 0.53-1.77) with prior stroke, TIA, or SAE vs 0.83 (95% CI, 0.55-1.26) without prior stroke, TIA, or SAE (*P* value for interaction = .68) (eFigure 8 in Supplement 2). For the outcome of ischemic stroke, the estimates were an HR of 1.03 (95% CI, 0.61-1.76) with prior stroke vs 0.68 (95% CI, 0.48-0.97) without prior stroke (*P* value for interaction = .21) and an HR of 0.97 (95% CI, 0.60-1.57) with prior stroke, TIA, or SAE vs 0.67 (95% CI, 0.46-0.98) without prior stroke, TIA, or SAE (*P* value for interaction = .24) (eFigure 9 in Supplement 2). For the outcome of cardioembolic stroke or ESUS, the estimates were an HR of 1.13 (95% CI, 0.55-2.32) with prior stroke vs 0.64 (95% CI, 0.36-1.15) without prior stroke (*P* value for interaction = .24) and an HR of 1.05 (95% CI, 0.53-2.07) with prior stroke, TIA, or SAE vs 0.65 (95% CI, 0.35-1.18) without prior stroke, TIA, or SAE (*P* value for interaction = .45) (eFigure 10 in Supplement 2). For the outcome of severe cardioembolic stroke or ESUS, the estimates were an HR of 1.49 (95% CI, 0.64-3.45) with prior stroke vs 0.46 (95% CI, 0.22-0.97) without prior stroke (*P* value for interaction = .04) and an HR of 1.43 (95% CI, 0.68-3.02) with prior stroke, TIA, or SAE vs 0.38 (95% CI, 0.16-0.89) without prior stroke, TIA, or SAE (*P* value for interaction = .02) (eFigure 11 in Supplement 2).

### Atrial Fibrillation and Stroke

Of all 315 participants with stroke admissions, 49 (16%) had been diagnosed with AF before their event (control group, 32 [13%] vs ILR group, 17 [26%]), and a further 5 patients were diagnosed with AF on the same day as the event (control group, 4 vs ILR group, 1). The median (IQR) mRS score was 3 (1-6) vs 2 (0-3) for patients with and without AF diagnosis, respectively (*P* < .001). Within the control group, the median (IQR) mRS score was 4 (2-6) vs 1 (0-3) for patients with and without AF diagnosis, respectively (*P* < .001), whereas there was no statistical difference within the ILR group (eFigure 12 in Supplement 2). Of the 49 patients with AF diagnosis before the stroke, 44 (90%) had already initiated anticoagulation (control group, 28 [88%] vs ILR group, 16 [94%]), and these had a median (IQR) mRS score of 2 (1-5) compared with 5 (2-6) in those with untreated AF (*P* = .50).

## Discussion

To our knowledge, this was the first study to assess the severity and etiology of stroke after AF screening vs usual care in persons without AF but with high risk of stroke. The data are from a well-characterized randomized trial with high AF detection in the screening group, high adherence to anticoagulation overall, and a large number of strokes with no loss to follow-up. The main findings were as follows: first, more than 1 in 3 strokes were classified as disabling or lethal (mRS score ≥3) with a nonsignificant 31% reduction in this outcome by ILR screening compared with usual care. Second, the vast majority of strokes were ischemic, and the most common etiology was small-vessel disease, although patients with AF detected had worse strokes than those without. Third, exploratory subgroup analyses indicated an effect on severe strokes from screening in persons without prior stroke, whereas there was no signal toward benefit from screening in patients with prior stroke.

The overall stroke rate of 1% per year, and the etiology, with almost 90% being ischemic, were comparable with population estimates.^1,2,17,18^ The somewhat higher proportion of small-vessel occlusions and lower proportion of cardioembolism in the current study compared with prior cohorts could in part be explained by the eligibility criteria yielding an older population with a very high prevalence of hypertension and no known atrial fibrillation at baseline. The stroke severity was also somewhat higher than in registry studies, with only 47% having no or only slight disability, and the stroke-related mortality of 13% was somewhat higher than recently reported.^11^ This may reflect that our study only included participants with risk factors and that all strokes were adjudicated.

Our study supports previous observations of worse stroke prognosis in patients with a diagnosis of AF than those without an AF diagnosis.^11,19^ This signal was upheld by patients with usual care-detected AF probably owing to patients with ILR being diagnosed earlier, and the possibility that aggressive treatment of subclinical AF decreased the rate of more serious complications is worth mentioning. Overall, fewer participants in the ILR group experienced a disabling or lethal stroke, and the lack of statistical significance could be attributable to insufficient statistical power for this outcome. In support of this notion is the gradual separation of the cumulative incidence curves over time and the much smaller signal for mild strokes. One could question whether adequately powered screening trials are feasible. Albeit being a highly prevalent disease, stroke is a rare event in the individual. This problem is only bigger in the screening-intervention setting compared with single-step intervention trials. With 315 adjudicated strokes, the current trial was comparable in size with the seminal trials on direct oral anticoagulation in AF^20,21,22^ and ESUS,^23,24^ but because of the expectedly low event rate, it was only powered to detect at least 35% relative reduction in stroke risk,^12^ with even less power to detect rarer events such as disabling stroke. Despite the limited evidence,^25^ screening for AF seems to be increasingly applied, whereas the methodologies to detect the arrhythmia expand outside the traditional clinical settings.^7,26^ To summarize the current trial, the bulk of the data supports that intensive screening and treatment of subclinical AF leads to decreased risk of stroke, especially severe stroke, but this comes at a cost of increased risk of bleeding, albeit no signal for hemorrhagic stroke. Future meta-analyses will seek to clarify which persons will benefit from what type of screening.^27^

How intensively to monitor for unknown AF remains an unresolved problem for neurologists who manage stroke.^1^ Albeit not designed to investigate poststroke regimens, the current study does not support ILR screening for AF among patients with a history of stroke. Countering the support for AF screening are arguments that increased focus on AF should not cause decreased focus on other stroke risk factors.^1^ The exploratory subgroup analyses in the current study indicated a possible effect from screening among participants without, not with, prior stroke even though these had lower event rates (Figure 4; eFigures 9-11 in Supplement 2). This may well translate into outpatients at highest risk having too many competing risk factors for detection of subclinical AF to make a difference. Also, only 1 in 4 stroke patients with continuous monitoring had any AF before the event, and only 12% of strokes were deemed cardioembolic by imaging. In all, this highlights that other risk factors than the arrhythmia itself are worth pursuing in terms of stroke prevention. For instance, high systolic blood pressure is an important and treatable risk factor,^28,29^ obesity and metabolic syndrome are risk factors for AF and stroke alike,^30,31,32^ and a large-scale trial is under way to assess the benefit of anticoagulation in patients with stroke who have concurrent atrial cardiomyopathy and sinus rhythm.^33^

### Limitations

This study had several limitations. First, it was a post hoc analysis, and the results should be interpreted with caution, especially the subgroup analyses which were exploratory by nature. The timing of recruitment did not depend on stroke history, and the etiology of strokes before enrollment was unknown. Second, the current trial may be underpowered to assess a smaller but clinically meaningful reduction in risk of disabling stroke or in the distribution of stroke severity across the randomization groups. Third, the more frequent study visits in the ILR group may lead to ascertainment bias with regard to minor events that did not result in hospital contact. Finally, NIHSS could not be assessed for 14% of stroke admissions, and stroke severity scores at admission or after discharge are not perfect measures of long-term prognosis.^34,35^

## Conclusions

In this post hoc randomized clinical trial of ILR screening for AF vs usual care in individuals 70 years or older with risk factors, screening did not result in a significant reduction in disabling or lethal stroke. Exploratory subgroup analyses indicated a possible effect of screening in persons without prior stroke, whereas there was no signal toward benefit in patients with prior stroke. The vast majority of strokes were ischemic, and the most common etiology was small-vessel disease. Cardioembolism was relatively rare, although stroke in patients with AF detected was worse than in patients without.

## Notes

Supplement 1. Trial Protocol

Supplement 2. **eFigure 1.** Trial Flow Chart **eFigure 2.** Cumulative Incidence of Recurrent Stroke on Patients With Incident Stroke **eFigure 3.** Stroke Hospital Stay Duration and Discharge Outcome Grouped by Randomization Group **eFigure 4.** Cumulative Incidence of Mild Stroke **eFigure 5.** Cumulative Incidence of Ischemic Stroke **eFigure 6.** Cumulative Incidence of Cardioembolic Stroke or ESUS **eFigure 7.** Cumulative Incidence of Severe Cardioembolic Stroke or ESUS **eFigure 8.** Cumulative Incidence of Mild Stroke by Stroke History **eFigure 9.** Cumulative Incidence of Ischemic Stroke by Stroke History **eFigure 10.** Cumulative Incidence of Cardioembolic Stroke or ESUS by Stroke History **eFigure 11.** Cumulative Incidence of Severe Cardioembolic Stroke or ESUS by Stroke History **eFigure 12.** Stroke Severity Grouped by AF Diagnosis and Randomization Group

Supplement 3. Data Sharing Statement
