# Supplementary material for: Severity and Etiology of Incident Stroke in Patients Screened for Atrial Fibrillation vs Usual Care and the Impact of Prior Stroke: A Post Hoc Analysis of the LOOP Randomized Clinical Trial
Source: JAMA Neurol. 2022 Aug 29;79(10):997–1004. doi: 10.1001/jamaneurol.2022.3031 (PMC9425290; doi:10.1001/jamaneurol.2022.3031)
Supplement: Supplement 1. — Trial Protocol [file jamaneurol-e223031-s001.pdf]

Implantable Loop Recorder to Detect Atrial Fibrillation and Prevent Stroke (The LOOP study): A Randomized Clinical Trial

Jesper H Svendsen, MD, DMSc, Søren Z Diederichsen MD, PhD, Søren Højbjerg, MD, PhD, Derk Krieger, MD, Claus Graff, MSc, PhD, Christian Kronborg, MSc, PhD, Morten S. Olesen, MSc, PhD, Jonas B Nielsen, MD, PhD, Anders G Holst, MD, PhD,Axel Brandes, MD, DMSc, Ketil J Haugan, MD, PhD, Lars Køber, MD, DMS

This supplement contains the following items:

1. Original protocol, final protocol, summary of changes
2. Original statistical analysis plan (SAP), final SAP, summary of changes

Table of contents

**Original protocol**	**page 3 to 66**
**Final protocol**	**page 67 to 140**
**Summary of changes**	**page 141 to 143**
**Original SAP**	**page 144 to 146**
**Final SAP**	**page 147 to 158**
**Summary of changes**	**page 159 to 160**

Original protocol

Atrial Fibrillation detected by continuous ECG monitoring using implantable loop recorder to prevent stroke in high-risk individuals - The LOOP study

By the Steering Committee of the LOOP Study

Trial Sponsor:

The LOOP Study Steering Committee chairman Jesper Hastrup Svendsen
Department of Cardiology, section 2013
Rigshospitalet
Blegdamsvej 9, DK-2100 Copenhagen, Denmark

Protocol Version 61
Version date 03-12-2013
Protocol number in the Ethics Committe of the Capital Region of Denmark: H-4-2013-025
ClinicalTrials Gov number: xxx

Protocol synopsis

Title:
The LOOP study: Atrial Fibrillation detected by continuous ECG monitoring using implantable Loop recorder to prevent stroke in high-risk individuals.
Background:
Stroke is a major health problem, which affects approximately 16,000 people annually in Denmark, causing severe and burdensome consequences for its victims, who suffer a significant loss in quality of life (QOL), and their families. Furthermore, the healthcare costs due to stroke are sizeable, amounting to more than EUR 38 bn in the EU in 2006. Stroke is often caused by atrial fibrillation (AF), which is the most common type of cardiac arrhythmia. Effective management of AF with anticoagulation therapy is available and may have considerable benefits for patients as well as society. Difficult to detect, however, asymptomatic AF is usually only diagnosed by chance. Recently developed technology (implantable loop recorder; ILR) allows continuous long-term electrocardiography (ECG) monitoring and may constitute a substantial improvement in AF diagnosis. Furthermore, long-term ECG monitoring is essential for studying the significance of AF in stroke pathophysiology. The enormous socio-economic consequences of stroke mean that even a modest reduction in the number of strokes would benefit society.
Primary objective:
The primary objective of this study is to determine, whether patients with increased risk of stroke have a high occurrence of AF without symptoms when studied by continuous ECG monitoring using an ILR and whether the risk of stroke can be reduced by initiating anticoagulation therapy according to pre-specified local study guidelines;
Secondary objectives:
a) Whether analysis of ECG intervals obtained from a single ECG lead (from the ILR) and from conventional ECG can predict future development of AF; and **b)** Whether the use of an expensive monitor technology – such as an ILR - for the diagnosis of AF will be cost effective in a health economics analysis due to a reduction in the number of strokes.
Study design:
Randomized, un-blinded, controlled parallel two group trial.
Endpoints:
Primary endpoint: Strokes and peripheral embolic episodes.
Secondary: AF burden, major bleeding complications, death or acute (non-elective) hospitalisation due to cardiac reasons or complications, health economy, QOL, presence of brain infarcts and white matter hyper-intensity on MRI, safety aspects of device implants, inflammation markers and

AF, genetic prediction of stroke, cardiac fibrosis on MRI, electrocardiographic markers to predict AF and stroke.

Sample size:

In total 6,000 subjects will participate: 1,500 randomised to receive an implantable loop recorder (ILR group) and 4,500 randomised to receive standard care (control group).

Summary of Subject Eligibility Criteria:

Subjects will be 70 years of age or older and will have at least one of the following diseases: hypertension, diabetes, heart failure or previous stroke (and biologically a candidate for OAC).

ILR group:

Patients randomised to the ILR group will receive the monitoring device and if AF is detected anticoagulation therapy will be given according to study guidelines.

Control group:

Patients will be treated according to standard care.

Study Duration:

The study comprises a screening period of up to 12-18 months followed by at treatment phase of a minimum of 36 months. The total study duration will therefore be approximately 5 years.

Screening and Randomisation:

Treatment: After randomisation patients allocated to the ILR group should receive the implantation as fast as possible, and preferably within 4 weeks.

Statistical Considerations:

The population is expected to have a stroke risk of 0.7%/year if AF is not present and 2%/year if AF is present. Approximately 20% are expected to have AF during monitoring for a period of more than two years, which is a conservative estimate since 10% in the ASSERT study cohort (pacemaker patients over the age of 65 with hypertension but without a history of AF) had AF in the course of 3 months ECG monitoring. As a result, there will be approximately four non-AF patients (controls) per experimental subject (AF).

We expect to have an accrual interval of two years and additional follow-up after the accrual interval of 2 years. The population will consist of patients at risk of developing stroke due to AF and patients at risk of developing stroke unrelated to AF. The AF-related strokes are expected to be reduced by oral anticoagulation therapy (hazard ratio 0.2), while strokes unrelated to AF will not be influenced by this. Thus, the overall hazard ratio is expected to be 0.65 (35% reduction of stroke in the ILR group). With a power of 80% and a type I error of 5% we need 584 patients in the ILR group. We have increased the sample size to 1.500 patients in the ILR group to increase the possibility to reach secondary endpoints. If the event rate is lower than expected follow-up can be

increased. With a follow-up of 3 years, the total number of strokes is expected to be 230 and the study is expected to continue until this number of events has been reached.

Primary Endpoint Analysis:

The principal analysis for the primary endpoint (time to one of the events in the combined endpoint) will employ the intent-to-threat principle and use a survival analysis. For each randomised group, Kaplan-Meier curves will be estimated, graphically displayed and compared using a logrank test. A co-variate adjusted analysis of the combined primary endpoint using a Cox proportional regression model will be performed as a supportive analysis. The hazard ratios and the corresponding 95% confidence intervals will be estimated. Subjects completing the study and not reaching the composite endpoint will be censored.

Secondary Endpoint Analysis:

All time-to-event secondary endpoints will be analysed similarly to the primary endpoint.

Sample Size:

Hazard rates have been estimated from previous studies, including the ASSERT study. Assuming 12-18 months recruitment period and 36 months follow-up period and a randomisation ratio of one active vs. 9 controls (where active subjects will receive an ILR and subsequent anticoagulation therapy if subclinical AF is detected) a total of 584 subjects randomised to ILR group will provide a power of 80% and a type I error of 5% we need 584 patients in the ILR group. By increasing the number of study participants in the ILR group we expect to reach statistical power to evaluate secondary endpoints. If the event rate is lower than expected follow-up can be increased. With a follow-up of 3 years, approximately 20 stroke patients in the ILR group are needed. The total number of strokes is expected to be 230 and the study is expected to continue until this number of events are reached.

Event Committee:

An event adjudication committee will classify endpoint events throughout the study.

Funding:

Research grants have been obtained from the Danish National Foundation for Strategic Research (DKK 15.6 mio), the Research Foundation for the Capital Region of Denmark (DKK 2.0 mio) and the Danish Heart Foundation (DKK 150.000). MEDTRONIC Bakken Research Center B.V.(manufacturer of the ILRs will donate an unrestricted research grant to the study covering costs related to 900 ILRs (which has a value of DKK 5.4 mio (reduced price) or DKK 22.5 mio (ordinary list price)) and a grant to cover salary for research nurses at the hospitals (DKK 1.9 mio).

Additional research grants will be applied for from private and public research foundations as well as from industry.

Protocol Signatures:

Responsible for the clinical part of the study:

Professor Jesper Hastrup Svendsen
Department of Cardiology, The Heart Centre, Rigshospitalet
Blegdamsvej 9, 2100 Copenhagen
Phone no. +45 3545 2817; E-mail: Jesper.Hastrup.Svendsen@regionh.dk

Professor Lars Køber
Department of Cardiology, The Heart Centre, Rigshospitalet
Blegdamsvej 9, 2100 Copenhagen
Phone no. +45 3545 3519; E-mail: Lars.Koeber@regionh.dk

Consultant Søren Højberg
Department of Cardiology Y, Bispebjerg Hospital
Bispebjerg Bakke 23, 2400 Copenhagen NV
Phone no. +45 3531 2578; E-mail: soeren.hoejberg@regionh.dk

Consultant Ketil Haugan
Department of Cardiology, Roskilde Hospital
Køgevej 7-13, 4000 Roskilde
Phone no. +45 4732 6000; E-mail: khau@regionsjaelland.dk

Consultant Axel Brandes
Department of Cardiology, Odense University Hospital
Sdr. Boulevard 29, 5000 Odense C
Phone no. +45 6611 3333. E-mail: axel.brandes@ouh.regionsyddanmark.dk

Professor Derk Krieger,
Department of Neurology, Bispebjerg Hospital.
Bispebjerg Bakke 23, 2400 København NV

Responsible for monitoring:

Steering committee:
c/o Professor Jesper Hastrup Svendsen
Department of Cardiology, section 2013
Rigshospitalet, Blegdamsvej 9, 2100 Copenhagen, Denmark

Members of the Steering Committee of the LOOP Study:

Professor Jesper Hastrup Svendsen, Department of Cardiology, Rigshospitalet (chairman).
Professor Lars Køber, Department of Cardiology, Rigshospitalet.
Consultant Axel Brandes, Department of Cardiology, Odense University Hospital.
Consultant Ketil Haugan, Department of Cardiology, Roskilde Hospital.
Consultant Søren Højberg, Department of Cardiology Y, Bispebjerg Hospital.
Professor Derk Krieger, Department of Neurology, Bispebjerg Hospital.
Associate Professor Christian Kronborg, University of Southern Denmark.
Associate Professor Claus Graff, Aalborg University.

Study glossary:
ILR: Implantable Loop Recorder
AF: Atrial fibrillation
CIED: Cardiac Implantable Electronic Device
Stroke: rapid loss of brain function due to disturbed blood supply to the brain.
TIA: Transient Ischaemic Attack
QOL: Quality of Life

Table of Content

Protocol synopsis:	2
Protocol signatures:	5
Responsible for the study:	5
Responsible for monitoring:	5
1. OBJECTIVES:	9
1.1 Primary:	9
1.2 Secondary:	9
1.3 Expected results:	9
2. BACKGROUND and RATIONALE:	10
2.1 Stroke and AF:	10
2.2 ECG and AF risk:	11
2.3 Health economy and QOL:	11
2.4 Rationale:	12
2.5 Clinical Hypothesis:	12
3. EXPERIMENTAL PLAN:	13
3.1 Study Design:	13
3.2 Number of Centres:	13
3.3 Number of Subjects:	13
3.4 Estimated Study Duration:	13
4. SUBJECT ELIGIBILITY:	14
4.1 Inclusion Criteria:	14
4.2 Exclusion Criteria:	14
5. SUBJECT ENROLMENT:	15
5.1 Subject Registration:	16
5.2 Randomisation and Treatment Assignment:	16
5.3 Screen Failures:	17
5.4 Re-Screening:	17
6. TREATMENT PROCEDURES:	17
6.1 ILRs:	17
6.2 ILR implantation procedure:	18
6.3 Concomitant Therapy:	19
6.4 Prescribed Heart Failure Therapy During Study Period:	19
7. STUDY PROCEDURES:	19
7.1 Assessments:	19
7.2 Subject Screening:	20
7.3 Follow-up Phase:	20
7.4 Endpoint Determination:	24
7.5 Biomarkers:	24
7.6 Strategy for management of low inclusion rate:	24
8. REMOVAL AND REPLACEMENT OF SUBJECTS:	25
8.1 Removal of Subjects:	25
8.2 Replacement of Subjects:	26
9. ADVERSE EVENT REPORTING:	26
9.1 Definitions:	26
9.2 Reporting Procedures for All Adverse Events:	27
9.3 Serious Adverse Event Reporting Procedures:	27
10. STATISTICAL CONSIDERATIONS:	27
10.1 Study Design:	27
10.2 Study Endpoints and Subsets:	28
10.3 Sample Size Considerations:	29

10.4 Interim Analysis and Early Stopping Guidelines:	29
10.5 Access to Individual Subject Treatment Assignments:	29
10.6 Planned Methods of Analysis:	30
10.7 Study Oversight:	31
11. INVESTIGATIONAL PRODUCT:	31
11.1 Loop recorder:	31
11.2 Compliance:	32
12. REGULATORY OBLIGATIONS:	32
12.1 Ethical considerations:	32
12.2 Informed Consent:	32
12.3 Independent Ethics Committee:	33
12.4 Subject Confidentiality:	33
12.5 Investigator Signatory Obligations:	34
13. ADMINISTRATIVE AND LEGAL OBLIGATIONS:	34
13.1 Protocol Amendments and Study Termination:	34
13.2 Study Documentation and Storage:	34
13.3 Study Monitoring and Data Collection:	35
13.4 Language:	36
13.5 Publication Policy:	36
13.6 Compensation:	38
14. REFERENCES:	39
15. APPENDICES:	46
Appendix A – Study procedures – summary:	47
Appendix B – Composition of Committees:	49
Appendix C – Serious Adverse Events:	50
Appendix D – Blood Samples for Genetic analysis and Blood Samples for Biomarkers:	50
Appendix E – ILR information:	51
Appendix F – Definition of composite endpoint:	53
Appendix G – Funding:	54
Appendix H – Informed consent:	54
Appendix I – Guidelines for oral information:	55

1. OBJECTIVES:

The overall objective of this project is to improve the identification and treatment of patients at risk of stroke as well as identify how significant the contribution from AF is in stroke causation when studied by a technology which identifies all AF episodes including asymptomatic. This will be pursued by three linked studies to be performed simultaneously in a randomised clinical trial where patients at risk of stroke will be divided into two groups, with one group being continuously ECG monitored by an ILR and the other one not monitored.

Primary objective:
Whether patients with increased risk of stroke have a high occurrence of AF without symptoms, when studied by continuous ECG monitoring using an ILR and whether the risk of stroke can be reduced by initiating anticoagulation therapy according to pre-specified study guidelines.

Secondary objectives:
Whether analysis of ECG intervals obtained from a single ECG lead (from the ILR) can predict future development of AF.
Whether the use of an expensive monitor technology – such as an ILR for the diagnosis of AF – will be cost-effective in a health economics analysis due to a reduction in the number of strokes.

Expected results:
The LOOP project is expected to provide results in three important areas:
1) It will document, that the detection of asymptomatic AF in high risk patients and subsequent anticoagulation therapy will benefit patients in terms of decreased mortality and/or morbidity (AF, stroke, disability etc.), decreased hospitalisation and increased QOL, not to mention psychological benefits due to being continuously monitored. Potential disadvantages are side effects caused by the ILR and medication. A demonstrated, clinically relevant benefit will have a significant impact on future patient treatment and will be included in future treatment guidelines.
2) It will show that ECG analyses obtained from ILR can be used as a means to improve identification of patients with asymptomatic AF and this will help predict which patients will develop AF during follow-up. Results will determine if this will allow the risk stratification of patients into several categories and whether patients with the highest risk need closer follow-up than those at lower risk.
3) It will document via a health economics analysis of whether or not introducing costly monitoring of patients in high risk of stroke events due to AF is cost-effective. The analysis will also show how

additional costs and cost savings will be distributed between various sectors and actors. The combined resulting documentation can then be used to inform health policy decision makers.

2. BACKGROUND AND RATIONALE:

Patients with AF, diabetes, hypertension, heart failure as well as previous stroke have increased risk of stroke. Lifestyle factors such as smoking and physical inactivity are also associated with stroke. Furthermore old age is associated with increased risk of stroke. If several risk factors are present in the same patient the risk of stroke is increased further – these individuals are called high-risk individuals with respect to stroke.

2.1 Stroke and AF:

Stroke is an exceedingly common cardiovascular disease. Out of the 16,000 people suffering annually from stroke in Denmark, 12,000 of them are first-time strokes.[1] More than 85,000 people in Denmark live with a previous stroke.[1] By 2020 120,000 individuals are expected to be living with a previous stroke, which represents a 50% increase from 2000 to 2020.[1] Stroke primarily affects the elderly. Nearly 70% of all strokes appear in people over the age of 65. Worldwide, stroke is the reason for 10% of all deaths and causes 5.7 million deaths annually.[2] The healthcare costs due to strokes in the EU were more than EUR 38 bn in 2006, accounting for 2-3% of total EU healthcare costs.[2]

AF is the most common cardiac arrhythmia. Each year (2009) more than 21,000 people in Denmark experience a first-event AF and more than 64,000 live with an AF diagnosis,[1] the incidence of AF having increased by 35-40% from 2000 to 2009, a development that is expected to continue. Thus the number of new patients with AF is expected to double in 2020 compared with 2000.[1] Other Western countries are experiencing the same type of development.[2,3] AF is associated with having a five times greater risk of stroke.[2,3] Patients with AF-related strokes have a one-year mortality of about 30% and up to 30% of all AF-related stroke victims remain permanently disabled.[2] Thus this patient group has a higher mortality rate and suffers from strokes more disabling than non-AF strokes.[2]

A systematic search for AF primarily by checking for an irregular pulse rate can identify 50-70% more AF patients than a routine strategy based on symptom-driven diagnosis.[3] Thus, putting more effort into detecting AF will mean an increase in AF diagnosis. Previous population-based AF studies have primarily used 12-lead ECG, which only detects a minimum number of cases due to the snap-shot nature of the recording. Correct diagnosis of short-lasting AF episodes is difficult, especially if the episodes are asymptomatic. Continuous long-term ECG monitoring appears to be

the ideal method to identify the true incidence of AF and to characterise its subtypes. One technology which can be used for continuous long-term monitoring is a pacemaker. This technology was used in the ASSERT Study, which recently showed that among 2,580 pacemaker patients over the age of 65 with hypertension and without a history of AF, 261 (10.1%) had device-detected asymptomatic atrial tachycardia (mostly AF) at the three-month follow-up.[4] After 2.5 years of follow-up, 51 strokes (or embolic episodes) were detected, 11 of which were detected among 261 (4.2%) of the pacemaker patients with asymptomatic AF while 40 were among the remaining 2319 (1.7%) patients without asymptomatic AF. None of the stroke patients had symptomatic AF at the three-month follow-up.[4]

A less invasive technology which can be used for continuous long-term monitoring is the implantable ECG loop recorder (ILR). This device, which measures 62 mm x 19 mm x 8 mm and weighs 15 g, is implanted subcutaneously on the chest wall without the need for electrodes in the heart chambers. The device can sample arrhythmia automatically based on specific criteria or if the patient activates data sampling due to symptoms. Currently, ILR technology provides the best possibilities for continuous monitoring of the heart rhythm for AF detection. The fact that follow-up can be performed by telemedicine is also advantageous.

Anticoagulation therapy is effective in reducing stroke incidence in patients with AF. However, at present we do not have clear evidence about how accidentally detected AF should be weighted with respect to anticoagulation therapy.

2.2 ECG and AF risk:

Most elderly patients who develop AF have structural and functional abnormalities in the atrial myocardium.[5,6] These changes may cause heterogeneous atrial activation and a shortening of the atrial refractory period, resulting in changing patterns of cardiac impulse propagation.[7] The Framingham Heart Study showed that prolongation of the electrocardiographic PR interval is associated with an increased risk of future development of AF.[8] Our research group has recently shown that both an increment of the PR interval and an increment in QTc interval are associated with an increased risk of AF (unpublished data), suggesting that the PR interval, QTc interval and AF have a shared pathophysiology. The atrial repolarisation, and more specifically the atrial effective refractory period, is probably the most important factor in the pathophysiology of AF. The ventricular repolarisation time, reflected in the QT interval, is expected to be a marker of the atrial repolarisation time, as the atria and ventricles share many of the same ion channel complexes and are thereby influenced by the same genetic variation present in an individual. Other ECG markers, such as P-wave duration, premature atrial and ventricular contractions, are also associated with future development of AF.[9] This evidence suggests that future AF can be predicted from ECG

markers measured on a single lead of the standard electrocardiogram.

2.3 Health economy and QOL:

The direct costs of stroke, including acute care and rehabilitation, are substantial. Previous studies from Denmark show that the mean cost of healthcare and social services during the first year after stroke was DKK 193,400 per person (2011 price index),[10] and that the initial severity of the stroke was the only medical factor that influenced length of hospital stays.[11] In addition to the cost of stroke treatment, rehabilitation and long term care, stroke diagnosis is associated with a significant loss of QOL for the patient.[12] Thus, interventions that prevent stroke may lead to significant cost savings that may counter balance (some of) the costs of the intervention and also improve the QOL of patients.

Several studies have explored the cost-effectiveness of various stroke rehabilitation strategies[13] and a number of studies have looked at the cost-effectiveness of secondary prevention of stroke with anticoagulation therapy in patients with AF.[14,15,16]

2.4 Rationale:

Patients at risk of stroke due to age, or presence of specific diseases may also have undetected atrial fibrillation. If atrial fibrillation without symptoms is detected by continuous ECG-monitoring by a Loop Recorder, this may potentially detect atrial fibrillation, that would not otherwise be detected, and by the monitoring of the heart rhythm the patient gets a possibility to receive anticoagulation therapy, which potentially may reduce the risk of future stroke. However, anticoagulation therapy may also have undesired side effects. Therefore, we will perform a study, where we will record both the beneficial and harmful effect in a randomized trial, and we will assess economical and quality of life aspects in a randomized trial.

2.5 Clinical Hypothesis:

2.5.1 First (major) hypothesis:

When AF is detected and relevant anticoagulation therapy is initiated in high-risk patients, a reduction in strokes will follow. Similarly, death rate and acute hospitalisations will decrease (combined end-point).

2.5.2 Secondary hypothesis:

a. ECG markers such as PR and QTc time intervals measured from distinct points on the ECG recorded from an ILR and from a 12-lead ECG can be used to predict future AF.

b. Continuous ECG monitoring with an ILR to detect asymptomatic AF and subsequent anticoagulation therapy is cost effective from a societal perspective since the cost of monitoring and therapy can be balanced against reduced costs of stroke treatment and rehabilitation, as well as improved QOL and survival.
c. Continuous ECG monitoring can detect bradyarrhythmia (requiring pacemaker) or ventricular tachyarrhythmia (requiring therapy) in these study participants.

2.5.3 Supplementary hypothesis:
Cerebral abnormalities including MRI detected white matter hyperintensity and brain infarcts (at inclusion) predict AF and recurrent stroke.
Genetic variation in genes coding for ion channel proteins are associated with development of AF and certain genetic patterns will prove to be protective against AF.
AF burden can predict development of cognitive dysfunction

3. EXPERIMENTAL PLAN:

3.1 Study Design:
This is a prospective randomized, open-label (unblinded), controlled, parallel group multi-center study performed in Denmark. Participants are recruited following identification from Danish registries as well as from outpatient clinics, and after hospitalizations. The study will recruit individuals who live in address in Region Zealand, Region of Southern Denmark or the Capital Region of Denmark. Potentially the study region will be increased to cover other parts of Denmark. Participants are randomised to either receive an ILR (ILR group) or not receive one (control group). The randomisation occurs at a ratio of 1:3 (ILR group vs. control group). The LOOP study will comply with the standards of the CONSORT Statement. [17]

After successfully completing all screening and baseline procedures subjects will be randomized in a ratio of 1 to 3 to receive an ILR or usual control. The Loop Recorder will be programmed according to study recommendations (see section 6.2).
The study flow chart is given in Appendix A and the study endpoints are defined in section 10.2.
A steering committee will oversee the conduct of the study. Composition and roles of this and other committees are described in section 10.7.

3.2 Number of Centres:
The study will be a multi-centre study performed in the south and eastern part of Denmark, where

all hospitals involved in implantations of Cardiac Implantable Electronic Devices (CIEDs) will be invited to participate. All participating centres will be involved in randomization, implantation of the ILRs as well as follow-ups.

3.3 Number of Study Subjects

Participants in this clinical investigation shall be referred to as subjects or patients. Approximately 6,000 subjects will be randomized in a ratio of 1:3 to receive an ILR (ILR group) or continue standard care regimen (control group).

It is expected, that the randomization period will last for 12 to 24 months. The decline rate is estimated as <10 %. The cross over from control to the ILR group is expected to be less than 10 %, since an ILR implant is generally assumed to require a syncope or definite clinical suspicion of arrhythmia or a wish to quantify arrhythmia burden. Subjects discontinuing from the study prematurely will remain in endpoint follow-up unless consent is withdrawn. The rationale for the sample size calculation is provided in section 10.3.

3.4 Estimated Study Duration

The study is expected to conclude after 3 years of follow-up. The steering committee will continuously monitor the overall event rate and will recommend if the follow-up period should be extended based on the number of observed primary endpoints. It is expected, that it will take approximately 1-2 years to enrol all subjects, and it is expected, that all patients will be followed for a minimum of 36 months. Based on the accumulating event rate in the total treatment group the steering committee may also recommend to alter the sample size in order to complete the trial in approximately 5 years from the start of the recruitment phase.

Randomised Clinical Trial:

This will include: recruit participants, examination of participants, randomisation, implantation of ILR and follow-up, data collection (ILR data, ECGs, blood samples, questionnaires, MRI), data management - all clinical data will be evaluated and reported, and statistical analyses of clinical data from the main trial. Invitation letters will be sent to approximately 15,000 individuals identified from registers and clinical departments. We expect that 50% (7,500) will be interested in participating, and of these 20% (1,500) are not expected to be able to participate due to the exclusion criteria. Thus, we expect to recruit 6,000 individuals to be randomised. At each of the four hospitals 1,500 patients, or about 7-8 patients per day, will be randomised (based on the assumption of 200 working-days per year).

4. SUBJECT ELIGIBILITY:

The investigators will maintain a screening log, that will include limited information about all identified potential study candidates (e.g., initials, age, sex, race, date) and outcome of the screening process (e.g. enrolled into study, ineligibility (and reason for ineligibility), or refusal to participate).

4.1 Inclusion Criteria:

To be eligible for the study subjects must fulfil the following criteria at inclusion:

- Living address in Region Zealand, Region of Southern Denmark or the Capital Region of Denmark (study subjects from other regions of Denmark who actively contact the study for participation can be included if they wish to participate)
- 70-90 years of age at the time of screening and in addition the study subject should be clinically evaluated to be biologically a potential candidate for anti-coagulation therapy

and additionally have at least one of the diseases mentioned below:

- Known diabetes mellitus (type 1 or type 2, with or without medical therapy)
- History with hypertension with or without a therapy that may reduce blood pressure (i.e., an increased blood pressure at randomization is not required)
- Heart failure: NYHA II-IV or a reduced left ventricular ejection fraction (< 0.50).
- Previous diagnosed stroke (preferably diagnosed in hospital and verified by imaging; previous TIA is not considered an inclusion criterion; a specific time interval from previous stroke to inclusion time is not required)

Concerning the four qualifying diseases the study subject is considered qualified for study inclusion if he has a history of one of the diseases (for example subject is now normotensive on life-style correction or medical therapy and similarly if blood glucose has been normalised upon life-style corrections or medical therapy).

4.2 Exclusion Criteria:

To be eligible for this study subjects must <u>not</u> meet <u>any</u> of the following criteria:

4.2.1: Uncorrected, congenital heart disease or severe valvular stenosis, obstructive cardiomyopathy, active myocarditis, constrictive pericarditis.

4.2.2: Recipient of any major organ transplant (e.g. lung, liver, heart or kidney)

4.2.3: Receiving or has received cytotoxic or cytostatic chemotherapy and/or radiation therapy for treatment of a malignancy within 6 months before randomization or clinical evidence of current malignancy with the following exceptions: Basal or squamous cell carcinoma of the skin, cervical

intraepithelial neoplasia, prostate cancer (if stable, localized disease with a life expectancy of > 2.5 years in the opinion of the investigator)
4.2.4: Known to be human immunodeficiency virus (HIV) positive with an expected survival of less than 5 years due to HIV infection
4.2.5: Renal failure treated with permanent dialysis
4.2.6: Recent (within 3 months) history of alcohol or drug abuse based on self-reporting
4.2.7: Any condition (e.g. psychiatric illness, dementia) or situation, that in the investigators opinion could put the subject at significant risk, confound the study results, or interfere significantly with the subject participation in the study
4.2.8: Known atrial fibrillation irrespective of its type (paroxysmal, persistent, long-lasting persistent or permanent)
4.2.9: Ongoing therapy with OAC or newer OAC (whereas therapy with platelet inhibitors such as acetyl-salicylic acid, clopidogrel, persantine is not considered as an exclusion)
4.2.10: Patients who have a pacemaker (including CRT-P) or ICD (including CRT-D)
4.2.11: Existing contraindication to OAC
4.2.12: On a waiting list for major surgery (cardiac, thoracic or abdominal)
4.2.13: Cardiac or thoracic surgery has been performed within 3 months from inclusion
4.2.14: Life-expectancy shorter than 6 months
4.2.15: Unwillingness to participate or patient does not understand Danish language

Study subjects who during follow-up develop one of the mentioned diseases which qualify as an exclusion criterion at baseline will remain in the study and complete the follow-up.

5. SUBJECT ENROLMENT:

Potential study subjects will be contacted by an invitation letter about the LOOP study or by direct contact and given initial basic information about the study. If patients do not respond to the initial contact they will be contacted a second time. In connection with the initial contact written information about the study is given.
If interested in participating in the study potential study subjects are asked to contact one of the participating hospitals (typically the geographically closest) and an information out-patient consultation is scheduled (inclusion visit).
At the inclusion visit the study subjects who have been invited will be informed about the study and interviewed with a focus on previous history (diabetes, heart disease, hypertension, AF etc.). Blood pressure will be measured, echocardiography and an ECG and blood sample is taken. Baseline variables including CHA_2DS_2-VASc and NYHA class score will be recorded. Individuals with AF on

the ECG will be excluded from participating and these patients will be referred for relevant treatment. Subjects will be screened for previously unknown diabetes and hypertension. If these diseases are detected the patients will be referred for further tests or therapy. Individuals who wish to participate will sign informed consent documents, complete the QOL questionnaires and will be randomised to either implantation of ILR or standard care. Subjects must personally sign and date the consent form.

Patients may have additional study tests performed (T-wave alternans test, echocardiography, MRI etc.).

For the purpose of this trial the following definitions will apply:

Registration

A subject has signed the informed consent form and has been assigned a subject identification number generated by the computer generating code (but not yet randomized)

Randomization

A subject has been assigned to one of the two treatments and has been assigned a randomization number.

Enrolment

A subject is considered enrolled once the subject is randomized and the ILR implantation (or control) has been performed.

The subject identification number assigned during registration (section 5.1) will be used to identify a subject throughout the trial and must be used on all study documentations related to that subject. The subject identification number must remain constant throughout the entire trial; it must not be changed at any time at rescreening, enrolment or randomization.

5.1 Subject Registration:

After the provision of informed consent each subject will be registered in a web-based register. All entries up to and including randomization are performed at the participating centre. The main database is physically located at Rigshospitalet. Subsequent follow-up visits can be entered at the participating hospital where the patient is being followed. Subject initials and date of birth as well as limited other information will be required for registration, including for example subject initials, date of signed informed consent, subject gender, subject ethnicity and subject weight.

5.2 Randomisation and Treatment Assignment

Subjects, who successfully complete the screening assesment will be randomized into the study. Eligible patients will be identified from a centrally performed registry draw, and from this the

individual participating centre will get patients for randomization. A computer generated randomization scheme will be generated to assign subjects to treatment, and those who fulfil the eligibility criteria (section 4) will be randomized in a ratio of 1:9 to receive an ILR or to be a control, respectively. The investigator (or assigned designee) will access the central computer and obtain an assignment code at the randomization visit. The following information at a minimum will be required for randomization:

- Subject identification number
- Date of birth
- Confirmation, that the subject meets the eligibility criteria

This information will be entered into the electronic case report form. Subject must be randomized within 30 days of being identified at the referral hospital.

5.3 Screen Failures:

Registered subjects, who are ineligible for the study based on screening assessments will be considered screen failures and entered as such into the registration computer. The following information will be required:

- Subject identification number
- Date of birth
- Reason for failing screening
- Date of last screening assessment

5.4 Re-Screening:

Registered subjects, who do not meet all eligibility criteria on the first screening occasion may be rescreened for the study at a later time. Such subjects may begin screening procedures again two weeks after the date of the last screening assessment. Rescreened subjects will retain the originally assigned subject identification number: However, the rescreening attempt will need to be entered into the registration computer.

6. TREATMENT PROCEDURES:

Implantation of a Loop Recorder will be performed according to local standards and within four weeks of randomization.

6.1 Loop Recorder:

Loop Recorders from Medtronic (Reveal LINQ or newer comparable devices) with possibility

of remote monitoring are planned to be used in this trial. Information on specific Loop Recorder data can be found in Appendix F.

6.2 Loop Recorder implantation procedure:

Preoperative biochemical parameters as required by local guidelines (which may include variables such as INR, blood cells, creatinine, sodium, potassium).

The device is implanted at the hospital using standard sterile technique. The implant procedure is not necessarily performed in an operation facility. An intravenous injection line (such as Venflon) is optional, but is inserted if i.v. antibiotics are given.

Medication in relation to surgery:

Antibiotics are given according to study guidelines. It is suggested to give at least one dose of i.v. cefuroxime (Zinacef) 1.5 g or dicloxacillin (Diclocil) 0.5-1.0 g immediately prior to surgery or oral dicloxacilling (Dicillin) 0.5-1.0 g at least one hour prior to implant. It is optional to give additional antibiotics. In case of allergy to penicillin alternative antibiotics is given according to local guidelines.

Sedatives are not recommended. Local anaesthesia is given according to each centre´s local guidelines.

The surgical procedure:

The Loop Recorder is positioned subcutaneously on the left side of the chest using supplied incision and insertion tools. Measurement of the device orientation and securing optimal electrocardiogram is optional. At each centre the implantation of the Loop Recorder is performed by either an experienced cardiologist or a medical doctor under general supervision of the local experienced cardiologist

Post operative monitoring:

After the implant the study subject is kept for not less than one hour, and after this the patient can be discharged from hospital. The patients will receive advice with respect to skin inspection in the surgical region, treatment of local pain after surgery and follow-up.

Loop Recorder programming:

The loop recorders should be programmed according to study guidelines, and details are specified in Appendix E.

Remote follow-up:
Study participants will perform automatic daily remote transmissions since the device has an antenna.

6.3 Concomitant Therapy:
Throughout the study investigators may prescribe any concomitant medications or treatments necessary to provide adequate supportive care. Information on concomitant therapy will be collected on the appropriate case record form. It is possible for the patient to participate in another trial, both including devices (such as stents) or medical therapy.
If the ILR monitoring detects severe bradycardia, ventricular tachyarrhythmia or other arrhythmia these will be dealt with according to clinical guidelines.

6.4 Prescribed antithrombotic and anticoagulation therapy and prescribed heart failure therapy during the study period:
All subjects (both ILR group and control patients) should receive optimal medical therapy for their inclusion criteria disease (hypertension, diabetes, heart failure or stroke), including ACE inhibitor or angiotensin II receptor blocker, betablocker and/or aldosteron antagonist. The standard therapy for heart failure, atrial fibrillation and hypertension are described in the guidelines from the Danish Society of Cardiology which can be downloaded from the web page: www.cardio.dk. The standard regimen for stroke patients is described in the Danish Reference Programme 2013 (Dansk Apoplexi Selskab).

7. STUDY PROCEDURES:
A tabular schedule of study procedures is given in Appendix A. Written informed consent must be obtained before performing screening procedures. The investigator is responsible for ensuring that all assessments are performed according to the protocol and that the appropriate data are recorded in the case report forms. Missed visits, test(s), that are not done and examinations, that are not conducted, must be reported on the case report forms.

7.1 Assessments:
<u>CHADS-VASc score:</u> To establish the risk of stroke.
<u>NYHA functional classification:</u> The severity of heart failure will be evaluated by the investigator or

the designee at the respective study visits. If possible a subject should be evaluated by the same person throughout the study.

Concomitant therapy: Information on concomitant therapy and medication will be recorded in the case report forms on study visits.

Adverse events: Information on all adverse events (AEs), that occur from study day 1 after randomization will be recorded in the appropriate case report forms (section 9.2). All serious adverse events (SAEs) will be reported to the study office within one working day of discovery, and a serious adverse event form must be completed (section 9.3). Bleeding complications will be registered according to the suggestions by the International Society for Thrombosis and Haemostasis (Schulman and Kearon, 2005).

Deaths: For the efficacy analysis of mortality, the ECC will review all deaths and classify the events as cardiovascular or non-cardiovascular as described in Appendix H. Cardiovascular deaths will be further classified as sudden and non-sudden based on a time criteria, as described in Appendix H. Adjudicated cause of death as well investigator-reported cause of death will be entered into the clinical database.

7.2 Subject Screening:

During screening each subject will be evaluated for inclusion in the study. All laboratory assessments scheduled for screening will be done by the local laboratory. The following procedures, screening evaluations and assessments will be done once within 21 days prior to randomization:

- Obtaining or verifying informed consent
- Medical history, including previous, surgical procedures
- NYHA functional classification
- CHADS-VASc score
- Physical examination, including height, body weight
- Concomitant therapy
- Blood samples for routine biochemical testing, plasma troponines, p-BNP and for storage in the study related research biobank. After analysis of the material from the study related research biobank surplus biological material is transferred to a biobank for future research.
- ECG

If laboratory samples are missing, destroyed, unable to be analysed or results are considered and documented by the investigator as being obviously incorrect, a subject may be re-tested.

7.3 Follow-up Phase:

7.3.1. The main study:

The ILR devices will be implanted over the course of 12(-18) months (7-8 implantations per week in each of the centres), starting at the beginning of 2014.

- Participants in the ILR group will be checked at in-hospital visits at 12, 24 and 36 months after ILR implantation. Between in-hospital visits they will perform daily automatic ILR remote telemedicine transmissions. Subjects will be contacted if transmission are not received and system malfunction will be corrected. Arrhythmias detected by the ILR will be treated according to current guidelines and specific study rules. Follow-up will continued until the end of battery life or until death occurs. At in-hospital visits QOL and MOCA forms will be filled in.
- Participants in the control group will be seen only at inclusion and after the 3 year follow-up. During this period they will be seen by their general practitioner. Between the scheduled visits QOL forms will be sent by mail. MOCA forms will be filled in on the final follow-up.

Additional visits are made at the discretion of the caring physician. Throughout the study the investigator or study nurse/coordinator should continue to provide appropriate medical care.

The following assessments and procedures will be performed at specified time points during the study (for details please see Appendix A, section a and b):

- NYHA functional classifications: Months 12, 24, 36.
- Remote Loop Recorder readings: daily
- Vital signs (BP, HR): During visits
- Blood sample: At randomization.
- Concomitant therapy: At clinical visits.
- AES: At clinical visits.
- Assessment of Quality of life (QOL) and cognitive function : At randomization, months 12, 24 and 36.
- CHADS-VASc score
- Hospitalisations

The main study will register strokes or peripheral embolic episodes (combined primary endpoint).

<u>Endpoints:</u>

<u>Primary:</u> clinical and adjudicated stroke or peripheral embolic episode (i.e. events which presumably can be avoided by a relevant OAC therapy)

<u>Secondary:</u> Major bleeding complications (according to Schulman and Kearon. Journal of Thrombosis and Haemostasis, 2005; 3: 692–694), death or acute (non-elective) hospitalisation due

to cardiac reasons or complications, cerebral haemorrhage, time to AF, AF burden, other arrhythmia (bradyarrhythmia and ventricular arrhythmia), QOL, presence of brain infarcts and white matter hyperintensity on MRI (semiquantitative evaluation), cardiac chamber dimensions and function as well as fibrosis in chamber walls as assessed by MRI, safety aspects of device implants, inflammation markers, genetic prediction of stroke.
In the entire study cohort we will prospectively collect data on strokes, peripheral emboli, hospitalisations and death as well as perform QOL measurements using validated instruments (SF-36 and EQ-5D-5L) and cognitive function (using the MOCA instrument). A biobank is established (for: a) genetic screening (of genes relevant for cardiac arrhythmia and stroke), b) inflammation markers, c) heart hormones).In the control group (who are only seen at inclusion and end of study) we will perform a registry search for death, strokes and hospitalisations every 6-12 months.

7.3.1 The ECG study:
The ECG computer analysis study will collect periodic data from the loop recorders (ILR group) during sinus rhythm and during episodes of AF (both patient-activated and automatic sampling). Patients in the ILR group will be encouraged to make a monthly patient-activated sampling after five minutes of rest (most likely in sinus rhythm). Standard 12-lead ECGs will be obtained at baseline (ILR and control groups) and also annually in the ILR group. ECG markers predictive of future AF will be derived from the recordings.

Endpoints:
Primary: Incident AF and the association between chronological developments of ECG markers measured from single lead loop recorders and incident AF to identify which patients fall into low or high risk AF groups.
Secondary: Calculation of daily AF burden (ILR group) to refine quantification of episodes beyond merely presence/absence and to guide decisions regarding the need for anticoagulation.
Relationship between AF burden and development of ECG markers.
Additional endpoints:
Incidence rate and timing of new onset AF to identify the necessary duration and regularity of ECG monitoring to reliably identify change in status.
Throughout the ECG study we will also monitor the frequency of symptom triggered ILR transmissions, the proportion of falsely detected AF episodes by ILR as well as the number of planned ECG samplings and data transmissions from ILR devices which are not received.
Bradyarrhythmia and ventricular tachyarrhytmia.

Bleeding complications.

7.3.2 The health economic study:

The health economic analysis is appended to the analysis of the randomized clinical trial.

Endpoints:

Changes in the use of healthcare resources and costs, i.e. hospital treatment, general practitioner services, medical specialists, pharmaceuticals, rehabilitation, nursing home and quality-adjusted life-years (QALYs).

Individual personal data on resource use and health outcomes will be collected prospectively during the trial or from national registers, which also continuously record healthcare consumption.

The framework for the analysis is a cost-utility analysis where the mean differences in costs and QALYs between the intervention and the control group is expressed as a ratio that shows the cost per QALY gained from the introduction of ILR monitoring and subsequently AF management. All costs will be estimated from a societal perspective.

Resource use data in relation to the implantation of the loop recorder, ILR data transmission and analysis, as well as follow-up visits, will be recorded prospectively. The registration will include noting the amount of time staff spend during pre-operative evaluation, procedures (e.g. blood tests, electrocardiogram), utensils, anaesthetics, sedatives, antibiotics and hospital stay for each participant. In order to estimate costs, information about salaries, the cost of utensils, pharmaceuticals and equipment will be collected from the hospital finance department.

Data on the participants' use of inpatient and outpatient hospital care and use of emergency departments, general practitioners and other private practising medical specialists, physiotherapists, chiropractors, chiropodists and psychological counsellors, as well as prescription drugs, will be collected from the National Patient Register, the National Insurance Service Register, and the Register of Medicinal Product Statistics. It is assumed that costs of healthcare consumption unaffected by the intervention will cancel out due to randomisation. Furthermore, it is assumed that all AF management costs will be captured in the register data.

Hospital treatment will be valued with official hospital charges (diagnosis related group (DRG) charges) provided by the National Board of Health. Special attention to the relevance of using DRG charges will be considered.[21] Services from private practising providers will be valued according to the prevailing fee schedules that are agreed upon between the providers and the regional health authorities. For the valuation of general practitioner services an annual amount will be added to take into account that general practitioners are paid by a combination of capitation and fee-for-service. Furthermore, patients' co-payments will be added. Data on the participants' use of

prescription drugs will be collected from the Register of Medicinal Product Statistics. These drugs will be valued according to the pharmacy sales prices.

Information about the participants' use of services provided by the municipalities, e.g. community nurse visits, personal care and practical assistance at home, nursing home, respite care and rehabilitation, will be collected from the participants' municipality of residence. Unit costs of these services will be estimated based on statistics on wage rates and databases that record the cost of providing home care, (e.g. The Free Choice Database (*Fritvalgsdatabasen*)), which is administered by the National Board of Social Services. All costs will be converted to a specific year price level using the consumer price index and other relevant price indices.

QALYs will be estimated with the use of the EQ-5D-5L instrument.[12] Participants will fill in the EQ-5D-5L questionnaire at baseline (before operation) and every second year (mailed to control group). Each health state is converted to a social tariff (utility score) from a representative sample of the Danish population.[22] This will be used to construct each participant's utility profile. A straight line relation between each of the observed utility scores will be assumed. The number of QALYs experienced by each participant will be calculated as the area under the participant's utility profile.

Descriptive analyses will be used to describe the mean QALYs, resource use and costs per participant in both arms of the trial. Differences in QALYs, resource use and costs between the two groups after 24 months of the study will be based on the trial data. Confidence intervals of 95% for differences between the two groups will be computed based on bootstrap re-sampling with 5,000 replications of the trial data.

For the cost-effectiveness analyses, the incremental cost-effectiveness ration (ICER) will be estimated on the basis of the mean difference in costs relative to the mean difference in QALYs. Uncertainty about the ICERs will be analysed using bootstrapping and presented in a cost-effectiveness plane. A cost-effectiveness acceptability curve will be generated to represent the probability that loop recorders are cost-effective compared with usual treatment. Estimates of the costs per QALY gained from ILR will be compared to similar estimates of rehabilitation after stroke, AF management as well as other cost-effectiveness estimates in other treatment areas.

Study nurses will register all contacts between study subject and study nurse in the hospital files to be included in the cost evaluation.

7.3.3 The MRI study:

Patients with risk factors for stroke frequently have asymptomatic previous brain infarcts.[19] Age, hypertension and diabetes predispose to white matter hyperintensities, which is associated with cognitive disorders, balance and mood disorders.[20] In a subset of the study participants (approximately 500 subjects) we will perform magnetic resonance imaging (MRI) of the brain upon

study entry to detect previous brain infarcts (semi-quantitative assessment) and white matter hyperintensities (semi-quantitative assessment). MRI scans will be performed without contrast injection. In the patients in whom stroke occurs during follow-up we will perform a second MRI close to the time when stroke is diagnosed. In the subjects who had a first MRI scan performed a final MRI will be performed at the end of the study (approximately 4-500 subjects). MRI scans of brain are performed with 3 sequences: FLAIR , DWI and Gradient Echo sequencing. In a subset of participants we will perform an MRI scan of the heart with Gadolinium contrast to measure chamber dimensions, wall-motion and late enhancement (fibrosis) (approximately 200 subjects).

7.4 Endpoint Determination:

7.4.1 Definition of Endpoints:

Details regarding the definition of the components of the composite endpoint are provided in Appendix F. In this section definitions of the cerebrovascular endpoints are given.

7.4.2 Data Collection:

Endpoint data will be collected on specific case report forms. All supporting documentation will be provided to the endpoint committee by the investigators during the course of the trial. These data will be provided to the endpoint adjudication committee for review and confirmation of events as outlined in their charter.

7.5 Genetic analysis and Biomarkers:

In addition to testing the safety and effectiveness of medical treatment, biomarkers of disease severity will be identified and analyzed. At randomization 3 types of blood whiles will be collected (whole blood sample, EDTA while sample, and serum sample). Please see Appendix D for handling of blood samples.

7.6 Strategy for management of low inclusion rate:

1) Too low patient recruitment due to healthcare professionals or logistics: We will continuously monitor patient inclusion each month per centre. If a centre does not recruit the expected number of patients we will recruit a new centre. In the interim period the other centres will increase their recruitment.

2) Too low patient recruitment due to patient preferences: We conducted a small survey asking potential candidates if they would participate if asked, and patients were positive. A logbook will be

used to list and identify reasons why patients decline to participate. If better information can increase the participation rate we will improve the quality of the information. Otherwise additional letters of invitation will be sent.

3) Patients cannot perform telemedicine transmissions: Rigshospitalet is currently performing another study with ILRs in elderly patients with valve disease (aortic stenosis). These patients are fully capable of performing telemedicine transmissions. In the event of problems, study nurses will guide the patients by telephone.

7.7 Study guidelines dealing with definition of AF:

Arrhythmia which the device has sampled and categorised as AF and which is afterwards:

- confirmed as AF by a study investigator and
- has a duration of more than 2 minutes (please note that this is not the same as AF which requires anticoagulation therapy)

After new AF has been diagnosed the patient will continue unaltered arrhythmia monitoring as previously performed. The device requires 2 minutes for AF detection.

7.8 Study guidelines how to treat AF detected by ILR:

When AF is detected by the loop recorder and the episode is verified by an investigator and has a qualifying duration (i.e. an episode which has a duration $\geq$ 6 minutes), the patient is given advice to start therapy with one of the newer OAC (preferred strategy) or conventional OAC according to local routine if the treating physician considers that the patient is suitable candidate for this therapy.

It is recorded if the patient actually will receive OAC.

7.9 Study guidelines how to treat AF detected by other methods (also relevant for the control group):

When AF is diagnosed in the control group or in the ILR group by alternative non-ILR methods (including monitoring during hospitalisations, Holter, ECG, etc) patients are recommended to start anticoagulation according to the same guidelines as mentioned in the section above (section 7.8). If AF is diagnosed on a standard 12-lead ECG it is recommended to have a Holter recording performed.

7.10 Risks and adverse events related to study participation:

Study patients randomised to the ILR group may have local complaints due to surgery and the insertion of a device subcutaneously. They will have a small scar after surgery. There is a small

risk of infection in the region of surgery. They may have diagnosed atrial fibrillation (which is the purpose of the study) without symptoms and experience anxiety due to knowing that this was detected even though it does not give symptoms. They may start anticoagulation therapy and this may give side-effects such as bleedings, which would not have occurred if they had not participated in the study. EGG artefacts may be diagnosed as atrial fibrillation. Patients may experience that they become more focused on their health situation due to frequent data transmissions.
Patients in the control group may feel unhappy since they are not followed as closely as they had hoped.

8. REMOVAL AND REPLACEMENT OF SUBJECTS:

8.1 Removal of Subjects:

A subject has the right to withdraw from the study at any time, for any reason, without prejudice to his or her future medical care by the physician or at the institution. Any subjects who withdraws consent to participate in the study will immediately be removed from further treatment and/or study observations on the date of request. The investigator has the right to withdraw the subject from the study, if any of the following occurs:

- Significant illness caused by the Loop Recorder
- Refusal by the subject to continue observations
- Decision by the investigator, that termination is in the subjects best medical interest
- Loss to follow-up

Should a subject (or legally acceptable representative) request or decide to withdraw from the study, all efforts will be made to complete and report the observations as thoroughly as possible up to the date of withdrawal. All information should be reported on the applicable case report form. A complete, final evaluation and assessment (section 7.4) should be made at the time of the subject´s withdrawal. The end of study case report form will be completed with an explanation for the withdrawal. If the withdrawal of a subject is due to an adverse event, follow-up visits should be scheduled until the adverse event has resolved or stabilized. Unless consent has been withdrawn, follow-up date on death and hospitalizations will be collected until study termination.

However, unless specifically requested by the patient, the patient will be followed for the primary endpoint until end of study.

8.2 Replacement of Subjects:
Randomized subjects will not be replaced if discontinued.

9. ADVERSE EVENT REPORTING:

9.1 Definitions:

9.1.1 Adverse Events:
An adverse event is an undesirable, medical occurrence (sign, symptom, or diagnosis) or worsening of a pre-existing medical condition (e.g., diabetes, rheumatoid arthritis), that occurs in a subject after the initiation of the study, whether or not considered to be related to the implantation of the ILR. Worsening of a pre-existing medical condition is an increase in its severity, frequency, or duration. Elective hospitalizations for pre-existing conditions (i.e., due to any pre-existing systemic illness or elective surgery) are not adverse events. Abnormal laboratory values should not be reported as adverse events: However, any clinical consequences of such abnormal values should be reported as adverse events.

9.1.2 Serious Adverse Events:
A serious adverse event is defined by regulatory agencies as one that suggests a significant hazard or side effect, regardless of the investigators or sponsors opinion on its relationship to the investigational product. This includes, but may not be limited to, any event, that (at any dose):

- Is fatal
- Is life threatening (places the subject at immediate risk of death)
- Requires in-patient hospitalization or prolongation of existing hospitalization
- Is a persistent or significant disability/incapacity

A hospitalization meeting the regulatory requirement for “serious” criteria is any in-patient hospital admission, which includes a minimum of an overnight stay in a health care facility. Any event, that does not exactly meet this definition yet, which, in the investigators opinion, represents a significant hazard, can be assigned “other significant hazard” regulatory reporting serious criteria. Additionally, important medical events, that may not be immediately life-threatening or result in death or hospitalization, but which may jeopardize a subject or require intervention to prevent of the outcomes listed above, or result in urgent investigation, may be considered serious. Examples include allergic bronchospasm, convulsions or blood dyscrasia. Serious Adverse Events will be

reported based on patients information to study personal or monthly registry retrievals (“grønt system”) and will be filed in CRFs by the local study team.

9.2 Reporting Procedures for All Adverse Events:

All adverse events occurring after randomization observed by the investigator or reported by the subject (whether or not attributed to the investigational product), will be reported on the electronic case

report form. Medically significant adverse events considered related to the investigational product by

the investigator or the sponsor will be followed until resolved or considered stable. The following attributes must be assigned by the investigator:

Description: Dates of onset and resolution and action taking. The investigator may be asked to provided follow-up information. All adverse events, serious or not, that result in a subjects permanent withdrawal from the study must be reported in the CRF. The end-of-study case report form will be completed, giving details of the withdrawal, and, if needed, the General Comments case report form will also be completed.

All deaths occurring in study must be recorded, and cause of death should be investigated. This includes deaths until determination of the study or a maximum of 60 months since enrolment of the subject, whichever is earlier.

It will be left to an investigators clinical judgement as to whether or not an adverse event is of sufficient severity to require a subjects removal from treatment.

9.3 Serious Adverse Event Reporting Procedures:

All serious adverse events must be reported in the electronic CRF within one working day of discovery or notification of the event. Initial serious adverse event information and all amendments or additions must be recorded on a Serious Adverse Event Report form.

Description of serious adverse event reporting is included in Appendix C.

10. STATISTICAL CONSIDERATIONS:

10.1 Study Design:

This is a prospective randomized, open-label (unblinded), controlled, parallel group multi-center study performed in Denmark. Patients are recruited following identification from Danish registries

as well as from outpatient clinics, and after hospitalizations. The study will recruit individuals who live in the Capital Region of Denmark, Region Zealand or the Southern Region of Denmark. The study is expected to conclude when 230 strokes have occurred or 6,000 study subjects have been followed for at least 36 months, which is expected to be approximately 5 years after enrolment of the first subject. Subjects meeting the eligibility criteria will be randomized to either receive an ILR (ILR group) or not ie, continue standard care (control group). The randomisation occurs at a ratio of 1:3 (ILR group vs. control group) as described in section 5.2. The LOOP study will comply with the standards of the CONSORT Statement.[17] The study design is further described in section 3.

10.2 Study Endpoints and Subsets:

10.2.1 Primary Endpoint:

Efficacy (time to at least one of the components of the combined primary endpoint):

- clinical and adjudicated stroke or
- clinical and adjudicated peripheral embolic episode

10.2.2 Secondary Endpoints:

Efficacy evaluated by:

- death due to cardiac reasons/complications
- death due to any reason
- acute (non-elective) hospitalisation due to cardiac reasons or complications requiring an overnight stay in hospital
- Development of TIAs
- Development of AF and AF burden
- Development of other cardiac arrhythmia
- ECG markers ability of predicting AF
- Health economic costs
- Change from baseline QOL and Quality Adjusted Life Years
- MRI: Change in occurrence of brain infarcts and white matter hyper-intensity
- MRI: Left atrial size, and fibrosis and the association with stroke and AF
- Safety aspects (such as infections) of ILR implants
- Major bleeding complications (as defined by Schulman and Kearon, 2005)
- Inflammation markers and cardiac hormones and their prediction of AF and stroke
- Cognitive dysfunction evaluated by MOCA forms
- Genetic prediction of AF and stroke

- Echocardiographic prediction of AF and stroke

10.2.3 Subsets:

Efficacy Analysis Set:

Efficacy analysis will be performed on the intent to treat (ITT) subject set, defined as comprising all subjects randomized. Efficacy analysis will include all available follow-up data from all randomized subjects. In addition sensitivity analysis may be carried out based on the un-treatment subject set, which is defined as subjects, who will randomize to or receive an ILR, and the controls not receiving an ILR.

10.3 Sample Size Considerations:

We have performed sample-size calculations and we are planning a randomised study of 6,000 patients, 1,500 of whom (1 active vs. 3 controls) will receive an ILR and subsequent anticoagulation therapy if subclinical or clinical AF is detected. The population is expected to have a

stroke risk of 0.7%/year if AF is not present and 2%/year in patients with AF.[18] Approximately 20% are expected to have AF during monitoring for a period of more than two years, which is a conservative estimate since 10% in the ASSERT Study had AF in the course of 3 months of monitoring.[4] As a result, there will be approximately four non-AF patients (controls) per experimental subject (AF).

We expect to have an accrual interval of two years and additional follow-up after the accrual interval of 2 years. The population will consist of patients at risk of developing stroke due to AF and patients at risk of developing stroke unrelated to AF. The AF-related strokes are expected to be reduced by oral anticoagulation therapy (hazard ratio 0.2), while strokes unrelated to AF will not be influenced by this. Thus, the overall hazard ratio is expected to be 0.65 (35% reduction of stroke in the ILR group). With a power of 80% and a type I error of 5% we need 584 patients in the ILR group. We have increased the sample size to 1,500 patients since this will give us power to reach the secondary endpoints. If the event rate is lower than expected follow-up can be increased. With a follow-up of 3 years, approximately 20 stroke patients in the ILR group are needed. The total number of strokes is expected to be 230 and the study is expected to continue until this number of events has been reached. Participants for the project will be recruited from a random sample of individuals registered in the Danish Civil Registration System and the National Patient Registry who meet the inclusion criteria.

10.4 Interim Analysis:

The likely hood of observing a statistically significant beneficial treatment effect is dependent on the event rate in the trial. When approximately 1/3 of the subjects have completed one year of follow-up this event rate will be discussed by the steering committee in order to decide, if the study should continue as planned. The steering committee has the right to increase sample size or prolong follow-up if needed.

10.5 Access to Individual Subject Treatment Assignments:

Individual subject treatment assignments will be given to the steering committee if requested. Un-blinded (presented as group A and B) results, including aggregate and subject level data, can be presented to the steering committee for safety data reviews.

10.6 Planned Methods of Analysis:

10.6.1 General Approach/Considerations:

Baseline characteristics, demographics, pre-existing conditions, previous illnesses, and possible prior treatments will be summarized for each treatment group. Subjects disposition and reason for discontinuation will be summarized for each group. The primary and secondary efficacy endpoints are timed to event variables and survival techniques will be utilized to perform statistical estimation and hypothesis testing. Concomitant medications will be summarized for each treatment group and compared between groups to access their potential impact on the primary endpoint.

10.6.2 Analysis of Key Study Endpoints:

10.6.2.1 Primary Endpoint:

The principle analysis for the primary endpoints will employ the intend-to-treat principle, and use a survival analysis. For each treatment group, Kaplan-Meier curves will be estimated, graphically displayed, and compared using a two-sided logrank test at a 0.05 significance level. A covariate-adjusted analysis of the primary endpoint using a Cox proportional hazards model will be performed as a supportive analysis. The hazard ratio and its corresponding 95% confidence interval will be estimated. The selection of covariates will be defined prospectively in the statistical analysis plan prior to study commencement. Subjects withdrawn from the study early (other than for withdrawal of consent) will be followed for potential development of the primary endpoint.

Subjects completing the study and not experiencing the composite event will be censored.

10.6.2.2 Secondary Endpoints:

See section 10.2.2.

The secondary endpoints will be tested at a significance level of 0.05. Within the first two secondary endpoints the type 1 error rate associated with a multiplicity of comparisons will be controlled according to the Hochberg adjustment. The Quality of Life endpoint will be tested at the 5% significance level. For the time-to-event variables, analysis similar to the one employed for the primary endpoint will be used. The censoring mechanism will also be similar to the one used for the analysis of the primary endpoint. Quality of Life score will be analysed as repeated measures analysis of covariance mixed effects model using baseline score as a covariate.

10.6.2.3 Safety Endpoint:

Adverse events: Subjects incidence rates of all adverse events will be tabulated by system class, preferred term, severity, and relationship to investigational product. Tables and/or narratives of “on-study” death, and serious and significant adverse events, including those causing early withdrawal, will also be provided.

The following adverse events will be asked for at all visits and registered specifically in the CRF: ILR infection.

10.6.3 Additional Analyses:

Laboratory values: Laboratory values will be summarized over time by assessment visit using number of observations, mean, standard deviation, median, minimum and maximum. Graphical representations of aggregate data may also be presented. If appropriate, shift tables or scatter plots comparing the “worst” on-study laboratory value with a baseline value may be provided.

Vital signs: Blood pressure and heart rate will be summarized for each treatment group by calculating the mean standard deviation, median, minimum and maximum.

10.7 Study Oversight:

10.7.1 Steering Committee:

A steering committee comprising experts in the cardiovascular field will provide oversight and advice to ensure the most efficient conduct and execution of the trial. The steering committee will consist of external physicians with experience and expertise in the design and conduct of cardiovascular clinical trials and will have representations from the working group on pacing and

electrophysiology of the Danish Society of Cardiology, as well as representatives from Danish Stroke Neurologists. The main focus of the steering committee will be on the medical, ethical and scientific integrity of the study and on its timely completion. The steering committee will consider sample size re-examination in the eventuality at the observed, blinded pooled event rate for the primary endpoint false below its assumed estimate in the design stage. Steering committee members may be investigators in the study. To facilitate the communication of results from the study, a publication committee will be formed from the steering committee at an appropriate time, consisting of members of the steering committee and of others, who have contributed significantly to the study.

10.7.2 Endpoint Committee:

An endpoint classification committee (EC) will be formed prior to study commencement to adjudicate the primary endpoints in a blinded fashion (see Appendix B and F). A neurologist and a cardiologist are appointed in the endpoint committee.

With respect to correct classification of recorded AF events an “AF adjudication committee” will be established among the cardiologists in the steering committee. “The AF adjudication committee” will be composed of two cardiologist (rotation among SC members will occur). If these two cardiologists disagree on an adjudication a third cardiologist will be involved.

11. INVESTIGATIONAL PRODUCT:

11.1 Loop recorders:

The Loop recorder in this study is the Reveal LINQ (Medtronic) or newer devices with similar functionality. The device is described in details in appendix E.

The devices are equipped with remote monitoring equipment.

11.2 Compliance:

All patients randomised to Loop recorder should undergo implantation. However, the number of patients randomised but not receiving a Loop recorder will be registered as the number of explanted Loop recorders will.

12. REGULATORY OBLIGATIONS:

12.1 Ethical considerations:

It is currently not known how asymptomatic AF should be treated. Only when all (“the majority of”) AF is diagnosed the scientific and clinical problem about how to deal with asymptomatic AF can be addressed properly. One approach in this would be to perform continuous long-term monitoring of patients at risk. Therefore the LOOP study will answer clinically relevant questions and is based on this background.
In the present study patients will be randomised to either receive an ILR or not. For patients randomised to the control group, there is very little burden by the study since there is no placebo treatment. Patients randomised to ILR have the potential advantage of having AF detected and subsequently receiving appropriate anticoagulation therapy, which may turn out to be beneficial. However, such treatment may also cause potentially serious side effects. An implanted ILR may become infected. It may be disadvantageous for patients QOL to be instrumented with an implantable device due to local side effects. On the other hand, patients may experience that it gives them a feeling of safety.

This study will require approval from the Regional Danish Committee on Biomedical Research Ethics, as well as allowance from The Danish Data Protection Agency. The Danish Medicine Agency has been asked if application for permission to perform the study was necessary , and the Agency has answered that an approval of the LOOP study was not necessary.

Overall the study addresses an important clinical problem and if the trial turns out positive it will improve therapy for a large number of patients. Since the socio-economic costs in therapy and care related to stroke are enormous the results from the study may also be important for society. Therefore, we consider the risks we impose on patients are acceptable.

12.2 Informed Consent:

An informed consent form is provided in Appendix H. Guidance for oral information of participants is given in Appendix I.
Before a subject´s participation in the trial, the investigator is responsible for obtaining written informed consent from the subject after adequate explanation of the aims, methods, anticipated benefits, and potential hazards of the study and before any protocol-specific screening procedures or any investigational products are administered. The potential benefit and hazard associated with participation in the study is described in the informed consent form (Appendix H). If the subject agrees, it is recommended that the investigator informs the subjects primary care physician of his/her participation in the clinical trial.

The acquisition of informed consent should be documented in the subjects medical records, and the informed consent form should be signed and personally dated by the subject, and by the person who conducted the informed consent discussion (not necessarily an investigator). The original signed informed consent form should be retained in accordance with institutional policy, and a copy of the signed consent form should be provided to the subject or the subject's legally acceptable representative.

If a potential subject is illiterate or visually impaired, the investigator must provide an impartial witness to read the informed consent form to the subject, and must allow for questions. Thereafter, both the subject and the witness must sign the informed consent form to attest that informed consent was freely given and understood.

12.3 Independent Ethics Committee:

A copy of the protocol, proposed informed consent form, other written subject information, and any proposed advertising material must be submitted to the Ethics Committee for the Capitol Region of Denmark. The investigator must submit and, when necessary, obtain approval from the Ethics Committee for all subsequent protocol amendments and changes to the informed consent document.

12.4 Subject Confidentiality:

The investigator must ensure that the confidentiality of a study subject is maintained. On the case report forms or other documents mailed to the study office, subjects should be identified by their initials and a subject study number only. Documents that are not for submission (e.g. signed informed consent forms) should be kept in strict confidence by the investigator.

In compliance with Federal regulations/ICH-GCP Guidelines, it is required that the investigator and institution permit authorized representatives of a monitoring company, and the EC, direct access to review a subjects original medical records for verification of study-related procedures and data. Direct access includes examining, analyzing, verifying, and reproducing any records and reports that are important to the evaluation of the study. The investigator is obligated to inform and obtain the consent of a subject for named representatives to have access to his/her study-related records without violating the confidentiality of the subject. All information obtained in relation to this protocol regarding participating patients is protected according to Danish law on Patients Legal Rights and Data Protection.

12.5 Investigator Signatory Obligations:

Each clinical study report should be signed by the investigator or the coordinating investigator. The coordinating investigator meets at least one of the following criteria:

- A recognized expert in the therapeutic area
- An investigator, who provided significant contributions to either the design or interpretation of the Study.

13. ADMINISTRATIVE AND LEGAL OBLIGATIONS:

13.1 Protocol Amendments and Study Termination:

Protocol amendments must be made only with the prior approval of Steering Committee. Agreement from the investigator must be obtained for all protocol amendments and amendments to the informed consent document. The Ethics Committee must be informed of all amendments and give approval for any amendments likely to affect the safety of the subjects or the conduct of the trial. The Steering Committee reserve the right to terminate the study. The investigators reserve the right to withdraw from the study.

13.2 Study Documentation and Storage:

The investigator should maintain a list of appropriately qualified persons to whom he/she has delegated trial duties. All persons authorized to make entries and/or corrections on case report forms will be included on the sponsor Delegation of Authority Form. Source documents are original documents, data, and records from which the subject's case report form data are obtained. These include but are not limited to hospital records, clinical and office charts, laboratory and pharmacy records, diaries, microfiches, radiographs, and correspondence. Case report form entries may be considered source data if the case report form is the site of the original recording (i.e., there is no other written or electronic record of data). In this study, patient reported outcome information can be recorded directly on the web based CRF. It will be possible to perform electronic signature in the web based CRFs.

The investigator and study staff are responsible for maintaining a comprehensive and centralized filing system of all study-related (essential) documentation, suitable for inspection at any time by representatives of the sponsor and/or applicable regulatory authorities. Elements should include:

- Subject files containing completed case report forms, informed consents, and supporting copies of source documentation

• Study files containing the protocol with all amendments, investigator's brochure, copies of prestudy documentation (see section 12.3), and all correspondence to and from the EC
In addition, all original source documents supporting entries in the case report forms must be maintained and be readily available.
No study document should be destroyed without prior written agreement between the Steering Committee (or its appointed person) sponsor and the investigator.

13.3 Study Monitoring and Data Collection:

The Steering Committee representative and regulatory authority inspectors are responsible for contacting and visiting the investigator for the purpose of inspecting the facilities and, upon request, inspecting the various records of the trial (e.g., case report forms and other pertinent data) provided that subject confidentiality is respected. The monitor appointed by the Steering Committee is responsible for inspecting the case report forms at regular intervals as specified in the study monitoring plan, throughout the study to verify adherence to the protocol; completeness, accuracy, and consistency of the data; and adherence to local regulations on the conduct of clinical research. The monitor should have access to subject medical records and other study-related records needed to verify the entries on the case report forms.
The study is completed in accordance with this protocol and ICH-GCP guidelines. The investigator agrees to cooperate with the monitor to ensure that any problems detected in the course of these monitoring visits, including delays in completing case report forms, are resolved.
Inspection of site facilities and review of study-related records may occur to evaluate the trial conduct and compliance with the protocol, ICH-GCP, and applicable regulatory requirements.
All data should be entered into the web based CRF. The web based CRF should be completed when logged on to the system in the investigators own name.

• Corrections in the electronic CRF are made electronically. The change must be initialised and dated by the investigator or a member of the study staff authorized by the investigator. Corrections to electronic forms will be automatically documented through the software's "audit trail".
• To ensure the quality of clinical data across all subjects and sites, a clinical data management review will be performed on subject data received at the study office. During this review, subject data will be checked for consistency, omissions, and any apparent discrepancies. In addition, the data will be

reviewed for adherence to the protocol and GCP. To resolve any questions arising from the clinical
data management review process, data queries and/or site notifications will be sent to the site for completion and return to the study office.

• The principal investigator will sign and date the indicated places on the case report form. These signatures will indicate that the principal investigator inspected or reviewed the data on the case report form, the data queries, and the site notifications, and agrees with the content.

• The study office will correct the database for the following CRF issues **without notification** to site
staff:

- Misspellings, that do not change the meaning of the word (excluding adverse events and medications)
- Location of data recorded on an incorrect CRF (e.g., moving lab data from general comments to the
appropriate lab table)
- Date errors that occur into the new year
- Standard time to 24-hour clock
- Weight unit errors if a baseline weight has been established
- Height unit errors
- Administrative data (eg, event names for unscheduled visits or retests)
- Clarifying “other, specify” if data are provided (eg, race, physical exam)
- If both the end date and a status of continuing is indicated (eg, for adverse events, concomitant medication, hospitalization) the end date will supersede
- Deletion of obvious duplicate data (eg, same results sent twice with the same date but different clinical planned events – week 4 and early termination)
- For adverse events that record action taken code = 01 (none) and any other action code, 01 (none)
may be deleted as it is superseded by other existing data
- If equivalent units or terms are recorded instead of the acceptable the sponsor standard (e.g., cc for
mL, SQ for SC route, Not Examined for Not Done), the sponsor units or terms will be used
- If the answer to a YES or NO question is blank or obviously incorrect (e.g., Answers to the following
questions do not reflect the data that are recorded or missing: Were there any adverse events? Concomitant medications? Hospitalizations?)

All data will be kept for 15 years.

13.4 Language:

Case report forms will be in English. Registered trade names for concomitant medications may be used instead of generic names. All written information and other material to be used by subjects and investigative staff must use vocabulary and language that are clearly understood.

13.5 Publication Policy:

To coordinate dissemination of data from this study, a publication committee consisting of the steering committee and selected participants are formed. The committee is expected to solicit input and assistance from other investigators as appropriate. Membership of the committee does not guarantee authorship—the criteria described below should be met for every publication. Authorship of any publications resulting from this study will be determined on the basis of the Uniform Requirement for Manuscripts Submitted to Biomedical Journals (International Committee of Medical Journal Editors, 1997) which states:

Each author should have participated sufficiently in the work to take public responsibility for the content. Authorship credit should be based on only substantial contributions to:

(a) Conception and design, or analysis and interpretation of data.

(b) Drafting the article or revising it critically for important intellectual content.

(c) Final approval of the version to be published.

Conditions (a), (b), and (c) must all be met.

All publications (e.g., manuscripts, abstracts, oral/slide presentations, book chapters) based on this study must be submitted to the Steering Committee for review.

The results from the study - both positive and negative as well as inconclusive results– will be submitted for publication in international peer-reviewed scientific journals.

13.6 Compensation:

Subjects will be covered by normal patient insurance.

14. REFERENCES:

1. Koch MB, Davidsen M, Juel K.
   Heart Diseases in Denmark: Incidence and future development. Report from the Danish Heart Foundation. October 2011.
   http://www.hjerteforeningen.dk/files/Rapporter_mm/Hjertekarsygdomme%20i%20Danmark.% 20Forekomst%20og%20udvikling%202000-2009.pdf

2. Kirchhof P, Adamou A, Knight E, et al.
   How can we avoid a stroke crisis? ESC Working group report. Stroke prevention in atrial fibrillation. December 2009.
   http://www.escardio.org/communities/EHRA/publications/papers-interest/Documents/ehra-stroke-report-recommend-document.pdf

3. Camm AJ, Kirchhof P, Lip GY et al.
   Guidelines for the management of atrial fibrillation: The Task Force for the management of atrial fibrillation of the European Society of Cardiology.
   Europace. 2010; 12: 1360-1420.

4. Healey JS, Connolly SJ, Gold MR et al.
   Subclinical atrial fibrillation and the risk of stroke.
   NEJM 2012; 366: 120-9.

5. Savelieva I, Kakouros N, Kourliouros A, and Camm AJ.
   Upstream therapies for management of atrial fibrillation: review of clinical evidence and implications for European Society of Cardiology guidelines. Part I: primary prevention.
   Europace 2011; 13: 308-328.

6. De Jong AM, Maass AH, Oberdorf-Maass SU, Van Veldhuisen DJ, Van Gilst WH, Van Gelder IC. Mechanisms of atrial structural changes caused by stretch occurring before and during early atrial fibrillation.
   Cardiovasc Res 2010; 89: 754-765.

7. Nattel S, Burstein B and Dobrev D.
   Atrial Remodeling and Atrial Fibrillation: Mechanisms and Implications.
   Circ Arrhythmia Electrophysiol 2008; 1: 62-73.

8. Cheng S, Keyes MJ, Larson MG et al.
   Long-term outcomes in individuals with prolonged PR interval or first-degree atrioventricular block. JAMA 2009; 301: 2571-7.

9. Kolb C, Nürnberger S, Ndrepepa G et al.
   Modes of initiation of paroxysmal atrial fibrillation from analysis of spontaneously occurring episodes using a 12-lead Holter monitoring system.
   Am J Cardiol 2001; 88: 853-7.

10. Porsdal V, Boysen G.
    Direct costs during the first year after intracerebral hemorrhage.
    Eur J Neurol 1999; 6: 449-54.

11. Jørgensen HS, Nakayama H, Raaschou HO et al.
    Acute stroke care and rehabilitation: an analysis of the direct cost and its clinical and social

determinants. The Copenhagen Stroke Study.
Stroke 1997; 28: 1138-41.

12.Lunde L.
Can EQ-5D and 15D be used interchangeably in economic evaluations? Assessing quality of life in post-stroke patients.
Eur J Health Econ 2012 Jun 8. Epub ahead of print.

13.Larsen T, Olsen TS, Sorensen J.
Early home-supported discharge of stroke patients: A health technology assessment.
Int J Technol Assess Health Care 2006; 22: 313-20.

14.Pink J, Lane S, Pirmohamed M, Hughes DA.
Dabigatran etexialte versus warfarin in management of non-valvular atrial fibrillation in UK context: quantitative benefit-harm and economic analyses.
BMJ 2011 Oct 31;343:d6333.

15.Sorensen SV, Kansal AR, Connolly S et al.
Cost-effectiveness of dabigatran etexilate for the prevention of stroke and systemic embolism in atrial fibrillation: a Canadian payer perspective.
Thromb Haemost 2011; 105: 908-19.

16.You JH, Tsui KK, Wong RS, Cheng G.
Cost-Effectiveness of Dabigatran versus Genotype-Guided Management of Warfarin Therapy for Stroke Prevention in Patients with Atrial Fibrillation.
PLoS One 2012;7:e39640.

17.The CONSORT Group. http://www.consort-statement.org

18.Truelsen T, Piechowski-Jóźwiak B, Bonita R, Mathers C, Bogousslavsky J, Boysen G.
Stroke incidence and prevalence in Europe: a review of available data.
Eur J Neurol. 2006; 13: 581-98.

19.Knopman DS, Pennman AD, Catellier DJ, Coker LH et al.
Vascular risk factors and longitudinal changes on brain MRI - The ARIC study.
Neurology 2011; 76: 1879–1885,

20.Rostrup E, Gouw AA, Vrenken H, van Straaten ECW, Ropele S, Pantoni L, Inzitari D, Barkhof F, Waldemar G on behalf of the LADIS study group.
The spatial distribution of age-related white matter changes as a function of vascular risk factors— Results from the LADIS study.
NeuroImage 2012; 60: 1597-1607.

21.Pedersen KM.
DRG again again.
Ugeskr Laeger 2010; 172:2205.

22.Wittrup-Jensen KU, Lauridsen J, Gudex C et al.
Generation of a Danish TTO value set for EQ-5D health states.
Scand J Public Health 2009; 37: 459-66.

Supplementary references on the scientific themes:

Ahlsson A, Fengsrud E, Bodin L, Englund A.
Postoperative atrial fibrillation in patients undergoing aortocoronary bypass surgery carries an eightfold risk of future atrial fibrillation and a doubled cardiovascular mortality.
Eur J Cardiothorac Surg (2010), doi:10.1016/j.ejcts.2009.12.033

Botto GL, Padeletti L, Santini M, et al.
Presence and Duration of Atrial Fibrillation Detected by Continuous Monitoring: Crucial Implications for the Risk of Thromboembolic Events.
J Cardiovasc Electrophysiol, Vol. pp. 1-8.

Connolly SJ, Ezekowitz MD, Yusuf S et al.
Dabigatran versus Warfarin in Patients with Atrial Fibrillation.
N Engl J Med 2009;361:1139-51.

Hanke T, Charitos EI, Stierle U, et al.
Twenty-Four–Hour Holter Monitor Follow-Up Does Not Provide Accurate Heart Rhythm Status After Surgical Atrial Fibrillation Ablation Therapy. Up to 12 Months Experience With a Novel Permanently Implantable Heart Rhythm Monitor Device.
Circulation 2009;120[suppl 1]:S177–S184.

Huikuri H, Raatikainen MJP, Moerch-Joergens R, et al.
Prediction of fatal or near-fatal cardiac arrhythmia events in patients with depressed left ventricular function after an acute myocardial infarction.
European Heart Journal (2009) 30, 689–698. doi:10.1093/eurheartj/ehn537

Pedersen OD, Bagger H, Keller N, et al.
Efficacy of Dofetilide in the Treatment of Atrial Fibrillation-Flutter in Patients With Reduced Left Ventricular Function A Danish Investigations of Arrhythmia and Mortality ON Dofetilide (DIAMOND) Substudy.
Circulation 2001;104;292-296.

Sinha AM, Diener HC, Morillo CA, et al.
Cryptogenic Stroke and underlying Atrial Fibrillation (CRYSTAL AF): Design and rationale.
Am Heart J 2010;160:36-41.e1.

Pokushalov E, Romanov A, Corbucci G, et al.
Use of An Implantable Monitor to Detect Arrhythmia Recurrences and Select Patients for Early Repeat Catheter Ablation for Atrial Fibrillation: A Pilot Study.
Circ Arrhythm Electrophysiol. 2011 Dec;4(6):823-31. doi: 10.1161/CIRCEP.111.964809. Epub 2011 Sep 19.

Lamas G.
How much atrial fibrillation is too much atrial fibrillation?
N Engl J Med 2012 Jan 12;366(2):178-80. doi: 10.1056/NEJMe1111948.

Bloch Thomsen PE, Jons C, Raatikainen MJ, Moerch Joergensen R, et al.
Long-term recording of cardiac arrhythmias with an implantable cardiac monitor in patients with reduced ejection fraction after acute myocardial infarction: the Cardiac Arrhythmias and Risk Stratification After Acute Myocardial Infarction (CARISMA) study.
Circulation. 2010 Sep 28;122(13):1258-64. doi: 10.1161/CIRCULATIONAHA.109.902148. Epub 2010 Sep 13.

Buxton AE.
Implantable loop recorder in survivors of acute myocardial infarction: a glimpse of reality?
Circulation. 2010 Sep 28;122(13):1255-7. doi: 10.1161/CIRCULATIONAHA.110.976365. Epub 2010 Sep 13.

Heeringa J, van der Kuip DA, Hofman A, et al.
Prevalence, incidence and lifetime risk of atrial fibrillation: the Rotterdam study.
Eur Heart J. 2006 Apr;27(8):949-53. Epub 2006 Mar 9.

Brignole M, Vardas P, Hoffman E, et al.
Indications for the use of diagnostic implantable and external ECG loop recorders.
Europace. 2009 May;11(5):671-87. doi: 10.1093/europace/eup097.

Pedersen OD, Bagger H, Køber L, Torp-Pedersen C; TRACE Study Group.
Impact of congestive heart failure and left ventricular systolic function on the prognostic significance of atrial fibrillation and atrial flutter following acute myocardial infarction.
Int J Cardiol. 2005 Apr 8;100(1):65-71.

van Dam PM, van Oosterom A.
Analysing the potential of Reveal for monitoring cardiac potentials.
Europace. 2007 Nov;9 Suppl 6:vi119-23.

Pokushalov E, Taborsky M, Hindricks G., et al.
Variables Influencing Sensing after Reveal ImplanTation (VISIT).
Abstract presented at EURPACE 2009, 21 June - 24 June 2009, Berlin, Germany.

Friberg L, Hammar N, Pettersson H, Rosenqvist M.
Increased mortality in paroxysmal atrial fibrillation: report from the Stockholm Cohort-Study of Atrial Fibrillation (SCAF).
Eur Heart J. 2007 Oct;28(19):2346-53. Epub 2007 Aug 1.

Heeringa J, Conway DS, van der Kuip DA.
A longitudinal population-based study of prothrombotic factors in elderly subjects with atrial fibrillation: the Rotterdam Study 1990-1999.
J Thromb Haemost. 2006 Sep;4(9):1944-9. Epub 2006 Jul 5.

Petkar S, Hamid T, Iddon P.
Prolonged implantable electrocardiographic monitoring indicates a high rate of misdiagnosis of epilepsy--REVISE study.
Europace. 2012 Nov;14(11):1653-60. doi: 10.1093/europace/eus185. Epub 2012 Jun 28.

Zimetbaum P, Goldman A.
Ambulatory arrhythmia monitoring: choosing the right device.
Circulation. 2010 Oct 19;122(16):1629-36. doi: 10.1161/CIRCULATIONAHA.109.925610

Binici Z, Intzilakis T, Nielsen OW.
Excessive supraventricular ectopic activity and increased risk of atrial fibrillation and stroke.
Circulation. 2010 May 4;121(17):1904-11. doi: 10.1161/CIRCULATIONAHA.109.874982. Epub 2010 Apr 19.

Ravensbergen et al Regarding Article, “Long-Term Recording of Cardiac Arrhythmias

With an Implantable Cardiac Monitor in Patients With Reduced Ejection Fraction After Acute Myocardial Infarction: The Cardiac Arrhythmias and Risk Stratification After Acute Myocardial Infarction (CARISMA) Study" (Letter).
Circulation. 2012 Jan 3;125(1):e239

Friberg L, Hammar N, Rosenqvist M.
Stroke in paroxysmal atrial fibrillation: report from the Stockholm Cohort of Atrial Fibrillation.
Eur Heart J. 2010 Apr;31(8):967-75. doi: 10.1093/eurheartj/ehn599. Epub 2009 Jan 27.

Henriksson KM, Farahmand B, Asberg S, et al.
First-ever atrial fibrillation documented after hemorrhagic or ischemic stroke: the role of the CHADS(2) score at the time of stroke.
Clin Cardiol. 2011 May;34(5):309-16. doi: 10.1002/clc.20869. Epub 2011 Mar 13.

Kolominsky-Rabas PL, Heuschmann PU, Marschall D, et al.
Lifetime cost of ischemic stroke in Germany: results and national projections from a population-based stroke registry: the Erlangen Stroke Project.
Stroke 2006 May;37(5):1179-83. Epub 2006 Mar 30.

Edvardsson N, Frykman V, van Mechelen R, et al.
Use of an implantable loop recorder to increase the diagnostic yield in unexplained syncope: results from the PICTURE registry.
Europace. 2011 Feb;13(2):262-9. doi: 10.1093/europace/euq418. Epub 2010 Nov 19

Winkel TA, Rouwet EV, van Kuijk JP, et al.
Aortic surgery complications evaluated by an implanted continuous electrocardiography device: a case report.
Eur J Vasc Endovasc Surg. 2011 Mar;41(3):334-6. doi: 10.1016/j.ejvs.2010.11.006. Epub 2010 Dec 31.

Pokushalov E, Romanov A, Corbucci G, et al.
Ablation of paroxysmal and persistent atrial fibrillation: 1-year follow-up through continuous subcutaneous monitoring.
J Cardiovasc Electrophysiol. 2011 Apr;22(4):369-75. doi: 10.1111/j.1540-8167.2010.01923.x. Epub 2010 Oct 11.

Jons C, Jacobsen UG, Joergensen RM, et al.
The incidence and prognostic significance of new-onset atrial fibrillation in patients with acute myocardial infarction and left ventricular systolic dysfunction: a CARISMA substudy.
Heart Rhythm. 2011 Mar;8(3):342-8. doi: 10.1016/j.hrthm.2010.09.090. Epub 2010 Nov 18

Goetze JP, Friis-Hansen L, Rehfeld JF.
Atrial secretion of B-type natriuretic peptide.
Eur Heart J. 2006 Jul;27(14):1648-50. Epub 2006 Jun 19.

Hindricks G, Pokushalov E, Urban L, et al.
Performance of a new leadless implantable cardiac monitor in detecting and quantifying atrial fibrillation: Results of the XPECT trial.
Circ Arrhythm Electrophysiol. 2010 Apr;3(2):141-7. doi: 10.1161/CIRCEP.109.877852. Epub 2010 Feb 16.

El-Chami MF, Kilgo P, Thourani V, et al.

New-onset atrial fibrillation predicts long-term mortality after coronary artery bypass graft.
J Am Coll Cardiol. 2010 Mar 30;55(13):1370-6. doi: 10.1016/j.jacc.2009.10.058.

Pokushalov E, Romanov A, Cherniavsky A, et al.
Ablation of paroxysmal atrial fibrillation during coronary artery bypass grafting: 12 months' follow-up through implantable loop recorder.
Eur J Cardiothorac Surg. 2011 Aug;40(2):405-11. doi: 10.1016/j.ejcts.2010.11.083. Epub 2011 May 23.

Kirchhof P, Lip GY, Van Gelder IC, et al.
Comprehensive risk reduction in patients with atrial fibrillation: emerging diagnostic and therapeutic options—a report from the 3rd Atrial Fibrillation Competence NETwork/European Heart Rhythm Association consensus conference.
Europace. 2012;14(1):8-27

Fang MC, Go AS, Chang Y, et al.
Comparison of risk stratification schemes to predict thromboembolism in people with non-valvular atrial fibrillation.
J Am Coll Cardiol 2008;51(8):810-815

Camm AJ, Corbucci G, Padeletti L.
Usefulness of Continuous Electrocardiographic Monitoring for Atrial Fibrillation.
Am J Cardiol 2012; 110(2):270-276

Glotzer TV, Hellkamp AS, Zimmerman J, et al.
Atrial high rate episodes detected by pacemaker diagnostics predicts death and stroke.
Circulation 2003;107(12):1614-1619

Capucci A, Santini M, Padeletti L, et al.
Monitored atrial fibrillation duration predicts arterial embolic events in patients suffering from bradycardia and atrial fibrillation implanted with antitachycardia pacemakers.
J Am Coll Cardiol 2005; 46(10):1913–1920

Glotzer TV, Daoud EG, Wyse DG, et al.
The Relationship between Daily Atrial Tachyarrhythmia Burden From Implantable Device Diagnostics and Stroke Risk. The TRENDS Study.
Circ Arrhythmia Electrophysiol 2009;2(5):474-480

Boriani G, Botto GL, Padeletti L, et al.
Improving stroke risk stratification using the CHADS2 and CHA2DS2VASc risk scores in paroxysmal atrial fibrillation patients by continuous arrhythmia burden monitoring.
Stroke 2011;42(6):1768-1770

Santini M, Gasparini M, Landolina M, et al.
Device-detected atrial tachyarrhythmias predict adverse outcome in real-world patients with implantable biventricular defibrillators.
J Am Coll Cardiol 2011. 57(2):167-172

Welles CC, Whooley MA, Na B, Ganz P, Schiller NB, Turakhia MP.
The CHADS2 score predicts ischemic stroke in the absence of atrial fibrillation among subjects with coronary heart disease: Data from the Heart and Soul Study,
Am Heart J. 2011;162(3):555-561

Purerfellner H, Gillis AM, Holbrook R, Hettrick DA.
Accuracy of atrial tachyarrhythmia detection in implantable devices with arrhythmia therapies.
Pacing Clin Electrophysiol. 2004; 27(7): 983–992

Lamas G.
How Much Atrial Fibrillation Is Too Much Atrial Fibrillation?
N Engl J Med 2012; 366(2):178-180

Boriani G., Santini M., Lunati M, .et al.
Improving Thromboprophylaxis Using Atrial Fibrillation Diagnostic Capabilities in Implantable Cardioverter-Defibrillators: The Multicentre Italian ANGELS of AF Project,
Circ Cardiovasc Qual Outcomes 2012; 5(2):182-188

van Elderen SG, de Roos A, de Craen AJ, et al.
Progression of brain atrophy and cognitive decline in diabetes mellitus: a 3-year follow-up.
Neurology. 2010 Sep 14;75(11):997-1002. doi: 10.1212/WNL.0b013e3181f25f06.

Knopman DS, Penman AD, Catellier DJ, et al.
Vascular risk factors and longitudinal changes on brain MRI: the ARIC study.
Neurology. 2011 May 31;76(22):1879-85. doi: 10.1212/WNL.0b013e31821d753f. Epub 2011 May 4.

Hajjar I, Quach L, Yang F, et al.
Hypertension, white matter hyperintensities, and concurrent impairments in mobility, cognition, and mood: the Cardiovascular Health Study.
Circulation. 2011 Mar 1;123(8):858-65. doi: 10.1161/CIRCULATIONAHA.110.978114. Epub 2011 Feb 14.

Portet F, Brickman AM, Stern Y, et al.
Metabolic syndrome and localization of white matter hyperintensities in the elderly population.
Alzheimers Dement. 2012 Oct;8(5 Suppl):S88-95.e1. doi: 10.1016/j.jalz.2011.11.007. Epub 2012 Jun 6.

Raz N, Yang Y, Dahle CL, et al.
Volume of white matter hyperintensities in healthy adults: contribution of age, vascular risk factors, and inflammation-related genetic variants.
Biochim Biophys Acta. 2012 Mar;1822(3):361-9. doi: 10.1016/j.bbadis.2011.08.007. Epub 2011 Aug 25.

Rostrup E, Gouw AA, Vrenken H, et al.
The spatial distribution of age-related white matter changes as a function of vascular risk factors--results from the LADIS study.
Neuroimage. 2012 Apr 15;60(3):1597-607. doi: 10.1016/j.neuroimage.2012.01.106. Epub 2012 Jan 28.

Seo SW, Lee JM, Im K, et al.
Cardiovascular risk factors cause cortical thinning in cognitively impaired patients: relationships among cardiovascular risk factors, white matter hyperintensities, and cortical atrophy.
Alzheimer Dis Assoc Disord. 2012 Apr-Jun;26(2):106-12. doi: 10.1097/WAD.0b013e31822e0831.

Gorelick PB, Scuteri A, Black SE, et al.
Vascular contributions to cognitive impairment and dementia: a statement for healthcare professionals from the american heart association/american stroke association.
Stroke. 2011 Sep;42(9):2672-713. doi: 10.1161/STR.0b013e3182299496. Epub 2011 Jul 21.

Zhu YC, Chabriat H, Godin O, et al.
Distribution of white matter hyperintensity in cerebral hemorrhage and healthy aging.
J Neurol. 2012 Mar;259(3):530-6. doi: 10.1007/s00415-011-6218-3. Epub 2011 Aug 30.

15. APPENDICES:

Appendix A – Study procedures – summary

Appendix B – Composition of Committees

Appendix C – Serious Adverse Events

Appendix D – Blood Samples for Genetic analysis and Blood Samples for Biomarkers

Appendix E – ILR information

Appendix F – Definition of composite endpoint

Appendix G – Funding

Appendix H – Informed consent

Appendix I – Guidelines for oral information

Appendix A – Study procedures – Summary
Study flow chart – a) ILR group

Phase	Screen	Rand.	Impl.	Follow-up						
**Visit**	**1**	**2**	**3**	**3A**	**4**	**4A**	**5**	**5A**	**6**	**6A**
**Month**	0,5	0	0,5		12,5	13-24	24,5	25-36	36,5	37-48
**Day**	-14	0	14		374		734		1094	
**Informed consent**	√									
**Medical history**	√				√		√		√	
**Concomitant Disease**	√				√		√		√	
**Concomitant Medication**	√				√		√		√	
**Demographics**	√									
**Echocardiography**		√								
**12-lead ECG**	√				√		√		√	
**Randomisation**		√								
**Vital signs**	√				√		√		√	
**QOL and cognitive function**	√				√		√		√	
**Blood sample**	√									
**Adverse events**					√		√		√	
**MRI**		(√)		(√)		(√)				
**Health economics**					√		√		√	
**Remote ILR interrogation #**			√	√	√	√	√	√	√	√

# : only relevant for the ILR group; remote transmissions are sent automatically on a daily basis and routinely as patient activated every 4 weeks (to obtain ECG data during presumed sinus rhythm) during the entire study period

Appendix A – Study procedures – Summary
Study flow chart – b) Control group

Phase	Screen	Rand.	Impl.	Follow-up						
**Visit**	**1**	**2**	**3**	**3A**	**4**	**4A**	**5**	**5A**	**6**	**6A**
**Month**	0,5	0	0,5		12,5	13-24	24,5	25-36	36,5	37-48
**Day**	-14	0	14		374		734		1094	
**Informed consent**	√									
**Medical history**	√				√		√		√	
**Concomittant. Disease**	√				√		√		√	
**Concomittant Medication**	√				√		√		√	
**Demographics**	√									
**Echocardiography**		√								
**12-lead ECG**	√								√	
**Randomisation**		√								
**Vital signs**	√								√	
**QOL and cognitive function**	√				√		√		√	
**Blood sample**	√									
**Adverse events**					√		√		√	
**MRI**		(√)		(√)		(√)				
**Health economics**					√		√		√	
**Remote ILR interrogation #**										

# : only relevant for the ILR group; remote transmissions are sent automatically on a daily basis and routinely as patient activated every 4 weeks (to obtain ECG data during presumed sinus rhythm) during the entire study period

Appendix B – Composition of Committees

Composition of the Steering committee (SC):

The SC, which consists of representatives from the founding partner institutions, will provide advice as to the project´s overall scientific quality and budget as well as concerning the balance between the project´s activities, goals and results.

The members of the Steering committee are:
Jesper Hastrup Svendsen, Rigshospitalet (chairman)
Lars Køber, Rigshospitalet
Søren Højberg, Bispebjerg Hospital
Ketil Haugan, Roskilde Hospital
Axel Brandes, Odense University Hospital
Christian Kronborg, University of Southern Denmark
Claus Graff, Aalborg University
Derk W. Krieger, Bispebjerg Hospital

Composition of the Event Committee (EC):

The event committee is responsible for evaluating and adjudicating events comprising the primary endpoints and AF burden. The EC will classify deaths according to pre-specified criteria modified according to Hinkle and Thaler. Deaths will be classified as cardiovascular and non-cardiovascular. Cardiovascular will be divided into sudden and non-sudden cardiovascular deaths based on time specifications. Strokes will be divided into ischaemic and non-ischaemic strokes (bleeds) and non-classifiable strokes. The guidelines for classifications will be described in a separate charter.
There will be a neurologist and a cardiologist in the EC. The members of the EC are:

- Professor Derk W. Krieger and
- Cardiologist NN (TBD)

Composition of the International Advisory Committee (IAC):

An *International Scientific Advisory Group*, which will meet annually, will discuss issues of relevance for the project (theory, methods and results) and consist of internationally recognised experts in the field. The members of the IAC are:

- Professor Gregory Y.H. Lip, University of Birmingham, UK;
- Professor Mårten Rosenqvist, Karolinska Institute, Sweden; and
- Professor Dan Atar, University of Oslo, Norway.

They have all agreed to serve in the advisory group. All three are senior cardiologists with a strong scientific track record in atrial fibrillation.

Appendix C – Serious Adverse Events

Serious adverse events (SAE) are reported immediately in the electronic case report forms. Every 6 month a report is generated to the Steering Committee. This report contains information on all deaths and strokes and all implantation related adverse events. Since the study is performed in a study population with an increased risk of stroke and mortality SAE will not be reported on a daily basis. For information this report will also be forwarded to the Ethics committee of the Capitol Region of Denmark. This report will be accompanied by a description of the events together with a statement regarding eventual consequences for the study.

Appendix D – Blood Samples for Genetic analysis and Blood Samples for Biomarkers

On inclusion, blood samples will be taken for routine measurement of electrolytes (Na and K), creatinine, haemoglobin concentration, white blood cells, platelets, troponins, pro-BNP and CRP (a total of 15 ml venous blood).

In addition, a total of 32 ml of venous blood is collected and stored in a research biobank.

The sampled blood for the research biobank is collected into a total of 6 vials:

A part of the blood is collected in 3 x 6 ml vials (purple coloured, K2EDTA) and stored as full-blood (a part is stored in vials containing RNAlater).

In addition, one vial containing 6 ml blood (purple coloured, K2EDTA) and 2 vials containing 4 ml blood (Z serum clot activator) are collected for centrifuging and after pipetting the supernatant is stored as plasma and serum, respectively.

Centrifuging is performed within 30 minutes after venous puncture and carried out at 3,000 RPM for 10 minutes. Samples are stored in freezer at minus 80 degrees Celcius.

Samples will be transferred to Rigshospitalet where they will be stored in the study related research biobank in a locked freezer. Surplus biological material from the research biobank is stored in a biobank for future research.

Appendix E – Device information: the Medtronic Reveal® LINQ:

In this study the Medtronic Reveal® LINQ insertable, cardiac monitor is used. The Reveal® LINQ is the newest and smallest ILR on the market which is expected to be market released by February 2014. It has battery longevity of three years and additionally 12 months of shelf life. Its size is 1.19 cc which is about 10% of the previous model (Medtronic Reveal® LINQ which has a volume of 9 cc). In contrast to the XT model the LINQ model has a build-in antenna which enables automatic remote transmissions. The device has an event memory of a total of 57 minutes (which is longer than the memory of the XT model: 49.5 minutes) (128 Hz) with up to 30 events in total. The total memory capacity is divided into 22.5 minutes of patient activated events (with the three most recent manual events lasting 7.5 minutes each), and additionally 27 minutes auto-activated events. The device is magnetic resonance (MR) conditional, which means that the Reveal® is safe for use in MR environments. Due to the algorithm the device uses for AF detection it will only categorize an arrhythmia event as atrial fibrillation, if it lasts more than two minutes in total. The arrhythmia detection capabilities of the Reveal® LINQ categorize arrhythmias into 5 groups: Fast VT, VT, bradycardia, asystolia, and AT/AF. The Reveal® LINQ Recorder is prepared for CareLink remote monitoring, with both automated daily transmissions and patient activated transmissions. The device can record AT/AF burden, heart rate variability, day versus night frequency and patient activity. Prior to implant it is recommended to check the vector of the device, with the purpose of finding the orientation with optimal R wave amplitude via the programmer. The device is supplied with a so-called patient assistant, which is a patient activating device. The device algorithm for atrial fibrillation detection is based on measurement of the RR-intervals and the delta RR-interval (change in RR interval from the preceding RR interval), which is afterwards visually presented in a so-called Lorenz plot (cluster signature matrix), where normal sinus rhythm or atrial flutter will be shown as a highly concentrated black spot in the center of the plot, whereas atrial fibrillation will be widely distributed in the cluster signature matrix (”gunshot profile”).

When patient activated episodes are stored, there is a total of 22.5 minutes for the episodes, and the device will always store the three last events, each lasting 7.5 minutes. Each of these 7.5 minute events will comprise 6.5 minutes prior to patient activation as well as 1 minute after patient activation. After patient activation a new episode will only be stored, if it happens more than 5 minutes after last activation. If the memory is full, the oldest episode will be overwritten. Per episode type there is a guaranteed storage of the three most resent episodes. When memory is full, the oldest stored ECGs will be removed. If data have been transmitted in the form of ECGs these will be stored forever as ECGs on Carelink. All episodes recorded by the loop recorder will be stored (as episodes with interpretation markers, but not necessarily as ECGs).

The system offers a visual indication to provide the patient with information that either everything is fine (no arrhythmia has been detected) (green lamp) or something with the heart rhythm needs action – please contact hospital (red lamp).

In the LOOP study it may seem reasonable to turn off one of the VT zones to increase the data storage.

In patients with frequent premature supraventricular contractions which the device incorrectly classify as atrial fibrillation, the device can be programmed to ignore these episodes by programming the function “ectopy rejection” “on” which will cause these episodes to be categorised as sinus rhythm.

It is possible to bring the Carelink box to another country and make transmissions from abroad.

Suggested device programming of parameters:

a) FVT parameters are set at: 30/40 and a rate >222 bpm
b) VT parameters are set at: 24 consequtive beats at a rate > 182 bpm
c) Bradycardia is set at a rate < 30 bpm
d) Asystolia is set at a duration > 4.5 sec
e) Symptom activation is programmed on and programmed to 3 episodes of 7.5 min each
f) AT/AF is programmed at AF only on
g) Ectopy rejection is programmed off in standard settings (only programmed on in case it is observed that the device falsely categorises premature contractions as AF.

Since the LINQ Reveal has not yet been market released we do not fully know the ideal programming yet.

When the ILR reaches end of battery life the device is explanted. If a patient dies with an implanted ILR the device is explanted by a post-mortem procedure.

Appendix F – Definition of composite endpoint

Brain imaging is required for all patients with symptoms of stroke or TIA to distinguish hemorrhagic from ischemic stroke.

1. Ischemic stroke is defined as the sudden onset of a focal neurologic deficit in a location consistent with the territory of a major cerebral artery and categorized as ischemic, hemorrhagic, or unspecified (in the case of patients who did not undergo brain imaging or in whom an autopsy was not performed).

2. Intracranial hemorrhage consists of hemorrhagic stroke (cerebral hemorrhage) and subdural or subarachnoid hemorrhage.

Explanation: Above definitions have been used in the RELY, ARISTOTELES and ROCKET AF studies.

3. Transient ischemic attack (TIA) is defined as a transient episode of neurologic dysfunction caused by focal brain, spinal cord, or retinal ischemia, **without** acute infarction.

Explanation: TIA was originally defined clinically by the temporary nature (<24 hours) of the associated neurologic symptoms. However, the arbitrary nature of the 24-hour time limit and lack of specific pathophysiologic meaning hampered the clinical and research utility of the term "TIA." Recognition of these problems led to a change to a tissue-based definition of TIA. The change was driven by advances in neuroimaging that enabled very early identification of ischemic brain injury and endorsed by 2009 guidelines from the American Heart Association and American Stroke Association (AHA/ASA).

Appendix G –Funding

Research grants have been obtained from the Danish National Foundation for Strategic Research (DKK 15.6 mio), the Research Foundation for the Capital Region of Denmark (DKK 2.0 mio) and the Danish Heart Foundation (DKK 150.000).
The first 600 loop recorders used in the project will be purchased from the study funding from the Danish National Foundation for Strategic Research. The condition of this purchase has been altered so that the Reveal LINQ´s will be offered on the same terms as the original Reveal XT devices. The additional 900 Reveal LINQ loop recorders will be donated from Medtronic as an unrestricted research grant (which has a value of DKK 5.4 mio (reduced price) or DKK 22.5 mio (ordinary list price)) and a grant to cover salary for research nurses at the hospitals (DKK 1.9 mio). Medtronic has decided to support the LOOP study with an unrestricted research grant covering salaries for one research nurse for one year at each of the four participating hospitals (i.e. a total of four years of salary for research nurses; estimated salary per nurse including pension and vacation is 40,000 DKK per month which equals 480,000 DKK per year/nurse; i.e. a total of 1.920.000 DKK (the same salary estimate was used in the application to the Strategic Research Council).
Additional research grants will be applied for from private and public research foundations as well as from industry. Study participants and the Ethics committee will be informed about additional funding.
Patients will not receive compensation for their participation in the study, but may in selected cases receive compensation for their transport expenses.

Appendix H – Informed consent

Appendix I – Guidelines for oral information

Retningslinjer for mundtlig deltagerinformation.
Informationen gives af en person, som har de sundhedsfaglige forudsætninger for at kunne informere om forskningsprojektet og som har direkte tilknytning til dette, hvilket i praksis betyder at informationen gives af læge eller forskningssygeplejerske på et af de hospitaler der deltager i undersøgelsen.

Forsøgspersonerne er identificeret ved:

- Et registertræk hvor man har fundet personer der opfylder in- og eksklusionskriterier
- Personer der løbende identificeres på de deltagende hospitaler (i forbindelse med indlæggelse eller ambulant kontrol) og som opfylder in- og eksklusionskriterier
- Personer som henvender sig til hospitalerne fordi de ønsker at deltage og som opfylder in- og eksklusionskriterier (disse vil potentielt kunne komme fra andre end de 3 deltagende regioner)

De potentielle forsøgspersoner vil få følgende initiale skriftlige information:

LOOP forsøget

Titel: Sammenhængen mellem blodprop i hjernen og atrieflimren påvist ved langvarig registrering af hjerterytmen med loop recorder.

Med dette brev vil vi spørge, om du vil deltage i en forskningsundersøgelse. Forsøget har til formål at afklare, om man kan forebygge blodprop i hjernen ved at overvåge hjerterytmen med et lille apparat (en såkaldt loop recorder), der placeres under huden på forsiden af brystkassen. Nogle af de personer der deltager vil få placeret det lille apparat, mens andre ikke vil få dette (dette afgøres ved en lodtrækning). I forskningsundersøgelsen deltager i alt 6.000 patienter og af disse vil hver fjerde (dvs. 1.500) efter lodtrækning få placeret det lille apparat.

Vi har fundet frem til dig ved at søge i registre efter personer med adresse i Region Hovedstaden, Region Sjælland eller Region Syddanmark, der er fyldt 70 år og desuden har en eller flere af sygdommene: Forhøjet blodtryk, sukkersyge, hjertesvigt eller en tidligere blodprop i hjernen ("slagtilfælde"). Du kan også være blevet fundet i forbindelse med en nylig hospitalskontakt, eller ved at du har kontaktet os, fordi du har hørt om forsøget. Nogen gange kan der være fejl i

registerdiagnoser, så hvis du fejlagtigt er blevet registreret med en af de nævnte diagnoser, beder vi dig orientere os herom.

Såfremt du allerede har fået påvist den hjerterytmeforstyrrelse, der kaldes atrieflimren kan du desværre ikke deltage. Du kan heller ikke deltage i forsøget, hvis du behandles med blodfortyndende medicin af typen Marevan, Pradaxa, Eliquis eller Xarelto

Hvis du er interesseret i at høre mere om forsøget kan du **kontakte projektsygeplejerske Sisse Lund (telefonnummer 35450686 eller e-mail: sisse.maj.lund.jensen@regionh.dk),** som vil sikre, at du kan deltage i forsøget.

Såfremt du ønsker at deltage, vil det være en god ide, hvis du kan have et familiemedlem eller en god ven med til samtalen på sygehuset.

Med venlig hilsen

Jesper Hastrup Svendsen
Professor, dr.med.

Den detaljerede mundtlige patientinformation gives til forsøgspersonen ved besøget på et af de deltagende centre, hvor selve randomiseringen foregår. Samtalen foregår i et uforstyrret rum. Forskningsprojektet vil blive fremlagt i et for forsøgspersonen forståeligt sprog, afpasset efter forsøgspersonens forudsætninger (alder, viden etc.). Det pointeres at det drejer sig om et biomedicinsk forskningsprojekt, samtalen indeholder oplysninger om eventuelle forudsigelige risici, bivirkninger, komplikationer og ulemper, samt at der kan være uforudsigelige risici og belastninger knyttet til deltagelse i et biomedicinsk forskningsprojekt. Ved samtalen udleveres skriftligt informationsmateriale, samt folderen ”Før du beslutter dig”, der omhandler patientrettigheder i forbindelse med biomedicinske forsøg i Danmark. Efter samtalen har forsøgspersonen 24 timers betænkningstid, evt. længere tid hvis dette er nødvendigt. Hvis forsøgspersonen er i stand til at træffe beslutning med det samme efter samtalen vil randomiseringen foregå samme dag, efter underskrift af informed concent.

Såfremt forsøgspersonen ønsker at deltage og bliver randomiseret, vil supplerende information efterfølgende blive givet til patienten:

- Såfremt der under gennemførelsen af forsøget fremkommer nye oplysninger om effekt, risici, bivirkninger, komplikationer eller ulemper
- Såfremt forskningsprojektets forsøgsdesign ændres væsentligt i forhold til forsøgspersonens sikkerhed
- Såfremt der under gennemførelsen af forskningsprojektet fremkommer væsentlige oplysninger om forsøgspersonens helbredstilstand, med mindre forsøgspersonen utvetydigt har givet udtryk for, at den pågældende ikke ønsker dette
- Ved projektets afrappportering informeres forsøgspersonen om de resultater, der er opnået samt om eventuelle konsekvenser for den enkelte. Dette forudsætter, at det er praktisk mulig og forsøgspersonen ønsker dette.

Final protocol

Atrial Fibrillation detected by continuous ECG monitoring using implantable loop recorder to prevent stroke in high-risk individuals -

The LOOP study

By the Steering Committee of the LOOP Study

Trial Sponsor:

The LOOP Study Steering Committee chairman Jesper Hastrup Svendsen
Department of Cardiology, section 2013
Rigshospitalet
Blegdamsvej 9, DK-2100 Copenhagen, Denmark

Protocol Version 63
Version date: 5th July 2019
Approved by the Steering Committee at meeting: August 16th 2019
Protocol number in the Ethics Committe of the Capital Region of Denmark: H-4-2013-025
ClinicalTrials Gov number: NCT02036450

Protocol synopsis

Title:

The LOOP study: Atrial Fibrillation detected by continuous ECG monitoring using implantable Loop recorder to prevent stroke in high-risk individuals.

Background:

Stroke is a major health problem, which affects approximately 16,000 people annually in Denmark, causing severe and burdensome consequences for its victims, who suffer a significant loss in quality of life (QoL), and their families. Furthermore, the healthcare costs due to stroke are sizeable, amounting to more than EUR 38 bn in the EU in 2006. Stroke is often caused by atrial fibrillation (AF), which is the most common type of cardiac arrhythmia. Effective management of AF with anticoagulation therapy is available and may have considerable benefits for patients as well as society. Difficult to detect, however, asymptomatic AF is usually only diagnosed by chance. Recently developed technology (implantable loop recorder; ILR) allows continuous long-term electrocardiography (ECG) monitoring and may constitute a substantial improvement in AF diagnosis. Furthermore, long-term ECG monitoring is essential for studying the significance of AF in stroke pathophysiology. The enormous socio-economic consequences of stroke mean that even a modest reduction in the number of strokes would benefit society.

Primary objective:

The primary objective of this study is to determine whether screening for AF with ILR and initiation of oral anticoagulation (OAC) if AF is detected, will reduce the risk of stroke and systemic arterial embolism in patients with risk factors for stroke.

Secondary objectives:

Secondary objectives include studies of whether the screening will reduce the risk of the combined endpoint of stroke, systemic arterial embolism, and mortality, health economic analyses, quality-of-life assessments, cognitive function assessments, and studies of AF characterization and risk markers from 12-lead ECG, genetics, cardiac and brain imaging, biochemistry, and more.

Study design:

Randomized, un-blinded, controlled parallel two group trial.

Endpoints:

Primary endpoint: Time to first stroke or systemic arterial embolism

Secondary endpoints: Time to adjudicated ischemic stroke/transient ischemic attack/systemic arterial embolism, time to adjudicated stroke, systemic arterial embolism, or cardiovascular death, time to adjudicated cardiovascular death, time to death by any cause

Other endpoints: time to diagnosis of AF, time to initiation of OAC, time to adjudicated intracranial hemorrhage not classified as stroke, time to adjudicated hemorrhagic stroke, time to major bleeding as defined by the International Society on Thrombosis and Hemostasis, complications related to loop recorder implantation, change from baseline in quality of life

Sample size:

In total 6,000 subjects will participate: 1,500 randomized to receive an ILR (ILR group) and 4,500 randomized to receive standard care (control group).

Summary of Subject Eligibility Criteria:

Subjects will be 70 years of age or older and will have at least one of the following diseases: hypertension, diabetes, heart failure, or previous stroke, while exclusion criteria include any history of AF or current contraindication to OAC.

ILR group:

Patients randomized to the ILR group will receive the monitoring device, and if AF is detected, OAC will be initiated according to study guidelines.

Control group:

Patients will be treated according to standard care.

Study Duration:

The study comprises an inclusion period of approximately 2 years followed by at intervention phase of minimum 3 years. The total study duration will therefore be approximately 5 years.

Screening and Randomization:

After randomization, patients allocated to the ILR group should receive the implantation as fast as possible, and preferably within 4 weeks.

Statistical Considerations:

We expect that 30% of the participants will have documented AF episodes during ILR monitoring for at least three years. The study population is expected to have a stroke rate of 0.7%/year in non-AF patients, and 2%/year in AF patients not treated with OAC. We assume that 3% of all participants in the control group will become diagnosed with AF and start OAC. We expect the number of AF-related strokes to be reduced by OAC. The annual rate of the primary end point in the control group is expected to be 1%. Finally, we expect that 5% of the ILR group will not be monitored due to refusal of ILR implantation or ILR explantation due to discomfort. We assume an overall hazard ratio of 0.65 for ILR vs control. To analyse this reduction in the primary endpoint with a 2-sided α-level of 0.05 and a power of 80%, we will need 279 primary events. Follow-up duration will be adjusted to accommodate the number of primary events.

Primary Endpoint Analysis:

The principal analysis for the primary endpoint will employ the intent-to-threat principle and use a survival analysis. To account for the competing risk of death, the cumulative incidences of the primary endpoint will estimated, plotted and group-wise compared in a multi-state fashion. A co-variate adjusted analysis of the combined primary endpoint using a cause-specific Cox proportional regression model will be performed as a supportive analysis. The hazard ratios and the corresponding 95% confidence intervals will be estimated. Subjects completing the study and not reaching the composite endpoint will be censored.

Secondary Endpoint Analysis:

All time-to-event secondary endpoints will be analysed similarly to the primary endpoint.

Event Committee:

An event adjudication committee will classify endpoint events throughout the study.

Funding:

Research grants have been obtained from the Danish National Foundation for Strategic Research (DKK 15.6 mio), the Research Foundation for the Capital Region of Denmark (DKK 2.0 mio) and the Danish Heart Foundation (DKK 150.000). MEDTRONIC Bakken Research Center B.V.(manufacturer of the ILRs will donate an unrestricted research grant to the study covering costs related to 900 ILRs (which has a value of DKK 5.4 mio (reduced price) or DKK 22.5 mio (ordinary list price)) and a grant to cover salary for research nurses at the hospitals (DKK 1.9 mio).

Additional research grants will be applied for from private and public research foundations as well as from industry.

Protocol Signatures:

Responsible for the clinical part of the study:

Professor Jesper Hastrup Svendsen
Department of Cardiology, The Heart Centre, Rigshospitalet
Blegdamsvej 9, 2100 Copenhagen
Phone no. +45 3545 2817; E-mail: Jesper.Hastrup.Svendsen@regionh.dk

Professor Lars Køber
Department of Cardiology, The Heart Centre, Rigshospitalet
Blegdamsvej 9, 2100 Copenhagen
Phone no. +45 3545 3519; E-mail: Lars.Koeber@regionh.dk

Consultant Søren Højberg
Department of Cardiology Y, Bispebjerg Hospital
Bispebjerg Bakke 23, 2400 Copenhagen NV
Phone no. +45 3531 2578; E-mail: soeren.hoejberg@regionh.dk

Consultant Ketil Haugan
Department of Cardiology, Roskilde Hospital
Køgevej 7-13, 4000 Roskilde
Phone no. +45 4732 6000; E-mail: khau@regionsjaelland.dk

Consultant Axel Brandes
Department of Cardiology, Odense University Hospital
Sdr. Boulevard 29, 5000 Odense C
Phone no. +45 6611 3333. E-mail: axel.brandes@ouh.regionsyddanmark.dk

Professor Derk Krieger,
Department of Neurology, Bispebjerg Hospital.
Bispebjerg Bakke 23, 2400 København NV

Responsible for monitoring:

Steering committee:
c/o Professor Jesper Hastrup Svendsen
Department of Cardiology, section 2013
Rigshospitalet, Blegdamsvej 9, 2100 Copenhagen, Denmark

Members of the Steering Committee of the LOOP Study:

Professor Jesper Hastrup Svendsen, Department of Cardiology, Rigshospitalet (chairman).
Professor Lars Køber, Department of Cardiology, Rigshospitalet.
Consultant, associate professor Axel Brandes, Department of Cardiology, Odense University Hospital.
Consultant Ketil Haugan, Department of Cardiology, Roskilde Hospital.
Consultant Søren Højberg, Department of Cardiology, Bispebjerg Hospital.
Professor Derk Krieger, Department of Neurology, Bispebjerg Hospital.
Associate Professor Christian Kronborg, University of Southern Denmark.
Associate Professor Claus Graff, Aalborg University.

Additional Researchers:

Resident Søren Zöga Diederichsen, Department of Cardiology, Rigshospitalet.

Study glossary:
AF: Atrial fibrillation
AE: Adverse event
CIED: Cardiac Implantable Electronic Device
EC: Endpoint Committee
eCRF: Electronic Case Report Form
ILR: Implantable Loop Recorder
QoL: Quality of Life
SAE: Serious adverse event

SC: Steering Committee

Stroke: rapid loss of brain function due to disturbed blood supply to the brain.

TIA: Transient Ischaemic Attack

Table of Contents

Protocol synopsis	2
Protocol signatures	5
Responsible for the study	5
Responsible for monitoring	5
1. OBJECTIVES	9
1.1 Primary	9
1.2 Secondary	9
1.3 Expected results	9
2. BACKGROUND and RATIONALE	10
2.1 Stroke and AF	10
2.2 ECG and AF risk	11
2.3 Health economy and QoL	11
2.4 Study Rationale	12
2.5 Clinical Hypotheses	12
3. EXPERIMENTAL PLAN	12
3.1 Study Design	13
3.2 Number of Centres	13
3.3 Number of Subjects	13
3.4 Recruitment	13
3.5 Estimated Study Duration	13
4. SUBJECT ELIGIBILITY	14
4.1 Inclusion Criteria	14
4.2 Exclusion Criteria	14
5. SUBJECT ENROLMENT	15
5.1 Recruitment and inclusion visit	15
5.2 Subject Registration	16
5.3 Screen Failures	16
5.4 Randomization and Treatment Assignment	16
5.5 Re-Screening	17
6. TREATMENT PROCEDURES	17
6.1 ILR device	17
6.2 ILR procedures	18
6.3 Concomitant Therapy	18
6.4 Prescribed therapy during the study period	18
7. STUDY PROCEDURES	19
7.1 Subject Screening	19
7.2 Follow-up Phase	19
7.3 Endpoint Determination	24
7.4 Genetic analysis and Biomarkers	24
7.5 Strategy for management of low inclusion rate	24
7.6 Study guidelines dealing with definition of AF	24
7.7 Study guidelines how to treat AF detected by ILR	25
7.8 Study guidelines how to treat AF detected by other methods	25
8. REMOVAL AND REPLACEMENT OF SUBJECTS	25
8.1 Removal of Subjects	25
8.2 Replacement of Subjects	26
9. ADVERSE EVENT REPORTING	26
9.1 Definitions	26
9.2 Reporting Procedures for All Adverse Events	27
9.3 Serious Adverse Event Reporting Procedures	27
10. STATISTICAL CONSIDERATIONS	27

10.1 Study Design 27
10.2 Study Endpoints and Subsets 28
10.3 Sample Size Considerations 29
10.4 Interim Analysis 29
10.5 Access to Individual Subject Treatment Assignments 30
10.6 Planned Methods of Analysis 30
10.7 Study Oversight 31
11. INVESTIGATIONAL PRODUCT 31
11.1 Loop recorder 31
11.2 Compliance 31
12. REGULATORY OBLIGATIONS 32
12.1 Ethical considerations 32
12.2 Informed Consent 32
12.3 Independent Ethics Committee 33
12.4 Subject Confidentiality 33
12.5 Investigator Signatory Obligations 34
13. ADMINISTRATIVE AND LEGAL OBLIGATIONS 34
13.1 Protocol Amendments and Study Termination 34
13.2 Study Documentation and Storage 34
13.3 Study Monitoring and Data Collection 35
13.4 Language 36
13.5 Publication Policy 36
13.6 Compensation 37
14. REFERENCES 37
15. APPENDICES 44
Appendix A – Study procedures – summary 45
Appendix B – Composition of Committees 47
Appendix C – Serious Adverse Events 48
Appendix D – Blood Samples for Genetic analysis and Blood Samples for Biomarkers 48
Appendix E – Device information: the Medtronic Reveal® LINQ 49
Appendix F – Definition of composite endpoint 51
Appendix G – Funding 52
Appendix H – Informed consent 53
Appendix I – Guidelines for oral information 54
Appendix J – Endpoint Committee Charter 56

1. OBJECTIVES:

The overall objective of this project is to improve the identification and treatment of patients at risk of stroke as well as identify how significant the contribution from AF is in stroke causation when studied by a technology which identifies all AF episodes including asymptomatic. This will be pursued by three linked studies to be performed simultaneously in a randomized clinical trial where patients at risk of stroke will be divided into two groups, with one group being continuously ECG monitored by an implantable loop recorder (ILR) and the other one not monitored.

Primary objective:

The primary objective of this study is to determine whether screening for AF with ILR and initiation of oral anticoagulation (OAC) if AF is detected, will reduce the risk of stroke and systemic arterial embolism in patients with risk factors for stroke.

Secondary objectives:

Secondary objectives include studies of whether the screening will reduce the risk of the combined endpoint of stroke, systemic arterial embolism, and mortality, studies of bleeding risks, studies of AF characterization and prediction, health economic analyses, quality-of-life assessments, cognitive function assessments, and studies of risk markers from 12-lead ECG, genetics, cardiac and brain imaging, biochemistry, and more.

Expected results:

The LOOP project is expected to provide results in three important areas:

1) It will document whether detection of asymptomatic AF in high risk patients and subsequent anticoagulation therapy will benefit patients in terms of decreased mortality and/or morbidity (AF, stroke, disability etc.), decreased hospitalisation and increased Quality of Life (QoL), not to mention psychological benefits due to being continuously monitored. Potential disadvantages are side effects caused by the ILR and medication. A demonstrated, clinically relevant benefit will have a significant impact on future patient treatment.

2) It will show whether ECG markers can be used as a means to improve identification of patients with asymptomatic AF and this will help predict which patients will develop AF during follow-up. Results will determine if this will allow the risk stratification of patients into several categories and whether patients with the highest risk need closer follow-up than those at lower risk. On a note, the same will be investigated for markers from blood biochemistry, imaging, genetics, and more.

3) It will document via a health economics analysis of whether or not introducing costly monitoring of patients in high risk of stroke events due to AF is cost-effective. The analysis will also show how

additional costs and cost savings will be distributed between various sectors and actors. The combined documentation can then be used to inform health policy decision makers.

2. BACKGROUND AND RATIONALE:

Patients with AF, diabetes, hypertension, heart failure as well as previous stroke have increased risk of stroke. Lifestyle factors such as smoking and physical inactivity are also associated with stroke. Furthermore old age is associated with increased risk of stroke. If several risk factors are present in the same patient the risk of stroke is increased further – these individuals are high-risk individuals with respect to stroke.

2.1 Stroke and AF:

Stroke is an increasingly common cardiovascular disease. Out of the 16,000 people suffering annually from stroke in Denmark, 12,000 of them are first-time strokes.[1] More than 85,000 people in Denmark live with a previous stroke.[1] By 2020 120,000 individuals are expected to be living with a previous stroke, which represents a 50% increase from 2000 to 2020.[1] Stroke primarily affects the elderly. Nearly 70% of all strokes appear in people over the age of 65. Worldwide, stroke is the reason for 10% of all deaths and causes 5.7 million deaths annually.[2] The healthcare costs due to strokes in the EU were more than EUR 38 bn in 2006, accounting for 2-3% of total EU healthcare costs.[2]

AF is the most common cardiac arrhythmia. Each year (2009) more than 21,000 people in Denmark experience a first-event AF and more than 64,000 live with an AF diagnosis,[1] the incidence of AF having increased by 35-40% from 2000 to 2009, a development that is expected to continue. Thus the number of new patients with AF is expected to double in 2020 compared with 2000.[1] Other Western countries are experiencing the same type of development.[2,3] AF is associated with having a five times greater risk of stroke.[2,3] Patients with AF-related strokes have a one-year mortality of about 30% and up to 30% of all AF-related stroke victims remain permanently disabled.[2] Thus this patient group has a higher mortality rate and suffers from strokes more disabling than non-AF strokes.[2]

A systematic search for AF primarily by checking for an irregular pulse rate can identify 50-70% more AF patients than a routine strategy based on symptom-driven diagnosis.[3] Thus, putting more effort into detecting AF will mean an increase in AF diagnosis. Previous population-based AF studies have primarily used 12-lead ECG, which only detects a minimum number of cases due to the snap-shot nature of the recording. Correct diagnosis of short-lasting AF episodes is difficult, especially if the episodes are asymptomatic. Continuous long-term ECG monitoring appears to be the ideal method to identify the true incidence of AF and to characterise its subtypes. One technology which can be used for continuous long-term monitoring is a pacemaker. This technology was used in the ASSERT Study, which recently showed that among 2,580 pacemaker patients over the age of 65 with hypertension and without a history of AF, 261 (10.1%) had device-

detected asymptomatic atrial tachycardia (mostly AF) at the three-month follow-up.[4] After 2.5 years of follow-up, 51 strokes (or embolic episodes) were detected, 11 of which were detected among 261 (4.2%) of the pacemaker patients with asymptomatic AF while 40 were among the remaining 2319 (1.7%) patients without asymptomatic AF. None of the stroke patients had symptomatic AF at the three-month follow-up.[4]

A less invasive technology which can be used for continuous long-term monitoring is the implantable ILR. This device, which measures 45 mm x 7 mm x 4 mm and weighs 2.5 g, is implanted subcutaneously on the chest wall without the need for electrodes in the heart chambers. The device samples ECG information automatically, based on specific criteria, or on-demand if the patient activates data sampling due to symptoms. Currently, ILR technology provides the best possibilities for continuous monitoring of the heart rhythm for AF detection. The fact that follow-up can be performed by telemedicine is also advantageous.

Anticoagulation therapy is effective in reducing stroke incidence in patients with AF. However, at present we do not have clear evidence about how accidentally detected AF should be weighted with respect to anticoagulation therapy.

2.2 ECG and AF risk:

Most elderly patients who develop AF have structural and functional abnormalities in the atrial myocardium.[5,6] These changes may cause heterogeneous atrial activation and a shortening of the atrial refractory period, resulting in changing patterns of cardiac impulse propagation.[7] The Framingham Heart Study showed that prolongation of the electrocardiographic PR interval is associated with an increased risk of future development of AF.[8] Our research group has recently shown that both an increment of the PR interval and an increment in QTc interval are associated with an increased risk of AF, suggesting that the PR interval, QTc interval and AF have a shared pathophysiology. The atrial repolarisation, and more specifically the atrial effective refractory period, is probably the most important factor in the pathophysiology of AF. The ventricular repolarisation time, reflected in the QT interval, is expected to be a marker of the atrial repolarisation time, as the atria and ventricles share many of the same ion channel complexes. Other ECG markers, such as P-wave duration and morphology, and premature atrial and ventricular contractions, are also associated with future development of AF.[9] This evidence suggests that future AF can be predicted from ECG markers measured on a single lead of the standard electrocardiogram.

2.3 Health economy and QoL:

The direct costs of stroke including acute care and rehabilitation are substantial. Previous studies from Denmark show that the mean cost of healthcare and social services during the first year after stroke was DKK 193,400 per person (2011 price index),[10] and that the initial severity of the stroke was the only medical factor that influenced length of hospital stays.[11] In addition to the cost of stroke treatment, rehabilitation and long term care, stroke diagnosis is associated with a significant loss of QoL for the patient.[12] Thus, interventions that prevent stroke may lead to significant cost savings that may counter balance (some of) the costs of the intervention and also improve the QoL of patients.

Several studies have explored the cost-effectiveness of various stroke rehabilitation strategies[13] and a number of studies have looked at the cost-effectiveness of secondary prevention of stroke with anticoagulation therapy in patients with AF.[14,15,16]

2.4 Study Rationale:

Patients at risk of stroke due to age, or presence of specific diseases may also have undetected atrial fibrillation. If atrial fibrillation without symptoms is detected by continuous ECG-monitoring by a Loop Recorder, this may detect atrial fibrillation, that would not otherwise be detected, and thus the patient gets a possibility to receive anticoagulation therapy, which potentially may reduce the risk of future stroke. However, anticoagulation therapy may also have undesired side effects. Therefore, we will perform a study, where we will record both the beneficial and harmful effect in a randomized trial, and we will assess economical and quality of life aspects in a randomized trial.

2.5 Clinical Hypotheses:

2.5.1 Major hypothesis:

When AF is detected and relevant anticoagulation therapy is initiated in high-risk patients, a reduction in strokes will follow. Similarly, death rate and acute hospitalisations will decrease (secondary end-points).

2.5.2 Secondary hypotheses:

a. Markers from ECG, blood biochemistry, imaging, genetics, and more, can be used to predict AF.

b. Continuous ECG monitoring with an ILR to detect asymptomatic AF and subsequent anticoagulation therapy is cost effective (ie. the cost of monitoring and therapy can be balanced against reduced costs of stroke treatment and rehabilitation, as well as improved QoL and survival).

c. Continuous ECG monitoring can detect other (non-AF) arrhythmias requiring therapy in patients at risk of stroke.

2.5.3 Supplementary hypotheses:

a. Cerebral abnormalities including magnetic resonance imaging (MRI) detected white matter hyperintensity and brain infarcts (at inclusion) are associated with AF and recurrent stroke.
b. Genetic variations are associated with development of AF and certain genetic patterns will prove to be protective against AF.
c. AF burden can predict development of cognitive dysfunction.

3. EXPERIMENTAL PLAN:

3.1 Study Design:

This is a prospective randomized, open-label (unblinded), controlled, parallel group multi-center study performed in Denmark. Participants are recruited following identification from Danish registries as well as from outpatient clinics, and after hospitalizations. The study will recruit individuals residing in Region Zealand, Region of Southern Denmark or the Capital Region of Denmark. Potentially, the study region will be increased to cover other parts of Denmark.
Participants are randomized to either receive an ILR (ILR group) or not receive one (control group). The randomization occurs at a ratio of 1:3 (ILR group vs. control group). The LOOP study will comply with the standards of the CONSORT Statement. [17]
After successfully completing all screening and baseline procedures, subjects will be randomized in a ratio of 1 to 3 to receive an ILR or usual control. The ILR will be programmed according to study recommendations (see section 6.2).
The study flow chart is given in Appendix A and the study endpoints are defined in section 10.2.
A steering committee (SC) will oversee the conduct of the study. Composition and roles of this and other committees are described in section 10.7.

3.2 Number of Centres:

The study will be a multi-centre study performed in the south and eastern part of Denmark, where all hospitals involved with implantations of cardiac implantable electronic devices (CIEDs) will be invited to participate. All participating centres will be involved in randomization, implantation of the ILRs as well as follow-up.

3.3 Number of Study Subjects:

Participants in this clinical investigation shall be referred to as subjects or patients. Approximately 6,000 subjects will be randomized in a ratio of 1:3 to receive an ILR (ILR group) or continue standard care regimen (control group).
It is expected, that the randomization period will last for 2 years. The decline rate is estimated to be <10 %. The cross over from control to the ILR group is expected to be <10 %, since an ILR implant

is generally assumed to require a syncope or definite clinical suspicion of arrhythmia or a wish to quantify arrhythmia burden. Subjects discontinuing from the study prematurely will remain in endpoint follow-up unless consent is withdrawn. Statistical considerations are provided in section 10.3.

3.4 Recruitment:

Invitation letters will be sent to approximately 15,000 individuals from the general population, identified from registers. We expect that 50% (7,500) will be interested in participating, and of these 20% (1,500) are not expected to be able to participate due to the exclusion criteria. Thus, we expect to include the planned 6,000 individuals. At each of the four hospitals 1,500 patients will be randomized (based on the assumption of 3-4 patients per working-day and 200 working-days per year).

3.5 Estimated Study Duration

The study is expected to conclude after 3 years of follow-up. The SC will continuously monitor the overall event rate and will recommend if the follow-up period should be extended, based on the number of observed primary endpoints. Based on the event rate the SC may also recommend to alter the sample size in order to complete the trial in approximately 5 years from the start of the recruitment phase.

4. SUBJECT ELIGIBILITY:

The investigators will maintain a screening log, that will include limited information about all identified potential study candidates (e.g., initials, age, sex, race, date) and outcome of the screening process (e.g. enrolled into study, ineligibility (and reason for ineligibility), or refusal to participate).

4.1 Inclusion Criteria:

To be eligible for the study, subjects must fulfil the following criteria at inclusion:

- Living address in Region Zealand, Region of Southern Denmark or the Capital Region of Denmark (study subjects from other regions of Denmark who actively contact the study for participation can be included if they wish to participate)
- 70-90 years of age at the time of screening

and additionally have at least one of the diseases mentioned below:

- Diabetes mellitus (type 1 or type 2, with or without medical therapy)
- Hypertension (with or without a therapy that may reduce blood pressure; i.e., an increased blood pressure at randomization is not required)
- Congestive heart failure
- Previous stroke (preferably diagnosed in hospital and verified by imaging; previous TIA is not considered an inclusion criterion)

Concerning the four qualifying diseases, the study subject is considered qualified for study inclusion if he has a history of one of the diseases (for example subject is now normotensive on life-style correction or medical therapy and similarly if blood glucose has been normalised upon life-style corrections or medical therapy).

4.2 Exclusion Criteria:

To be eligible for this study subjects must not meet any of the following criteria:

- History of atrial fibrillation or flutter irrespective of type
- Cardiac pacemaker or defibrillator (with or without re-synchronization therapy)
- Contraindication to oral anticoagulation therapy
- Anticoagulation therapy; vitamin K antagonists, direct oral anticoagulants, or (low-molecular) heparins. Therapy with platelet inhibitors such as acetyl-salicylic acid, clopidogrel, persantine is not considered an exclusion criterion
- Renal failure treated with permanent dialysis

- Uncorrected congenital heart disease, or severe valvular stenosis, obstructive cardiomyopathy, active myocarditis, or constrictive pericarditis.
- On a waiting list for major surgery (cardiac, thoracic or abdominal)
- Cardiac or thoracic surgery has been performed within 3 months from inclusion
- Any major organ transplant (e.g. lung, liver, heart, or kidney)
- Cytotoxic or cytostatic chemotherapy and/or radiation therapy for treatment of a malignancy within 6 months before randomization or clinical evidence of current malignancy with the following exceptions: Basal or squamous cell carcinoma of the skin, cervical intraepithelial neoplasia, prostate cancer (if stable, localized disease with a life expectancy of > 2.5 years in the opinion of the investigator)
- Life-expectancy shorter than 6 months
- Known to be human immunodeficiency virus (HIV) positive with an expected survival of less than 5 years due to HIV infection
- Recent (within 3 months) history of alcohol or drug abuse based on self-reporting
- Any condition (e.g. psychiatric illness, dementia) or situation, that in the investigators opinion could put the subject at significant risk, confound the study results, or interfere significantly with the subject participation in the study
- Unwillingness to participate or patient does not understand Danish language

Study subjects who during follow-up develop one of the mentioned diseases which qualify as an exclusion criterion at baseline will remain in the study and complete the follow-up.

5. SUBJECT ENROLMENT:

5.1 Recruitment and inclusion visit

Potential study subjects will be contacted by an invitation letter about the LOOP study with initial basic information about the study. If interested in participating in the study, potential study subjects are asked to contact one of the participating hospitals (typically the geographically closest) and an out-patient consultation is scheduled (inclusion visit).

At the inclusion visit the study subjects will be informed about the study by an investigator (or assigned designee), who will also look up the subject’s medical records, and will interview the patient with a focus on previous medical history (diabetes, heart disease, hypertension, AF etc.) to assess study eligibility.

Blood pressure will be measured, and an ECG and blood sample is taken. Baseline variables including CHA_2DS_2-VASc and NYHA class score will be recorded. Individuals with AF on the ECG will be excluded from participating and these patients will be referred for relevant treatment.

Subjects will be screened for previously unknown diabetes and hypertension. If these diseases are detected the patients will be referred for further tests or therapy. Individuals who wish to participate will sign informed consent documents, complete the QoL questionnaires and The Montreal Cognitive Assessment (MoCA) tests, and will be randomized to either implantation of ILR or standard care. Subjects must personally sign and date the consent form.
Patients may have additional study tests performed (T-wave alternans test, echocardiography, MRI etc.).
For the purpose of this trial the following definitions will apply:
Registration
A subject has signed the informed consent form and has been assigned a subject identification number generated by the computer generating code (but not yet randomized)
Randomization
A subject has been assigned to one of the two treatments and has been assigned a randomization number.
Enrolment
A subject is considered enrolled once the subject is randomized and the ILR implantation (or control) has been performed.

5.2 Subject Registration:

After the provision of informed consent each subject will be registered in a web-based electronic eCRF (eCRF), protected by encryption. A subject identification number will be assigned during registration, and will be used to identify a subject throughout the trial and must be used on all study documentations related to that subject. The subject identification number must remain constant throughout the entire trial; it must not be changed at any time.
All entries in the eCRF up to and including randomization are performed at the participating centre. Subsequent follow-up visits can be entered at the participating hospital where the patient is being followed. Subject initials and date of birth as well as other information will be required for registration, including for example subject initials and date of signed informed consent.

5.3 Screen Failures:

Registered subjects, who are ineligible for the study based on screening assessments will be considered screen failures and entered as such into the eCRF. The following information will be required:

- Subject identification number
- Date of birth

- Reason for failing screening
- Date of last screening assessment

5.4 Randomization and Treatment Assignment

Subjects, who successfully complete the screening assessment, and meet the eligibility criteria, will be randomized into the study. A computer generated randomization scheme will be generated to assign subjects to treatment, and those who fulfil the eligibility criteria (section 4) will be randomized in a ratio of 1:9 to receive an ILR or to be a control, respectively. The investigator (or assigned designee) will access the central computer and obtain an assignment code at the randomization visit. The following information at a minimum will be required for randomization:

- Subject identification number
- Date of birth
- Confirmation, that the subject meets the eligibility criteria

This information will be entered into the eCRF. Subject must be randomized within 30 days of being identified at the referral hospital.

5.5 Re-Screening:

Registered subjects, who do not meet all eligibility criteria on the first screening occasion may be rescreened for the study at a later time. Such subjects may begin screening procedures again two weeks after the date of the last screening assessment. Rescreened subjects will retain the originally assigned subject identification number: However, the rescreening attempt will need to be entered into the registration computer.

6. TREATMENT PROCEDURES:

Implantation of a Loop Recorder (ILR) will be performed according to local standards and within four weeks of randomization.

6.1 ILR device:

Devices from Medtronic (Reveal LINQ or newer comparable devices) with capability for remote monitoring will be used in this trial. Information on specific ILR data can be found in Appendix F.

6.2 ILR procedures:

6.2.1 Preparations and setting:

Preoperative biochemical parameters as required by local guidelines (which may include variables such as INR, blood cells, creatinine, sodium, potassium).

The device is implanted at the hospital using standard sterile technique; not necessarily in an operation or cardiac catheterization facility. An intravenous injection line (such as Venflon) is optional, but is inserted if i.v. antibiotics are given.

6.2.2 Medication in relation to surgery:

Antibiotics are given according to study guidelines. It is suggested to give at least one dose of i.v. cefuroxime (Zinacef) 1.5 g or dicloxacillin (Diclocil) 0.5-1.0 g immediately prior to the procedure, or oral dicloxacilling (Dicillin) 0.5-1.0 g at least one hour prior to the procedure. It is optional to give additional antibiotics. In case of allergy to penicillin alternative antibiotics is recommended according to local guidelines. Sedatives are not recommended. Local anaesthesia is given according to each centre’s local guidelines.

6.2.3 The surgical procedure:

The ILR is positioned subcutaneously on the left side of the chest using supplied incision and insertion tools. Measurement of the device orientation is optional. Securing optimal electrocardiogram before wound closure is recommended. At each centre, the implantation of the ILR is performed by either a senior cardiologist or a medical doctor under general supervision of the local senior cardiologist.

6.2.4 Loop Recorder programming:

The ILRs should be programmed according to study guidelines, and details are specified in Appendix E.

6.2.5 Post operative monitoring:

After the implantation, one hour of observation at the facility is recommended , and after this the patient can be discharged from hospital. The patients will receive advice with respect to skin inspection in the surgical region, treatment of local pain after surgery.

6.2.6 Remote follow-up:

Study participants will be instructed in the automated daily remote transmissions which require bedside setup of the MyCareLink Patient Monitor at their home along with patient-initiated transmissions to transfer extra data .

6.3 Concomitant Therapy:

Throughout the study investigators may prescribe any concomitant medications or treatments necessary to provide adequate supportive care. Information on concomitant therapy will be collected on the appropriate case report form.

If the ILR monitoring detects severe bradycardia, ventricular tachyarrhythmia or other arrhythmia these will be dealt with according to clinical guidelines. For study guidelines how to treat AF, see section 7.7.
It is possible for the patient to participate in another trial, both including devices (such as stents) or medical therapy.

6.4 Prescribed therapy during the study period:
All subjects (both ILR group and control patients) should receive optimal medical therapy for their inclusion criteria disease (hypertension, diabetes, heart failure or stroke). The national guidelines for treatment of heart failure and hypertension are described in the guidelines from the Danish Society of Cardiology which can be downloaded from the web page www.cardio.dk. The standard regimen for stroke patients is described in the Danish Reference Programme 2013 (Dansk Apoplexi Selskab).

7. STUDY PROCEDURES:

A tabular schedule of study procedures is given in Appendix A. Written informed consent must be obtained before performing screening procedures. The investigator is responsible for ensuring that all assessments are performed according to the protocol and that the appropriate data are recorded in the eCRFs. Missed visits, test(s), that are not done and examinations, that are not conducted, must be reported on the eCRFs.

7.1 Subject Screening:

During screening each subject will be evaluated for inclusion in the study. All laboratory assessments scheduled for screening will be done by the local laboratory. The following procedures, screening evaluations and assessments will be done once within 21 days prior to randomization:

- Obtaining or verifying informed consent
- Medical history, including previous, surgical procedures
- NYHA functional classification: The severity of heart failure will be evaluated by the investigator or the designee at the respective study visits. If possible a subject should be evaluated by the same person throughout the study.
- CHADS-VASc score
- Physical examination, including height, body weight
- Ongoing medical therapy
- Blood samples for routine biochemical testing, plasma troponines, p-BNP and for storage in the study related research biobank. After analysis of the material from the study related research biobank surplus biological material is transferred to a biobank for future research.
- 12-lead ECG

If laboratory samples are missing, destroyed, unable to be analysed or results are considered and documented by the investigator as being obviously incorrect, a subject may be re-tested.

7.2 Follow-up Phase:

7.2.1. The main study:

The ILR devices will be implanted over the course of 12-24 months (1-8 implantations per week in each of the centres), starting at the beginning of 2014.

- Participants in the ILR group will be seen at in-hospital visits at 12, 24 and 36 months after ILR implantation. Between in-hospital visits they will be followed by daily automatic ILR

remote telemedicine transmissions. Data from the ILRs are automatically transmitted to a central database. Every day, an experienced medical doctor reviews the recent transmissions in the database. Subjects will be contacted if transmissions are not received in a period of 14 days, and any system malfunctions will be corrected. If a new-onset AF episode lasting minimum 6 minutes is detected, two senior cardiologists independently evaluate the transmission. In case of disagreement, a third senior cardiologist will independently evaluate the episode. If the two or two of three cardiologists confirm the diagnosis of AF, OAC will be initiated by the local center (see section 7.8), and this will be registered in the eCRF. Remote monitoring will continue until the end of battery life, study withdrawal, or death occurs. At in-hospital visits, QoL and MoCA forms will be filled. Adverse events will be collected at study visits and by lookup in medical records and registries at least on a yearly basis until the end of study.

- Participants in the control group will be seen only at inclusion and after the 3 year follow-up. During this period they will be seen by their general practitioner. Between the scheduled visits QoL forms will be sent by mail. MoCA forms will be filled in on the final follow-up. Adverse events will be collected at study visits and by lookup in medical records and registries at least on a yearly basis until the end of study.

Additional visits are made at the discretion of the caring physician. Throughout the study the investigator or study nurse/coordinator should continue to provide appropriate medical care.

The following assessments and procedures will be performed at specified time points during the study and will be recorded in the eCRFs (for details please see Appendix A, section a and b):
- CHADS-VASc score: At randomization.
- Blood sample: At randomization. Some participants might have additional blood samples taken.
- NYHA functional classifications: At randomization and (12, 24,) 36months.
- Assessment of QoL and cognitive function : At randomization, months 12, 24 and 36.
- Vital signs (BP, HR …): At randomization and at (12, 24), 36 months.
- 12-lead ECG: At randomization and at (12, 24), 36 months.
- Concomitant therapy: At randomization and months 12, 24, 36 and by lookup in medical records and registries at least on a yearly basis.
- Remote Loop Recorder readings: Daily
- Adverse events (AEs): At clinical visits and by lookup in medical records and registries at least on a yearly basis (see section 9 for definitions).

Information on all AEs, that occur from study day 1 after randomization will be recorded in the appropriate eCRFs on study visits and by lookup in medical records and registries at least on a yearly basis. All serious adverse events (SAEs) will be reported to the study office within one working day of discovery (see appendix C), and a SAE form must be completed (see section 9.3 for definitions). Bleeding complications will be registered according to the suggestions by the International Society for Thrombosis and Haemostasis (Schulman and Kearon, 2005). For the efficacy analysis of mortality, the endpoint committee (EC) will review all deaths and classify the events as cardiovascular or non-cardiovascular (see Appendix J). Cardiovascular deaths will be further classified as sudden and non-sudden based on a time criteria (see Appendix J). Adjudicated cause of death as well investigator-reported cause of death will be entered into the eCRF.

A subgroup of those patients that underwent an echocardiography at inclusion will be invited to participate in a similar echocardiography during follow-up. The criteria for invitation is that the participants should have been included in the LOOP study before January 2017 at one of the study centres Rigshospitalet, Bispebjerg Hospital or Roskilde Hospital, and that the first echocardiography should be between 2 and 6 years old at the time of follow-up echocardiography.

For endpoints, see section 10.2

7.2.1 The ECG study:

The ECG computer analysis study will collect periodic data from the ILR during sinus rhythm and during episodes of AF (both patient-activated and automatic sampling). Patients in the ILR group will be encouraged to make a monthly patient-activated sampling after five minutes of rest (most likely in sinus rhythm). Standard 12-lead ECGs will be obtained at baseline (ILR and control groups) and also annually in the ILR group. ECG markers predictive of future AF will be derived from the recordings.

<u>Objectives:</u>

Primary: To study the the association between chronological developments of ECG markers measured by ILRs and incident AF, in order to identify which patients fall into low or high risk AF groups.

Secondary: Calculation of daily AF burden (ILR group) to refine quantification of episodes beyond merely presence/absence and to guide decisions regarding the need for anticoagulation. Relationship between AF burden and development of ECG markers.

Additional ECG studies:

- Incidence rate and timing of new onset AF to identify the necessary duration and regularity of ECG monitoring to reliably identify change in status.
- Throughout the ECG study we will also monitor the frequency of symptom triggered ILR transmissions, the proportion of falsely detected AF episodes by ILR as well as the number of planned ECG samplings and data transmissions from ILR devices which are not received.
- Bradyarrhythmia and ventricular tachyarrhytmia.

7.2.2 The health economic study:

The health economic analysis is appended to the analysis of the randomized clinical trial.

Objectives:

Analysis of changes in the use of healthcare resources and costs, i.e. hospital treatment, general practitioner services, medical specialists, pharmaceuticals, rehabilitation, and nursing homes, and analysis of quality-adjusted life-years (QALYs).

Comments:

Individual personal data on resource use and health outcomes will be collected prospectively during the trial or from national registers, which also continuously record healthcare consumption. The framework for the analysis is a cost-utility analysis where the mean differences in costs and QALYs between the intervention and the control group is expressed as a ratio that shows the cost per QALY gained from the introduction of ILR monitoring and subsequently AF management. All costs will be estimated from a societal perspective.

Resource use data in relation to the implantation of ILR, ILR data transmission and analysis, as well as follow-up visits, will be recorded prospectively. The registration will include noting the amount of time staff spend during pre-operative evaluation, procedures (e.g. blood tests, electrocardiogram), utensils, anaesthetics, sedatives, antibiotics and hospital stay for each participant. In order to estimate costs, information about salaries, the cost of utensils, pharmaceuticals and equipment will be collected from the hospital finance department.

Data on the participants' use of inpatient and outpatient hospital care and use of emergency departments, general practitioners and other private practising medical specialists, physiotherapists, chiropractors, chiropodists and psychological counsellors, as well as prescription drugs, will be collected from the National Patient Register, the National Insurance Service Register, and the Register of Medicinal Product Statistics. It is assumed that costs of healthcare consumption unaffected by the intervention will cancel out due to randomization. Furthermore, it is assumed that all AF management costs will be captured in the register data.

Hospital treatment will be valued with official hospital charges (diagnosis related group (DRG) charges) provided by the National Board of Health. Special attention to the relevance of using DRG charges will be considered.[21] Services from private practising providers will be valued according to the prevailing fee schedules that are agreed upon between the providers and the regional health authorities. For the valuation of general practitioner services an annual amount will be added to take into account that general practitioners are paid by a combination of capitation and fee-for-service. Furthermore, patients' co-payments will be added. Data on the participants' use of prescription drugs will be collected from the Register of Medicinal Product Statistics. These drugs will be valued according to the pharmacy sales prices.

Information about the participants' use of services provided by the municipalities, e.g. community nurse visits, personal care and practical assistance at home, nursing home, respite care and rehabilitation, will be collected from the participants' municipality of residence. Unit costs of these services will be estimated based on statistics on wage rates and databases that record the cost of providing home care, (e.g. The Free Choice Database (*Fritvalgsdatabasen*)), which is administered by the National Board of Social Services. All costs will be converted to a specific year price level using the consumer price index and other relevant price indices.

QALYs will be estimated with the use of the EQ-5D-5L instrument.[12] Participants will fill in the EQ-5D-5L questionnaire at baseline (before operation) and every second year (mailed to control group). Each health state is converted to a social tariff (utility score) from a representative sample of the Danish population.[22] This will be used to construct each participant's utility profile. A straight line relation between each of the observed utility scores will be assumed. The number of QALYs experienced by each participant will be calculated as the area under the participant's utility profile.

Descriptive analyses will be used to describe the mean QALYs, resource use and costs per participant in both arms of the trial. Differences in QALYs, resource use and costs between the two groups after 24 months of the study will be based on the trial data. Confidence intervals of 95% for differences between the two groups will be computed based on bootstrap re-sampling with 5,000 replications of the trial data.

For the cost-effectiveness analyses, the incremental cost-effectiveness ration (ICER) will be estimated on the basis of the mean difference in costs relative to the mean difference in QALYs. Uncertainty about the ICERs will be analysed using bootstrapping and presented in a cost-effectiveness plane. A cost-effectiveness acceptability curve will be generated to represent the probability that ILRs are cost-effective compared with usual treatment. Estimates of the costs per QALY gained from ILR will be compared to similar estimates of rehabilitation after stroke, AF management as well as other cost-effectiveness estimates in other treatment areas.

Study nurses will register all contacts between study subject and study nurse in the hospital files to be included in the cost evaluation.

7.2.3 The MRI study:

Patients with risk factors for stroke frequently have asymptomatic previous brain infarcts.[19] Age, hypertension and diabetes predispose to white matter hyperintensities, which is associated with cognitive disorders, balance and mood disorders.[20] In a subset of the study participants (approximately 500 subjects) we will perform MRI of the brain upon study entry to detect previous brain infarcts (semi-quantitative assessment) and white matter hyperintensities (semi-quantitative assessment). MRI scans will be performed <u>withou</u>t contrast injection. In the patients in whom

stroke occurs during follow-up a second MRI should be performed close to the time of stroke. In the subjects who had a first MRI scan performed a final MRI should be performed at the end of the study. MRI scans of brain are performed with 3 sequences: FLAIR , DWI and Gradient Echo sequencing. In a subset of participants we will perform an MRI scan of the heart with Gadolinium contrast to measure chamber dimensions, wall-motion and late enhancement (fibrosis) (approximately 200 subjects).

7.3 Endpoint Determination:

7.3.1 Definition of Endpoints:

Please see Section 10. Details are provided in Appendix F.

7.3.2 Data Collection:

Endpoint data will be collected on specific eCRFs. For all possible primary endpoints, and all deaths, all supporting documentation from medical records, imaging, laboratory tests, registries, etc., will be collected, and will be provided to the EC during the course of the trial for review and confirmation of events as outlined in the EC charter.

7.4 Genetic analysis and Biomarkers:

At randomization 3 types of blood vials will be collected (whole blood sample, EDTA while sample, and serum sample). Please see Appendix D for handling of blood samples. Blood samples will be used for genetic and biochemical analyses.

Other markers will be acquired as described above: ECG, and MoCA test (and imaging in a subset of patients) .

7.5 Strategy for management of low inclusion rate:

1) Too low patient recruitment due to healthcare professionals or logistics: We will continuously monitor patient inclusion each month per centre. If a centre does not recruit the expected number of patients we will recruit a new centre. In the interim period the other centres will increase their recruitment.

2) Too low patient recruitment due to patient preferences: We conducted a small survey asking potential candidates if they would participate if asked, and patients were positive. A logbook will be used to list and identify reasons why patients decline to participate. If better information can increase the participation rate we will improve the quality of the information. Otherwise additional letters of invitation will be sent.

3) Patients cannot perform telemedicine transmissions: Rigshospitalet is currently performing another study with ILRs in elderly patients with valve disease (aortic stenosis). These patients are

fully capable of performing telemedicine transmissions. In the event of problems, study nurses will guide the patients by telephone.

7.6 Study guidelines dealing with definition of AF:

Arrhythmia which the device has sampled and categorised as AF and which is afterwards:

- confirmed as AF by a study investigator and
- has a duration of ≥ 2 minutes (please note that this is not the same as AF which requires anticoagulation therapy)

After new AF has been diagnosed the patient will continue unaltered arrhythmia monitoring as previously performed. The device requires 2 minutes for AF detection.

7.7 Study guidelines how to treat AF detected by ILR:

When AF is detected by the ILR and the episode is verified by an investigator and has a qualifying duration (i.e. an episode which has a duration ≥ 6 minutes), the patient is given advice to start therapy with one of the newer OAC (preferred strategy) or conventional OAC according to local routine.

It is recorded if the patient actually will receive OAC. Subjects will be covered by normal patient insurance.

7.8 Study guidelines how to treat AF detected by other methods (also relevant for the control group):

When AF is diagnosed in the control group or in the ILR group by non-ILR methods (including monitoring during hospitalisations, Holter, ECG, etc) patients are recommended to start anticoagulation according to the same guidelines as mentioned in the section above (section 7.8).

8. REMOVAL AND REPLACEMENT OF SUBJECTS:

8.1 Removal of Subjects:

A subject has the right to withdraw from the study at any time, for any reason, without prejudice to his or her future medical care by the physician or at the institution. Any subjects who withdraws consent to participate in the study will immediately be removed from further treatment and/or study observations on the date of request. The investigator has the right to withdraw the subject from the study, if any of the following occurs:

- Significant illness caused by the ILR
- Refusal by the subject to continue observations

- Decision by the investigator, that termination is in the subjects best medical interest
- Loss to follow-up

Should a subject (or legally acceptable representative) request or decide to withdraw from the study, all efforts will be made to complete and report the observations as thoroughly as possible up to the date of withdrawal. All information should be reported on the applicable eCRF. A complete, final evaluation and assessment (section 7.4) should be made at the time of the subject’s withdrawal. The end of study eCRF will be completed with an explanation for the withdrawal. If the withdrawal of a subject is due to an AE, follow-up visits should be scheduled until the AE has resolved or stabilized. Unless consent has been withdrawn, follow-up date on death and hospitalizations will be collected until study termination, though if specifically requested by the patient, the patient will not be followed for the primary endpoint until end of study.

8.2 Replacement of Subjects:
Randomized subjects will not be replaced if discontinued.

9. ADVERSE EVENT REPORTING:

9.1 Definitions:

9.1.1 Adverse Events:
An AE is defined as stroke (hemorrhagic or ischemic), peripheral emboli, bleeding, cardiac event (atrial fibrillation, ventricular or supraventricular tachyarrhythmias, bradycardia, pacemaker implantation, myocardial infarction, unstable angina, percutaneous coronary intervention, coronary artery bypass graft or heart failure) or ILR infection, occurring in a subject after the initiation of the study, whether or not considered to be related to the implantation of the ILR. Elective hospitalizations (i.e., due to any pre-existing systemic illness or elective surgery) are not AEs. Abnormal laboratory values should not be reported as AEs, unless they fulfil the definition above.

9.1.2 Serious Adverse Events:
A serious adverse event is defined by regulatory agencies as an AE that poses a significant hazard or side effect, regardless of the investigators or sponsors opinion on its relationship to the investigational product. This includes, but may not be limited to, any event, that:

- Is fatal, or

- Is life threatening (places the subject at immediate risk of death) , or
- Requires in-patient hospitalization or prolongation of existing hospitalization, or
- Leads to persistent or significant disability/incapacity

A hospitalization meeting the regulatory requirement for “serious” criteria is any in-patient hospital admission, which includes a minimum of an overnight stay in a health care facility. Any event, that does not exactly meet this definition, which in the investigators opinion represents a significant hazard, can be assigned “other significant hazard” regulatory reporting serious criteria. Additionally, important medical events that may not be immediately life-threatening or result in death or hospitalization, but which may jeopardize a subject or require intervention to prevent the outcomes listed above, or result in urgent investigation, may be considered serious. Examples include allergic bronchospasm, convulsions or blood dyscrasia. Serious Adverse Events will be reported based on patients information to study personal or monthly registry retrievals and will be filed in CRFs by the local study team.

9.2 Reporting Procedures for All Adverse Events:
All AEs occurring after randomization observed by the investigator or reported by the subject (whether or not attributed to the investigational product), will be reported on the electronic case report form. The following attributes must be assigned by the investigator:

- Diagnosis.
- Description: E.g. hospital records.
- Dates of onset and resolution and action taking.

The investigator may be asked to provide follow-up information. All AEs, serious or not, that result in a subjects permanent withdrawal from the study must be reported in the CRF. The end-of-study eCRF will be completed, giving details of the withdrawal.
Medically significant AEs considered related to the investigational product by the investigator or the sponsor will be followed until resolved or considered stable.

All deaths occurring in study must be recorded, and cause of death must be investigated. This includes deaths until determination of the study or a maximum of 60 months since enrolment of the subject, whichever is earlier.

It will be left to an investigators clinical judgement as to whether or not an AE is of sufficient severity to require a subjects removal from treatment.

9.3 Serious Adverse Event Reporting Procedures:

All SAEs must be reported in the electronic CRF within one working day of discovery or notification of the event. Initial SAE information and all amendments or additions must be recorded on a Serious Adverse Event Report form.

Description of SAEs reporting is included in Appendix C.

10. STATISTICAL CONSIDERATIONS:

10.1 Study Design:

This is a prospective randomized, open-label (unblinded), controlled, parallel group multi-center study performed in Denmark. Patients are recruited following identification from Danish registries as well as from outpatient clinics, and after hospitalizations. The study will recruit individuals who live in the Capital Region of Denmark, Region Zealand or the Southern Region of Denmark. The study is expected to conclude when 279 strokes have occurred or 6,000 study subjects have been followed for at least 36 months, which is expected to be approximately 5 years after enrolment of the first subject. Subjects meeting the eligibility criteria will be randomized to either receive an ILR (ILR group) or not ie, continue standard care (control group). The randomization occurs at a ratio of 1:3 (ILR group vs. control group) as described in section 5.2. The LOOP study will comply with the standards of the CONSORT Statement.[17] The study design is further described in section 3.

10.2 Study Endpoints and Subsets:

10.2.1 Primary Endpoint:

Efficacy (time to at least one of the components of the combined primary endpoint):

- adjudicated stroke <u>or</u>
- adjudicated systemic arterial embolism

10.2.2 Secondary Endpoints:

- time to adjudicated ischemic stroke/transient ischemic attack/systemic arterial embolism
- time to adjudicated stroke, systemic arterial embolism, or cardiovascular death
- time to adjudicated cardiovascular death
- time to death by any cause

10.2.3 Other Endpoints:

- time to diagnosis of AF

- time to initiation of OAC
- time to adjudicated intracranial hemorrhage not classified as stroke
- time to adjudicated hemorrhagic stroke
- time to major bleeding as defined by the International Society on Thrombosis and Hemostasis
- complications related to loop recorder implantation

10.2.4 Supplementary Endpoints:

- Time to first acute (non-elective) hospitalization for stroke, systemic arterial embolism, transient ischemic attack, bleeding, cardiac disease, or complications, requiring an overnight stay in hospital or resulting in death
- Time to first admission for acute coronary syndrome or myocardial infarction
- Time to first implantation of cardiac pacemaker or defibrillator (with or without re-synchronization therapy)
- Time to first ablation of cardiac arrhythmia
- Development of AF burden
- Development of other cardiac arrhythmias
- ECG markers in predicting AF
- Changes in health economic costs
- Change from baseline QoL, and Quality Adjusted Life Years
- MRI: Change in occurrence of brain infarcts and white matter hyper-intensity
- MRI: Left atrial size, fibrosis, and function and the association with stroke and AF
- Safety aspects (such as infections) of ILR implants
- Inflammation markers and cardiac hormones and their prediction of AF and stroke
- Changes in cognitive dysfunction evaluated by MoCA forms
- Genetic prediction of AF and stroke
- Echocardiographic prediction of AF and stroke

10.2.5 Subsets:

Efficacy Analysis Set:

Efficacy analysis will be performed on the intent to treat (ITT) subject set, defined as comprising all subjects randomized. Efficacy analysis will include all available follow-up data from all randomized subjects. In addition sensitivity analysis may be carried out based on the un-treatment subject set, which is defined as subjects not receiving ILR regardless of randomization.

10.3 Sample Size Considerations:

We expect that 30% of the participants will have documented AF episodes lasting ≥6 minutes (and per protocol qualifying for OAC) during ILR monitoring for at least three years. The study population is expected to have a stroke rate of 0.7%/year in non-AF patients, and 2%/year in AF patients not treated with OAC. While we expect the number of AF-related strokes to be reduced by OAC, we assume that strokes unrelated to AF will not be influenced by this. We also include the assumption that 3% of all participants in the control group will become diagnosed with AF and start OAC. The annual rate of the primary end point in the control group is thus expected to be 1%. Finally, we expect that 5% in the ILR group will not be monitored due to refusal of ILR implantation or ILR explantation due to discomfort. These patients will participate at the same risk as patients in the control group and will be included in the analysis of the primary endpoint according to the intention-to-treat principle.

We thus expect the overall hazard ratio to be 0.65. To analyse this reduction in the primary endpoint with a 2-sided α-level of 0.05 and a power of 80% in a 1:3 intervention:control-randomized population, we will need 279 primary events. Follow-up duration will be adjusted to accommodate the number of primary events. With an assumed length of inclusion period of 1-2 years and a median follow-up up 4 years, 6000 subjects will be needed.

10.4 Interim Analysis:

The likely hood of observing a statistically significant beneficial treatment effect is dependent on the event rate in the trial. When approximately 1/3 of the subjects have completed one year of follow-up this event rate will be discussed by the SC in order to decide, if the study should continue as planned. The SC has the right to increase sample size or prolong follow-up if needed.

10.5 Access to Individual Subject Treatment Assignments:

Individual subject treatment assignments will be given to the SC if requested. Un-blinded (presented as group A and B) results, including aggregate and subject level data, can be presented to the SC for safety data reviews.

10.6 Planned Methods of Analysis:

10.6.1 General Approach/Considerations:

Baseline characteristics, demographics, pre-existing conditions, previous illnesses, and possible prior treatments will be summarized for each treatment group. Subjects disposition and reason for discontinuation will be summarized for each group. The primary and secondary efficacy endpoints

are timed to event variables and survival techniques will be utilized to perform statistical estimation and hypothesis testing. Concomitant medications will be summarized for each treatment group and compared between groups to access their potential impact on the primary endpoint.

10.6.2 Analysis of Key Study Endpoints:

10.6.2.1 Primary Endpoint:

The principle analysis for the primary endpoints will employ the intend-to-treat principle, and use a survival analysis. To account for the competing risk of death, the cumulative incidences of the primary endpoint will estimated, plotted and group-wise compared in a multi-state fashion. A co-variate adjusted analysis of the combined primary endpoint using a cause-specific Cox proportional regression model will be performed as a supportive analysis. The hazard ratios and the corresponding 95% confidence intervals will be estimated. Subjects completing the study and not reaching the composite endpoint will be censored.

10.6.2.2 Secondary Endpoints:

See section 10.2.2.

For the time-to-event variables, analysis similar to the one employed for the primary endpoint will be used. The censoring mechanism will also be similar to the one used for the analysis of the primary endpoint. The secondary endpoints will be tested at a significance level of 0.05. Within the first two secondary endpoints the type 1 error rate associated with a multiplicity of comparisons will be controlled according to the Hochberg adjustment. The Quality of Life endpoint will be tested at the 5% significance level. Quality of Life score will be analysed as repeated measures analysis of covariance mixed effects model using baseline score as a covariate.

10.6.2.3 Safety Endpoint:

AEs: Subjects incidence rates of all AEs will be tabulated by system class,
preferred term, severity, and relationship to investigational product. Tables and/or narratives of "on-study" death, and serious and significant AEs, including those causing early
withdrawal, will also be provided.

Complications from ILR implantation will be asked for and registered at all visits.

10.7 Study Oversight:

10.7.1 Steering Committee:

A SC comprising experts in the cardiovascular field will provide oversight and advice to ensure the most safe and efficient conduct and execution of the trial. The SC will consist of external physicians with experience and expertise in the design and conduct of cardiovascular clinical trials and will have representations from the working group on pacing and electrophysiology of the Danish Society of Cardiology, as well as representatives from Danish Stroke Neurologists. The

main focus of the SC will be on the medical, ethical and scientific integrity of the study and on its timely completion. The SC will consider sample size re-examination in the eventuality at the observed, blinded pooled event rate for the primary endpoint falls below the assumptions from the design stage. SC members may be investigators in the study. To facilitate the communication of results from the study, a publication committee will be formed from the SC at an appropriate time, consisting of members of the SC and of others, who have contributed significantly to the study.

10.7.2 Endpoint Committee:

An endpoint committee (EC) will be formed prior to study commencement to adjudicate the primary endpoints in a blinded fashion (see Appendix B and F). A neurologist and a cardiologist are appointed in the EC.

With respect to correct classification of recorded AF events an “AF adjudication committee” will be established among the cardiologists in the SC. “The AF adjudication committee” will be composed of two cardiologist (rotation among SC members will occur). If these two cardiologists disagree a third cardiologist will be involved for final adjudication of AF.

11. INVESTIGATIONAL PRODUCT:

11.1 Loop recorders:

The ILR used in this study is the Reveal LINQ (Medtronic) or newer devices with similar functionality. The device is described in details in appendix E.

The device is capable of remote monitoring.

11.2 Compliance:

All patients randomized to ILR should undergo implantation. However, the number of patients randomized but not receiving an ILR will be registered as well as the number of explanted ILRs.

12. REGULATORY OBLIGATIONS:

12.1 Ethical considerations:

It is currently not known how asymptomatic AF should be treated. Only when all (“the majority of”) AF is diagnosed the scientific and clinical problem about how to deal with asymptomatic AF can be addressed properly. One approach in this would be to perform continuous long-term monitoring of patients at risk. Therefore the LOOP study will answer clinically relevant questions and is based on this background.

In the present study patients will be randomized to either receive an ILR or not. For patients randomized to the control group, there is very little burden by the study since there is no placebo treatment. Patients randomized to ILR have the potential advantage of having AF detected and subsequently receiving appropriate anticoagulation therapy, which may turn out to be beneficial. However, such treatment may also cause potentially serious side effects.

Study patients randomized to the ILR group may have local complaints due to surgery and the insertion of a device subcutaneously. They will have a small scar after surgery. There is a small risk of infection in the region of surgery. They may have diagnosed atrial fibrillation (which is the purpose of the study) without symptoms and experience anxiety due to knowing that this was detected even though it does not give symptoms. They may start anticoagulation therapy and this may give side-effects such as bleedings, which would not have occurred if they had not participated in the study. Also, EGG artefacts may be misdiagnosed as atrial fibrillation.

Patients may experience that they become more focused on their health situation due to frequent data transmissions. On the other hand, patients may experience that it gives them a feeling of safety. Patients in the control group may feel unhappy since they are not followed as closely as they had hoped.

This study will require approval from the Regional Danish Committee on Biomedical Research Ethics, as well as allowance from The Danish Data Protection Agency. The Danish Medicine Agency has been asked if application for permission to perform the study was necessary, and the Agency has answered that an approval of the LOOP study was not necessary.

Overall the study addresses an important clinical problem and if the trial turns out positive it will improve therapy for a large number of patients. Since the socio-economic costs in therapy and care related to stroke are enormous the results from the study may also be important for society. Therefore, we consider the risks we impose on patients are acceptable.

12.2 Informed Consent:

An informed consent form is provided in Appendix H. Guidance for oral information of participants is given in Appendix I.

Before a subject's participation in the trial, the investigator is responsible for obtaining written informed consent from the subject after adequate explanation of the aims, methods, anticipated benefits, and potential hazards of the study, and the right to withdraw without consequences, before any protocol-specific screening procedures or any investigational products are administered. The potential benefit and hazard associated with participation in the study is described in the informed consent form (Appendix H). If the subject agrees, it is recommended that the investigator informs the subject's primary care physician of his/her participation in the clinical trial.

The acquisition of informed consent should be documented in the subjects medical records, and the informed consent form should be signed and personally dated by the subject, and by the person who conducted the informed consent discussion (not necessarily an investigator). The original signed informed consent form should be retained in accordance with institutional policy, and a copy of the signed consent form should be provided to the subject or the subject's legally acceptable representative.

If a potential subject is illiterate or visually impaired, the investigator must provide an impartial witness to read the informed consent form to the subject, and must allow for questions. Thereafter, both the subject and the witness must sign the informed consent form to attest that informed consent was freely given and understood.

12.3 Independent Ethics Committee:

A copy of the protocol, proposed informed consent form, other written subject information, and any proposed advertising material must be submitted to the Ethics Committee for the Capitol Region of Denmark. The investigator must submit and, when necessary, obtain approval from the Ethics Committee for all subsequent protocol amendments and changes to the informed consent document.

12.4 Subject Confidentiality:

The investigator must ensure that the confidentiality of a study subject is maintained. On the eCRFs or other documents mailed to the study office, subjects should be identified by their initials and a subject study number only. Documents that are not for submission (e.g. signed informed consent forms) should be kept in strict confidence by the investigator. In compliance with Federal

regulations/ICH-GCP Guidelines, it is required that the investigator and institution permit authorized representatives of a monitoring company, and the EC, direct access to review a subjects original medical records for verification of study-related procedures and data. Direct access includes examining, analyzing, verifying, and reproducing any records and reports that are important to the evaluation of the study. The investigator is obligated to inform and obtain the consent of a subject for named representatives to have access to his/her study-related records without violating the confidentiality of the subject. All information obtained in relation to this protocol regarding participating patients is protected according to Danish law on Patients Legal Rights and Data Protection.

12.5 Investigator Signatory Obligations:

Each clinical study report should be signed by the investigator or the coordinating investigator. The coordinating investigator meets at least one of the following criteria:

- A recognized expert in the therapeutic area
- An investigator, who provided significant contributions to either the design or interpretation of the Study

13. ADMINISTRATIVE AND LEGAL OBLIGATIONS:

13.1 Protocol Amendments and Study Termination:

Protocol amendments must be made only with the prior approval of Steering Committee. Agreement from the investigator must be obtained for all protocol amendments and amendments to the informed consent document. The Ethics Committee must be informed of all amendments and give approval for any amendments likely to affect the safety of the subjects or the conduct of the trial. The Steering Committee reserves the right to terminate the study. The investigators reserve the right to withdraw from the study.

13.2 Study Documentation and Storage:

The investigator should maintain a list of appropriately qualified persons to whom he/she has delegated trial duties. All persons authorized to make entries and/or corrections on eCRFs will be included on the sponsor Delegation of Authority Form. Source documents are original documents, data, and records from which the subject’s eCRF data are obtained. These include but are not limited to hospital records, clinical and office charts, laboratory and pharmacy records, diaries, microfiches, radiographs, and correspondence. CRF entries may be considered source data if the eCRF is the site of the original recording (i.e., there is no other written or electronic record of data).

In this study, patient reported outcome information can be recorded directly on the web based CRF. It will be possible to perform electronic signature in the web based CRFs.The access to the web based CRFs will be protected by encryption.

The investigator and study staff are responsible for maintaining a comprehensive and centralized filing system of all study-related (essential) documentation, suitable for inspection at any time by representatives of the sponsor and/or applicable regulatory authorities. Elements should include:

- Subject files containing completed eCRFs, informed consents, and supporting copies of source documentation
- Study files containing the protocol with all amendments, investigator’s brochure, copies of prestudy documentation (see section 12.3), and all correspondence to and from the EC.

In addition, all original source documents supporting entries in the eCRFs must be maintained and be readily available.

No study document should be destroyed without prior written agreement between the Steering Committee (or its appointed person) and the investigator.

13.3 Study Monitoring and Data Collection:

The Steering Committee representative and regulatory authority inspectors are responsible for contacting and visiting the investigator for the purpose of inspecting the facilities and, upon request, inspecting the various records of the trial (e.g., eCRFs and other pertinent data) provided that subject confidentiality is respected. The monitor appointed by the Steering Committee is responsible for inspecting the eCRFs at regular intervals as specified in the study monitoring plan, throughout the study to verify adherence to the protocol; completeness, accuracy, and consistency of the data; and adherence to local regulations on the conduct of clinical research. The monitor should have access to subject medical records and other study-related records needed to verify the entries on the eCRFs.

The study is completed in accordance with this protocol and ICH-GCP guidelines. The investigator agrees to cooperate with the monitor to ensure that any problems detected in the course of these monitoring visits, including delays in completing eCRFs, are resolved. Inspection of site facilities and review of study-related records may occur to evaluate the trial conduct and compliance with the protocol, ICH-GCP, and applicable regulatory requirements.

All data should be entered into the web based CRF. The web based CRF should be completed when logged on to the system in the investigators own name.

Corrections in the electronic CRF are made electronically. The change must be initialised and dated by the investigator or a member of the study staff authorized by the investigator. Corrections to electronic forms will be automatically documented through the software's "audit trail".

To ensure the quality of clinical data across all subjects and sites, a clinical data management review will be performed on subject data received at the study office. During this review, subject data will be checked for consistency, omissions, and any apparent discrepancies. In addition, the data will be reviewed for adherence to the protocol and GCP. To resolve any questions arising from the clinical data management review process, data queries and/or site notifications will be sent to the site for completion and return to the study office.

The principal investigator will sign and date the indicated places on the eCRF. These signatures will indicate that the principal investigator inspected or reviewed the data on the case report form, the data queries, and the site notifications, and agrees with the content.

The study office will correct the database for the following CRF issues without notification to site staff:

- Misspellings, that do not change the meaning of the word (excluding AEs and medications)
- Location of data recorded on an incorrect CRF (e.g., moving lab data from general comments to the appropriate lab table)
- Obvious date errors
- Standard time to 24-hour clock errors
- Weight unit errors if a baseline weight has been established
- Height unit errors
- Administrative data (eg, event names for unscheduled visits or retests)
- Clarifying "other, specify" if data are provided (eg, race, physical exam)
- If both the end date and a status of continuing is indicated (eg, for AEs, concomitant medication, hospitalization) the end date will supersede
- Deletion of obvious duplicate data (eg, same results sent twice with the same date but different clinical planned events – week 4 and early termination)
- For AEs that record action taken code = 01 (none) and any other action code, 01 (none) may be deleted as it is superseded by other existing data
- If equivalent units or terms are recorded instead of the acceptable the sponsor standard (e.g., cc for mL, SQ for SC route, Not Examined for Not Done), the sponsor units or terms will be used
- If the answer to a YES or NO question is blank or obviously incorrect (e.g., Answers to the following questions do not reflect the data that are recorded or missing: Were there any AEs? Concomitant medications? Hospitalizations?)

All data will be kept for 15 years.

13.4 Language:

CRFs will be in English. Registered trade names for concomitant medications may be used instead of generic names. All written information and other material to be used by subjects and investigative staff must use vocabulary and language that are clearly understood.

13.5 Publication Policy:

To coordinate dissemination of data from this study, a publication committee consisting of the SC and selected participants is formed. The committee is expected to solicit input and assistance from other investigators as appropriate. Membership of the committee does not guarantee authorship—the criteria described below should be met for every publication. Authorship of any publications resulting from this study will be determined on the basis of the Uniform Requirement for Manuscripts Submitted to Biomedical Journals (International Committee of Medical Journal Editors, 1997) which states:

Each author should have participated sufficiently in the work to take public responsibility for the content. Authorship credit should be based on only substantial contributions to:

(a) Conception and design, or analysis and interpretation of data.

(b) Drafting the article or revising it critically for important intellectual content.

(c) Final approval of the version to be published.

Conditions (a), (b), and (c) must all be met.

All publications (e.g., manuscripts, abstracts, oral/slide presentations, book chapters) based on this study must be submitted to the Steering Committee for review.

The results from the study - both positive and negative as well as inconclusive results– will be submitted for publication in international peer-reviewed scientific journals.

13.6 Compensation:

Subjects will be covered by normal patient insurance.

14. REFERENCES:

1. Koch MB, Davidsen M, Juel K.
   Heart Diseases in Denmark: Incidence and future development. Report from the Danish Heart Foundation. October 2011.

http://www.hjerteforeningen.dk/files/Rapporter_mm/Hjertekarsygdomme%20i%20Danmark.%20Forekomst%20og%20udvikling%202000-2009.pdf

2. Kirchhof P, Adamou A, Knight E, et al.
How can we avoid a stroke crisis? ESC Working group report. Stroke prevention in atrial fibrillation. December 2009.
http://www.escardio.org/communities/EHRA/publications/papers-interest/Documents/ehra-stroke-report-recommend-document.pdf

3. Camm AJ, Kirchhof P, Lip GY et al.
Guidelines for the management of atrial fibrillation: The Task Force for the management of atrial fibrillation of the European Society of Cardiology.
Europace. 2010; 12: 1360-1420.

4. Healey JS, Connolly SJ, Gold MR et al.
Subclinical atrial fibrillation and the risk of stroke.
NEJM 2012; 366: 120-9.

5. Savelieva I, Kakouros N, Kourliouros A, and Camm AJ.
Upstream therapies for management of atrial fibrillation: review of clinical evidence and implications for European Society of Cardiology guidelines. Part I: primary prevention.
Europace 2011; 13: 308-328.

6. De Jong AM, Maass AH, Oberdorf-Maass SU, Van Veldhuisen DJ, Van Gilst WH, Van Gelder IC. Mechanisms of atrial structural changes caused by stretch occurring before and during early atrial fibrillation.
Cardiovasc Res 2010; 89: 754-765.

7. Nattel S, Burstein B and Dobrev D.
Atrial Remodeling and Atrial Fibrillation: Mechanisms and Implications.
Circ Arrhythmia Electrophysiol 2008; 1: 62-73.

8. Cheng S, Keyes MJ, Larson MG et al.
Long-term outcomes in individuals with prolonged PR interval or first-degree atrioventricular block. JAMA 2009; 301: 2571-7.

9. Kolb C, Nürnberger S, Ndrepepa G et al.
Modes of initiation of paroxysmal atrial fibrillation from analysis of spontaneously occurring episodes using a 12-lead Holter monitoring system.
Am J Cardiol 2001; 88: 853-7.

10. Porsdal V, Boysen G.
Direct costs during the first year after intracerebral hemorrhage.
Eur J Neurol 1999; 6: 449-54.

11. Jørgensen HS, Nakayama H, Raaschou HO et al.
Acute stroke care and rehabilitation: an analysis of the direct cost and its clinical and social determinants. The Copenhagen Stroke Study.
Stroke 1997; 28: 1138-41.

12. Lunde L.
Can EQ-5D and 15D be used interchangeably in economic evaluations? Assessing quality of

life in post-stroke patients.
Eur J Health Econ 2012 Jun 8. Epub ahead of print.

13. Larsen T, Olsen TS, Sorensen J.
Early home-supported discharge of stroke patients: A health technology assessment.
Int J Technol Assess Health Care 2006; 22: 313-20.

14. Pink J, Lane S, Pirmohamed M, Hughes DA.
Dabigatran etexialte versus warfarin in management of non-valvular atrial fibrillation in UK context: quantitative benefit-harm and economic analyses.
BMJ 2011 Oct 31;343:d6333.

15. Sorensen SV, Kansal AR, Connolly S et al.
Cost-effectiveness of dabigatran etexilate for the prevention of stroke and systemic embolism in atrial fibrillation: a Canadian payer perspective.
Thromb Haemost 2011; 105: 908-19.

16. You JH, Tsui KK, Wong RS, Cheng G.
Cost-Effectiveness of Dabigatran versus Genotype-Guided Management of Warfarin Therapy for Stroke Prevention in Patients with Atrial Fibrillation.
PLoS One 2012;7:e39640.

17. The CONSORT Group. http://www.consort-statement.org

18. Truelsen T, Piechowski-Jóźwiak B, Bonita R, Mathers C, Bogousslavsky J, Boysen G.
Stroke incidence and prevalence in Europe: a review of available data.
Eur J Neurol. 2006; 13: 581-98.

19. Knopman DS, Pennman AD, Catellier DJ, Coker LH et al.
Vascular risk factors and longitudinal changes on brain MRI - The ARIC study.
Neurology 2011; 76: 1879–1885,

20. Rostrup E, Gouw AA, Vrenken H, van Straaten ECW, Ropele S, Pantoni L, Inzitari D, Barkhof F, Waldemar G on behalf of the LADIS study group.
The spatial distribution of age-related white matter changes as a function of vascular risk factors— Results from the LADIS study.
NeuroImage 2012; 60: 1597-1607.

21. Pedersen KM.
DRG again again.
Ugeskr Laeger 2010; 172:2205.

22. Wittrup-Jensen KU, Lauridsen J, Gudex C et al.
Generation of a Danish TTO value set for EQ-5D health states.
Scand J Public Health 2009; 37: 459-66.

Supplementary references on the scientific themes:

Ahlsson A, Fengsrud E, Bodin L, Englund A.
Postoperative atrial fibrillation in patients undergoing aortocoronary bypass surgery carries an eightfold risk of future atrial fibrillation and a doubled cardiovascular mortality.
Eur J Cardiothorac Surg (2010), doi:10.1016/j.ejcts.2009.12.033

Botto GL, Padeletti L, Santini M, et al.
Presence and Duration of Atrial Fibrillation Detected by Continuous Monitoring: Crucial Implications for the Risk of Thromboembolic Events.
J Cardiovasc Electrophysiol, Vol. pp. 1-8.

Connolly SJ, Ezekowitz MD, Yusuf S et al.
Dabigatran versus Warfarin in Patients with Atrial Fibrillation.
N Engl J Med 2009;361:1139-51.

Hanke T, Charitos EI, Stierle U, et al.
Twenty-Four–Hour Holter Monitor Follow-Up Does Not Provide Accurate Heart Rhythm Status After Surgical Atrial Fibrillation Ablation Therapy. Up to 12 Months Experience With a Novel Permanently Implantable Heart Rhythm Monitor Device.
Circulation 2009;120[suppl 1]:S177–S184.

Huikuri H, Raatikainen MJP, Moerch-Joergens R, et al.
Prediction of fatal or near-fatal cardiac arrhythmia events in patients with depressed left ventricular function after an acute myocardial infarction.
European Heart Journal (2009) 30, 689–698. doi:10.1093/eurheartj/ehn537

Pedersen OD, Bagger H, Keller N, et al.
Efficacy of Dofetilide in the Treatment of Atrial Fibrillation-Flutter in Patients With Reduced Left Ventricular Function A Danish Investigations of Arrhythmia and Mortality ON Dofetilide (DIAMOND) Substudy.
Circulation 2001;104;292-296.

Sinha AM, Diener HC, Morillo CA, et al.
Cryptogenic Stroke and underlying Atrial Fibrillation (CRYSTAL AF): Design and rationale.
Am Heart J 2010;160:36-41.e1.

Pokushalov E, Romanov A, Corbucci G, et al.
Use of An Implantable Monitor to Detect Arrhythmia Recurrences and Select Patients for Early Repeat Catheter Ablation for Atrial Fibrillation: A Pilot Study.
Circ Arrhythm Electrophysiol. 2011 Dec;4(6):823-31. doi: 10.1161/CIRCEP.111.964809. Epub 2011 Sep 19.

Lamas G.
How much atrial fibrillation is too much atrial fibrillation?
N Engl J Med 2012 Jan 12;366(2):178-80. doi: 10.1056/NEJMe1111948.

Bloch Thomsen PE, Jons C, Raatikainen MJ, Moerch Joergensen R, et al.
Long-term recording of cardiac arrhythmias with an implantable cardiac monitor in patients with reduced ejection fraction after acute myocardial infarction: the Cardiac Arrhythmias and Risk Stratification After Acute Myocardial Infarction (CARISMA) study.
Circulation. 2010 Sep 28;122(13):1258-64. doi: 10.1161/CIRCULATIONAHA.109.902148. Epub 2010 Sep 13.

Buxton AE.
Implantable loop recorder in survivors of acute myocardial infarction: a glimpse of reality?
Circulation. 2010 Sep 28;122(13):1255-7. doi: 10.1161/CIRCULATIONAHA.110.976365. Epub 2010 Sep 13.

Heeringa J, van der Kuip DA, Hofman A, et al.
Prevalence, incidence and lifetime risk of atrial fibrillation: the Rotterdam study.
Eur Heart J. 2006 Apr;27(8):949-53. Epub 2006 Mar 9.

Brignole M, Vardas P, Hoffman E, et al.
Indications for the use of diagnostic implantable and external ECG loop recorders.
Europace. 2009 May;11(5):671-87. doi: 10.1093/europace/eup097.

Pedersen OD, Bagger H, Køber L, Torp-Pedersen C; TRACE Study Group.
Impact of congestive heart failure and left ventricular systolic function on the prognostic significance of atrial fibrillation and atrial flutter following acute myocardial infarction.
Int J Cardiol. 2005 Apr 8;100(1):65-71.

van Dam PM, van Oosterom A.
Analysing the potential of Reveal for monitoring cardiac potentials.
Europace. 2007 Nov;9 Suppl 6:vi119-23.

Pokushalov E, Taborsky M, Hindricks G., et al.
Variables Influencing Sensing after Reveal ImplanTation (VISIT).
Abstract presented at EURPACE 2009, 21 June - 24 June 2009, Berlin, Germany.

Friberg L, Hammar N, Pettersson H, Rosenqvist M.
Increased mortality in paroxysmal atrial fibrillation: report from the Stockholm Cohort-Study of Atrial Fibrillation (SCAF).
Eur Heart J. 2007 Oct;28(19):2346-53. Epub 2007 Aug 1.

Heeringa J, Conway DS, van der Kuip DA.
A longitudinal population-based study of prothrombotic factors in elderly subjects with atrial fibrillation: the Rotterdam Study 1990-1999.
J Thromb Haemost. 2006 Sep;4(9):1944-9. Epub 2006 Jul 5.

Petkar S, Hamid T, Iddon P.
Prolonged implantable electrocardiographic monitoring indicates a high rate of misdiagnosis of epilepsy--REVISE study.
Europace. 2012 Nov;14(11):1653-60. doi: 10.1093/europace/eus185. Epub 2012 Jun 28.

Zimetbaum P, Goldman A.
Ambulatory arrhythmia monitoring: choosing the right device.
Circulation. 2010 Oct 19;122(16):1629-36. doi: 10.1161/CIRCULATIONAHA.109.925610

Binici Z, Intzilakis T, Nielsen OW.
Excessive supraventricular ectopic activity and increased risk of atrial fibrillation and stroke.
Circulation. 2010 May 4;121(17):1904-11. doi: 10.1161/CIRCULATIONAHA.109.874982. Epub 2010 Apr 19.

Ravensbergen et al Regarding Article, “Long-Term Recording of Cardiac Arrhythmias With an Implantable Cardiac Monitor in Patients With Reduced Ejection Fraction After Acute Myocardial Infarction: The Cardiac Arrhythmias and Risk Stratification After Acute Myocardial Infarction (CARISMA) Study” (Letter).
Circulation. 2012 Jan 3;125(1):e239

Friberg L, Hammar N, Rosenqvist M.
Stroke in paroxysmal atrial fibrillation: report from the Stockholm Cohort of Atrial Fibrillation.

Eur Heart J. 2010 Apr;31(8):967-75. doi: 10.1093/eurheartj/ehn599. Epub 2009 Jan 27.

Henriksson KM, Farahmand B, Asberg S, et al.
First-ever atrial fibrillation documented after hemorrhagic or ischemic stroke: the role of the CHADS(2) score at the time of stroke.
Clin Cardiol. 2011 May;34(5):309-16. doi: 10.1002/clc.20869. Epub 2011 Mar 13.

Kolominsky-Rabas PL, Heuschmann PU, Marschall D, et al.
Lifetime cost of ischemic stroke in Germany: results and national projections from a population-based stroke registry: the Erlangen Stroke Project.
Stroke 2006 May;37(5):1179-83. Epub 2006 Mar 30.

Edvardsson N, Frykman V, van Mechelen R, et al.
Use of an implantable loop recorder to increase the diagnostic yield in unexplained syncope: results from the PICTURE registry.
Europace. 2011 Feb;13(2):262-9. doi: 10.1093/europace/euq418. Epub 2010 Nov 19

Winkel TA, Rouwet EV, van Kuijk JP, et al.
Aortic surgery complications evaluated by an implanted continuous electrocardiography device: a case report.
Eur J Vasc Endovasc Surg. 2011 Mar;41(3):334-6. doi: 10.1016/j.ejvs.2010.11.006. Epub 2010 Dec 31.

Pokushalov E, Romanov A, Corbucci G, et al.
Ablation of paroxysmal and persistent atrial fibrillation: 1-year follow-up through continuous subcutaneous monitoring.
J Cardiovasc Electrophysiol. 2011 Apr;22(4):369-75. doi: 10.1111/j.1540-8167.2010.01923.x. Epub 2010 Oct 11.

Jons C, Jacobsen UG, Joergensen RM, et al.
The incidence and prognostic significance of new-onset atrial fibrillation in patients with acute myocardial infarction and left ventricular systolic dysfunction: a CARISMA substudy.
Heart Rhythm. 2011 Mar;8(3):342-8. doi: 10.1016/j.hrthm.2010.09.090. Epub 2010 Nov 18

Goetze JP, Friis-Hansen L, Rehfeld JF.
Atrial secretion of B-type natriuretic peptide.
Eur Heart J. 2006 Jul;27(14):1648-50. Epub 2006 Jun 19.

Hindricks G, Pokushalov E, Urban L, et al.
Performance of a new leadless implantable cardiac monitor in detecting and quantifying atrial fibrillation: Results of the XPECT trial.
Circ Arrhythm Electrophysiol. 2010 Apr;3(2):141-7. doi: 10.1161/CIRCEP.109.877852. Epub 2010 Feb 16.

El-Chami MF, Kilgo P, Thourani V, et al.
New-onset atrial fibrillation predicts long-term mortality after coronary artery bypass graft.
J Am Coll Cardiol. 2010 Mar 30;55(13):1370-6. doi: 10.1016/j.jacc.2009.10.058.

Pokushalov E, Romanov A, Cherniavsky A, et al.
Ablation of paroxysmal atrial fibrillation during coronary artery bypass grafting: 12 months' follow-up through implantable loop recorder.
Eur J Cardiothorac Surg. 2011 Aug;40(2):405-11. doi: 10.1016/j.ejcts.2010.11.083. Epub 2011 May

23.

Kirchhof P, Lip GY, Van Gelder IC, et al.
Comprehensive risk reduction in patients with atrial fibrillation: emerging diagnostic and therapeutic options—a report from the 3rd Atrial Fibrillation Competence NETwork/European Heart Rhythm Association consensus conference.
Europace. 2012;14(1):8-27

Fang MC, Go AS, Chang Y, et al.
Comparison of risk stratification schemes to predict thromboembolism in people with non-valvular atrial fibrillation.
J Am Coll Cardiol 2008;51(8):810-815

Camm AJ, Corbucci G, Padeletti L.
Usefulness of Continuous Electrocardiographic Monitoring for Atrial Fibrillation.
Am J Cardiol 2012; 110(2):270-276

Glotzer TV, Hellkamp AS, Zimmerman J, et al.
Atrial high rate episodes detected by pacemaker diagnostics predicts death and stroke.
Circulation 2003;107(12):1614-1619

Capucci A, Santini M, Padeletti L, et al.
Monitored atrial fibrillation duration predicts arterial embolic events in patients suffering from bradycardia and atrial fibrillation implanted with antitachycardia pacemakers.
J Am Coll Cardiol 2005; 46(10):1913–1920

Glotzer TV, Daoud EG, Wyse DG, et al.
The Relationship between Daily Atrial Tachyarrhythmia Burden From Implantable Device Diagnostics and Stroke Risk. The TRENDS Study.
Circ Arrhythmia Electrophysiol 2009;2(5):474-480

Boriani G, Botto GL, Padeletti L, et al.
Improving stroke risk stratification using the CHADS2 and CHA2DS2VASc risk scores in paroxysmal atrial fibrillation patients by continuous arrhythmia burden monitoring.
Stroke 2011;42(6):1768-1770

Santini M, Gasparini M, Landolina M, et al.
Device-detected atrial tachyarrhythmias predict adverse outcome in real-world patients with implantable biventricular defibrillators.
J Am Coll Cardiol 2011. 57(2):167-172

Welles CC, Whooley MA, Na B, Ganz P, Schiller NB, Turakhia MP.
The CHADS2 score predicts ischemic stroke in the absence of atrial fibrillation among subjects with coronary heart disease: Data from the Heart and Soul Study,
Am Heart J. 2011;162(3):555-561

Purerfellner H, Gillis AM, Holbrook R, Hettrick DA.
Accuracy of atrial tachyarrhythmia detection in implantable devices with arrhythmia therapies.
Pacing Clin Electrophysiol. 2004; 27(7): 983–992

Lamas G.
How Much Atrial Fibrillation Is Too Much Atrial Fibrillation?
N Engl J Med 2012; 366(2):178-180

Boriani G., Santini M., Lunati M, .et al.
Improving Thromboprophylaxis Using Atrial Fibrillation Diagnostic Capabilities in Implantable Cardioverter-Defibrillators: The Multicentre Italian ANGELS of AF Project,
Circ Cardiovasc Qual Outcomes 2012; 5(2):182-188

van Elderen SG, de Roos A, de Craen AJ, et al.
Progression of brain atrophy and cognitive decline in diabetes mellitus: a 3-year follow-up.
Neurology. 2010 Sep 14;75(11):997-1002. doi: 10.1212/WNL.0b013e3181f25f06.

Knopman DS, Penman AD, Catellier DJ, et al.
Vascular risk factors and longitudinal changes on brain MRI: the ARIC study.
Neurology. 2011 May 31;76(22):1879-85. doi: 10.1212/WNL.0b013e31821d753f. Epub 2011 May 4.

Hajjar I, Quach L, Yang F, et al.
Hypertension, white matter hyperintensities, and concurrent impairments in mobility, cognition, and mood: the Cardiovascular Health Study.
Circulation. 2011 Mar 1;123(8):858-65. doi: 10.1161/CIRCULATIONAHA.110.978114. Epub 2011 Feb 14.

Portet F, Brickman AM, Stern Y, et al.
Metabolic syndrome and localization of white matter hyperintensities in the elderly population.
Alzheimers Dement. 2012 Oct;8(5 Suppl):S88-95.e1. doi: 10.1016/j.jalz.2011.11.007. Epub 2012 Jun 6.

Raz N, Yang Y, Dahle CL, et al.
Volume of white matter hyperintensities in healthy adults: contribution of age, vascular risk factors, and inflammation-related genetic variants.
Biochim Biophys Acta. 2012 Mar;1822(3):361-9. doi: 10.1016/j.bbadis.2011.08.007. Epub 2011 Aug 25.

Rostrup E, Gouw AA, Vrenken H, et al.
The spatial distribution of age-related white matter changes as a function of vascular risk factors--results from the LADIS study.
Neuroimage. 2012 Apr 15;60(3):1597-607. doi: 10.1016/j.neuroimage.2012.01.106. Epub 2012 Jan 28.

Seo SW, Lee JM, Im K, et al.
Cardiovascular risk factors cause cortical thinning in cognitively impaired patients: relationships among cardiovascular risk factors, white matter hyperintensities, and cortical atrophy.
Alzheimer Dis Assoc Disord. 2012 Apr-Jun;26(2):106-12. doi: 10.1097/WAD.0b013e31822e0831.

Gorelick PB, Scuteri A, Black SE, et al.
Vascular contributions to cognitive impairment and dementia: a statement for healthcare professionals from the american heart association/american stroke association.
Stroke. 2011 Sep;42(9):2672-713. doi: 10.1161/STR.0b013e3182299496. Epub 2011 Jul 21.

Zhu YC, Chabriat H, Godin O, et al.
Distribution of white matter hyperintensity in cerebral hemorrhage and healthy aging.
J Neurol. 2012 Mar;259(3):530-6. doi: 10.1007/s00415-011-6218-3. Epub 2011 Aug 30.

15. APPENDICES:

Appendix A – Study procedures – summary
Appendix B – Composition of Committees
Appendix C – Serious Adverse Events
Appendix D – Blood Samples for Genetic analysis and Blood Samples for Biomarkers
Appendix E – ILR information
Appendix F – Definition of composite endpoint
Appendix G – Funding
Appendix H – Informed consent
Appendix I – Guidelines for oral information

Appendix A – Study procedures – Summary
Study flow chart – a) ILR group

Phase	Screen	Rand.	Impl.	Follow-up						
**Visit**	**1**	**2**	**3**	**3A**	**4**	**4A**	**5**	**5A**	**6**	**6A**
**Month**	0,5	0	0,5		12,5	13-24	24,5	25-36	36,5	37-48
**Day**	-14	0	14		374		734		1094	
**Informed consent**	√									
**Medical history**	√				√		√		√	
**Concomitant Disease**	√				√		√		√	
**Concomitant Medication**	√				√		√		√	
**Demographics**	√									
**Echocardiography**		√					(√)		(√)	
**12-lead ECG**	√				√		√		√	
**Randomization**		√								
**Vital signs**	√				√		√		√	
**QoL and cognitive function**	√				√		√		√	
**Blood sample**	√									
**Adverse events**					√		√		√	
**MRI**		(√)		(√)		(√)				
**Health economics**					√		√		√	
**Remote ILR interrogation #**			√	√	√	√	√	√	√	√

# : only relevant for the ILR group; remote transmissions are sent automatically on a daily basis and routinely as patient activated every 4 weeks (to obtain ECG data during presumed sinus rhythm) during the entire study period

Appendix A – Study procedures – Summary
Study flow chart – b) Control group

Phase	Screen	Rand.	Impl.	Follow-up						
**Visit**	**1**	**2**	**3**	**3A**	**4**	**4A**	**5**	**5A**	**6**	**6A**
**Month**	0,5	0	0,5		12,5	13-24	24,5	25-36	36,5	37-48
**Day**	-14	0	14		374		734		1094	
**Informed consent**	√									
**Medical history**	√				√		√		√	
**Concomittant. Disease**	√				√		√		√	
**Concomittant Medication**	√				√		√		√	
**Demographics**	√									
**Echocardiography**		√					(√)		(√)	
**12-lead ECG**	√								√	
**Randomization**		√								
**Vital signs**	√								√	
**QoL and cognitive function**	√				√		√		√	
**Blood sample**	√									
**Adverse events**					√		√		√	
**MRI**		(√)		(√)		(√)				
**Health economics**					√		√		√	
**Remote ILR interrogation #**										

# : only relevant for the ILR group; remote transmissions are sent automatically on a daily basis and routinely as patient activated every 4 weeks (to obtain ECG data during presumed sinus rhythm) during the entire study period

Appendix B – Composition of Committees

Composition of the Steering committee (SC):

The SC, which consists of representatives from the founding partner institutions, will provide advice as to the project´s overall scientific quality and budget as well as concerning the balance between the project´s activities, goals and results.

The members of the Steering committee are:
Jesper Hastrup Svendsen, Rigshospitalet (chairman)
Lars Køber, Rigshospitalet
Søren Højberg, Bispebjerg Hospital
Ketil Haugan, Roskilde Hospital
Axel Brandes, Odense University Hospital
Christian Kronborg, University of Southern Denmark
Claus Graff, Aalborg University
Derk W. Krieger, Bispebjerg Hospital

Composition of the Event Committee (EC):

The event committee is responsible for evaluating and adjudicating events comprising all possible primary endpoints. The EC will classify deaths according to pre-specified criteria modified according to Hinkle and Thaler. Deaths will be classified as cardiovascular and non-cardiovascular. Cardiovascular will be divided into sudden and non-sudden cardiovascular deaths based on time specifications. Strokes will be divided into ischaemic and non-ischaemic strokes (bleeds) and non-classifiable strokes. The guidelines for classifications will be described in a separate charter. There will be a neurologist and a cardiologist in the EC. The members of the EC are:

- Neurologist Professor Derk W. Krieger
- Cardiologist Professor Lars Køber

Composition of the International Advisory Committee (IAC):

An *International Scientific Advisory Group*, which will meet annually, will discuss issues of relevance for the project (theory, methods and results) and consist of internationally recognised experts in the field. The members of the IAC are:

- Professor Gregory Y.H. Lip, University of Liverpool, UK;

- Professor Mårten Rosenqvist, Karolinska Institute, Sweden; and
- Professor Dan Atar, University of Oslo, Norway.

They have all agreed to serve in the advisory group. All three are senior cardiologists with a strong scientific track record in atrial fibrillation.

Appendix C – Serious Adverse Events

Serious adverse events (SAE) are reported immediately in the electronic eCRFs. Every 6 month a summary report is generated to the Steering Committee. This report contains information on all SAEs. Since the study is performed in a study population with an increased risk of stroke and mortality SAE summaries will not be reported on a daily basis. An annual report will also be forwarded to the Ethics committee of the Capitol Region of Denmark. This report will be accompanied by a description of the events together with a statement regarding eventual consequences for the study.

Appendix D – Blood Samples for Genetic analysis and Blood Samples for Biomarkers

On inclusion, blood samples will be taken for routine measurement of electrolytes (Na and K), creatinine, haemoglobin concentration, white blood cells, platelets, troponins, pro-BNP and CRP (a total of 15 ml venous blood).

In addition, a total of 32 ml of venous blood is collected and stored in a research biobank.

The sampled blood for the research biobank is collected into a total of 6 vials:

A part of the blood is collected in 3 x 6 ml vials (purple coloured, K2EDTA) and stored as full-blood (a part is stored in vials containing RNAlater).

In addition, one vial containing 6 ml blood (purple coloured, K2EDTA) and 2 vials containing 4 ml blood (Z serum clot activator) are collected for centrifuging and after pipetting the supernatant is stored as plasma and serum, respectively.

Centrifuging is performed within 30 minutes after venous puncture and carried out at 3,000 RPM for 10 minutes. Samples are stored in freezer at minus 80 degrees Celcius.

Samples will be transferred to Rigshospitalet where they will be stored in the study related research biobank in a locked freezer. Surplus biological material from the research biobank is stored in a biobank for future research.

Appendix E – Device information: the Medtronic Reveal® LINQ:

In this study the Medtronic Reveal® LINQ insertable, cardiac monitor is used. The Reveal® LINQ is the newest and smallest ILR on the market which is expected to be market released by February 2014. It has battery longevity of three years and additionally 12 months of shelf life. Its size is 1.19 cc which is about 10% of the previous model (Medtronic Reveal® LINQ which has a volume of 9 cc). In contrast to the XT model the LINQ model has a build-in antenna which enables automatic remote transmissions. The device has an event memory of a total of 57 minutes (which is longer than the memory of the XT model: 49.5 minutes) (128 Hz) with up to 30 events in total. The total memory capacity is divided into 22.5 minutes of patient activated events (with the three most recent manual events lasting 7.5 minutes each), and additionally 27 minutes auto-activated events. The device is magnetic resonance (MR) conditional, which means that the Reveal® is safe for use in MR environments. Due to the algorithm the device uses for AF detection it will only categorize an arrhythmia event as atrial fibrillation, if it lasts more than two minutes in total. The arrhythmia detection capabilities of the Reveal® LINQ categorize arrhythmias into 5 groups: Fast VT, VT, bradycardia, asystolia, and AT/AF. The Reveal® LINQ Recorder is prepared for CareLink remote monitoring, with both automated daily transmissions and patient activated transmissions. The device can record AT/AF burden, heart rate variability, day versus night frequency and patient activity. Prior to implant it is recommended to check the vector of the device, with the purpose of finding the orientation with optimal R wave amplitude via the programmer. The device is supplied with a so-called patient assistant, which is a patient activating device. The device algorithm for atrial fibrillation detection is based on measurement of the RR-intervals and the delta RR-interval (change in RR interval from the preceding RR interval), which is afterwards visually presented in a so-called Lorenz plot (cluster signature matrix), where normal sinus rhythm or atrial flutter will be shown as a highly concentrated black spot in the center of the plot, whereas atrial fibrillation will be widely distributed in the cluster signature matrix (”gunshot profile”).

When patient activated episodes are stored, there is a total of 22.5 minutes for the episodes, and the device will always store the three last events, each lasting 7.5 minutes. Each of these 7.5 minute events will comprise 6.5 minutes prior to patient activation as well as 1 minute after patient activation. After patient activation a new episode will only be stored, if it happens more than 5 minutes after last activation. If the memory is full, the oldest episode will be overwritten. Per episode type there is a guaranteed storage of the three most resent episodes. When memory is full, the oldest stored ECGs will be removed. If data have been transmitted in the form of ECGs these will be stored forever as ECGs on Carelink. All episodes recorded by the ILR will be stored (as episodes with interpretation markers, but not necessarily as ECGs).

The system offers a visual indication to provide the patient with information that either everything is fine (no arrhythmia has been detected) (green lamp) or something with the heart rhythm needs action – please contact hospital (red lamp).
In the LOOP study it may seem reasonable to turn off one of the VT zones to increase the data storage.

In patients with frequent premature supraventricular contractions which the device incorrectly classify as atrial fibrillation, the device can be programmed to ignore these episodes by programming the function “ectopy rejection” “on” which will cause these episodes to be categorised as sinus rhythm.
It is possible to bring the Carelink box to another country and make transmissions from abroad.

Suggested device programming of parameters:

h) FVT parameters are set at: 30/40 and a rate >222 bpm
i) VT parameters are set at: 24 consequtive beats at a rate > 182 bpm
j) Bradycardia is set at a rate < 30 bpm
k) Asystolia is set at a duration > 4.5 sec
l) Symptom activation is programmed on and programmed to 3 episodes of 7.5 min each
m) AT/AF is programmed at AF only on
n) Ectopy rejection is programmed off in standard settings (only programmed on in case it is observed that the device falsely categorises premature contractions as AF.

Since the LINQ Reveal has not yet been market released we do not fully know the ideal programming yet.

When the ILR reaches end of battery life the device is explanted. If a patient dies with an implanted ILR the device is explanted by a post-mortem procedure.

Appendix F – Definition of the composite endpoint

1: Stroke is defined as rapid onset of a focal/global neurological deficit, with duration ≥ 24 hours, or resulting in death, or supported by clear evidence of cerebral infarction on diffusion-weighted MRI imaging. There must be no other readily identifiable non-stroke cause for the clinical presentation.

Stroke is sub- categorized as follows:

-1a: Ischemic stroke, defined as stroke caused by lack of blood supply (e.g. thrombosis, embolism, systemic hypoperfusion or cerebral venous sinus thrombosis)

-1b: Hemorrhagic stroke, defined as a stroke caused by cerebral or subarachnoid hemorrhage.

-1c: Unspecified stroke, in case the patient did not undergo brain imaging or autopsy

2: Systemic arterial embolism include arterial embolic events that do not reach the brain; these may reach organs such as kidney, spleen, heart, intestine, eyes and extremities.

Adjudication is based on clinical evaluation of patient data.

Transient ischemic attack (TIA) is not a primary event, and is defined as a transient episode of neurologic dysfunction caused by focal brain, spinal cord, or retinal ischemia, **without** acute infarction.

Explanation: TIA was originally defined clinically by the temporary nature (<24 hours) of the associated neurologic symptoms. However, the arbitrary nature of the 24-hour time limit and lack of specific pathophysiologic meaning hampered the clinical and research utility of the term "TIA." Recognition of these problems led to a change to a tissue-based definition of TIA. The change was driven by advances in neuroimaging that enabled very early identification of ischemic brain injury and endorsed by 2009 guidelines from the American Heart Association and American Stroke Association (AHA/ASA).

Appendix G –Funding

Research grants have been obtained from the Danish National Foundation for Strategic Research (DKK 15.6 mio), the Research Foundation for the Capital Region of Denmark (DKK 2.0 mio) and the Danish Heart Foundation (DKK 150.000).
The first 600 implantable loop recorders(ILRs) used in the project will be purchased from the study funding from the Danish National Foundation for Strategic Research. The condition of this purchase has been altered so that the Reveal LINQ´s will be offered on the same terms as the original Reveal XT devices. The additional 900 Reveal LINQ ILRs will be donated from Medtronic as an unrestricted research grant (which has a value of DKK 5.4 mio (reduced price) or DKK 22.5 mio (ordinary list price)) and a grant to cover salary for research nurses at the hospitals (DKK 1.9 mio).
Medtronic has decided to support the LOOP study with an unrestricted research grant covering salaries for one research nurse for one year at each of the four participating hospitals (i.e. a total of four years of salary for research nurses; estimated salary per nurse including pension and vacation is 40,000 DKK per month which equals 480,000 DKK per year/nurse; i.e. a total of 1.920.000 DKK (the same salary estimate was used in the application to the Strategic Research Council).
Additional research grants will be applied for from private and public research foundations as well as from industry. Study participants and the Ethics committee will be informed about additional funding.
Patients will not receive compensation for their participation in the study, but may in selected cases receive compensation for their transport expenses.

Appendix H – Informed consent

SAMTYKKE ERKLÆRING OG FULDMAGT VDR. LOOP FORSØGET

- ✓ Jeg har modtaget og læst deltagerinformationen for dette forsøg, og min læge har besvaret alle mine spørgsmål vedrørende det videnskabelige forsøg.
- ✓ Jeg har haft tilstrækkelig tid til at overveje min deltagelse i forsøget, og jeg er opmærksom på at deltagelse i dette forsøg sker helt frivilligt.
- ✓ Jeg er klar over, at jeg kan afslutte min deltagelse på et hvilket som helst tidspunkt, uden at det påvirker kvaliteten af sundhedsvæsenets ydelser til mig.
- ✓ Jeg forstår, og er enig i, at mine personlige oplysninger bliver indsamlet fra mine medicinske journaler, brugt og behandlet (manuelt og computermæssigt) af de læger som er ansvarlige for undersøgelsen, eller alle andre udpegede, som indgår i dette forsøg (dvs. hospitaler, læger, lovgivende myndigheder, etiske komitéer).
- ✓ Jeg har modtaget en kopi af patientinformationen og denne samtykkeerklæring.

Vedrørende forskningsbiobank:
☐ Jeg giver tilladelse til at min blodprøve, må udtages og opbevares i en forskningsbiobank. Det biologiske materiale vil ikke uden fornyet godkendelse hos De Videnskabsetiske komiteer blive brugt i andre forskningsprojekter eller til andre formål og analyser, end dem, der er nævnt i informationen.

Du bedes tilkendegive om du ønsker din egen læge informeret om din deltagelse i forsøget:
☐ *Jeg ønsker min praktiserende læge skal informeres om min deltagelse i dette forsøg*
☐ *Jeg ønsker <u>ikke</u> min praktiserende læge skal informeres om min deltagelse i dette forsøg*

Hvis der kommer nye væsentlige helbredsoplysninger frem om dig i dette forsøg vil du blive informeret. Såfremt du ikke ønsker at modtage nye helbredsoplysninger bedes du markere her:
☐ (sæt x)

Ønsker du at blive informeret om forsøgets resultater samt eventuelle konsekvenser for dig?
☐ *Ja (sæt x)* ☐ *Nej (sæt x)*

Underskrift til samtykke til deltagelse i forsøget (hvis relevant):

______________________	______________________	______________________
Patientens navn	Underskrift	Dato

Erklæring fra forsøgslægen, forsøgssygeplejerske eller anden person udpeget af forsøgslederen, som afgiver informationen:
Jeg bekræfter, at jeg har forklaret indholdet af denne patientinformation og informeret samtykke for den ovennævnte person. Jeg har spurgt, om der er nogle spørgsmål, og jeg har besvaret alle spørgsmål, der er fremført.

Læge/sygeplejerske navn	Underskrift	Dato

Appendix I – Guidelines for oral information

Retningslinjer for mundtlig deltagerinformation.
Informationen gives af en person, som har de sundhedsfaglige forudsætninger for at kunne informere om forskningsprojektet og som har direkte tilknytning til dette, hvilket i praksis betyder at informationen gives af læge eller forskningssygeplejerske på et af de hospitaler der deltager i undersøgelsen.

Forsøgspersonerne er identificeret ved:

- Et registertræk hvor man har fundet personer der opfylder in- og eksklusionskriterier
- Personer der løbende identificeres på de deltagende hospitaler (i forbindelse med indlæggelse eller ambulant kontrol) og som opfylder in- og eksklusionskriterier
- Personer som henvender sig til hospitalerne fordi de ønsker at deltage og som opfylder in- og eksklusionskriterier (disse vil potentielt kunne komme fra andre end de 3 deltagende regioner)

De potentielle forsøgspersoner vil få følgende initiale skriftlige information:

LOOP forsøget

Titel: Sammenhængen mellem blodprop i hjernen og atrieflimren påvist ved langvarig registrering af hjerterytmen med loop recorder.

Med dette brev vil vi spørge, om du vil deltage i en forskningsundersøgelse. Forsøget har til formål at afklare, om man kan forebygge blodprop i hjernen ved at overvåge hjerterytmen med et lille apparat (en såkaldt loop recorder), der placeres under huden på forsiden af brystkassen. Nogle af de personer der deltager vil få placeret det lille apparat, mens andre ikke vil få dette (dette afgøres ved en lodtrækning). I forskningsundersøgelsen deltager i alt 6.000 patienter og af disse vil hver fjerde (dvs. 1.500) efter lodtrækning få placeret det lille apparat.

Vi har fundet frem til dig ved at søge i registre efter personer med adresse i Region Hovedstaden, Region Sjælland eller Region Syddanmark, der er fyldt 70 år og desuden har en eller flere af sygdommene: Forhøjet blodtryk, sukkersyge, hjertesvigt eller en tidligere blodprop i hjernen (”slagtilfælde”). Du kan også være blevet fundet i forbindelse med en nylig hospitalskontakt, eller ved at du har kontaktet os, fordi du har hørt om forsøget. Nogen gange kan der være fejl i

registerdiagnoser, så hvis du fejlagtigt er blevet registreret med en af de nævnte diagnoser, beder vi dig orientere os herom.

Såfremt du allerede har fået påvist den hjerterytmeforstyrrelse, der kaldes atrieflimren kan du desværre ikke deltage. Du kan heller ikke deltage i forsøget, hvis du behandles med blodfortyndende medicin af typen Marevan, Pradaxa, Eliquis eller Xarelto

Hvis du er interesseret i at høre mere om forsøget kan du **kontakte projektsygeplejerske Sisse Lund (telefonnummer 35450686 eller e-mail: sisse.maj.lund.jensen@regionh.dk),** som vil sikre, at du kan deltage i forsøget.

Såfremt du ønsker at deltage, vil det være en god ide, hvis du kan have et familiemedlem eller en god ven med til samtalen på sygehuset.

Med venlig hilsen

Jesper Hastrup Svendsen
Professor, dr.med.

Appendix J – Endpoint Committee Charter

Clinical Endpoint Committee (CEC) Charter
The LOOP Study

Version 6.
Date: 20th October 2016
Revised on March 15, 2017

Members of the clinical endpoint committee (CEC):
Professor Lars Køber (chairman), Rigshospitalet, University of Copenhagen.
Professor Derk Krieger, University of Zürich.
The members of the CEC consist of experts in the field of cardiology or neurology with expertise in atrial fibrillation and stroke.
With respect to adjudication of atrial fibrillation episodes the CEC has given authority to members outside the committee to adjudicate atrial fibrillation episodes as described below.

1 **Purpose**
The committee will provide standardized adjudication of relevant protocol-defined events for the LOOP study. Events will include trial endpoints (excluding Quality of Life) and important specific adverse events. This charter describes the roles and responsibilities of the committee for adjudication of the primary endpoints. All members of the committee will adjudicate all endpoints that potentially could be a primary endpoint.

2 **Roles of CEC Members**
The primary roles of CEC members will be:
1. To review and provide input for this Charter;
2. To adjudicate all reported events as per this Charter;
3. To classify all endpoints into their specific type according to the set of definitions in this document.
Processes for adjudication and definitions for the events that will be adjudicated by the committee are described in this Charter. The committee will review and adjudicate all strokes and systemic embolisms and all deaths. The committee can include additional expertise as required for evaluation of events, and may obtain assistance in assessment of events.

The primary endpoint is:
- time to stroke or systemic arterial embolism

Secondary endpoints are:
- time to adjudicated ischemic stroke/transient ischemic attack/systemic arterial embolism
- time to adjudicated stroke, systemic arterial embolism, or cardiovascular death
- time to adjudicated cardiovascular death
- time to death by any cause

Other endpoints are:
- time to diagnosis of AF
- time to initiation of OAC
- time to adjudicated intracranial hemorrhage not classified as stroke
- time to adjudicated hemorrhagic stroke
- time to major bleeding as defined by the International Society on Thrombosis and Hemostasis

- complications related to loop recorder implantation
- change from baseline in quality of life

3. **Reporting events to the Committee**

Potential endpoints will be reported in the eCRF, and supporting documentation (e.g., admission summaries, progress records, diagnostic test reports, consultant evaluations, discharge summaries, death certificates, autopsy reports) will be forwarded by each center to the Study Office at Rigshospitalet. The study administrator will gather all relevant material in patient specific binders, which will be used by the CEC. The study administrator will interact with study sites to collect additional documentation needed to prepare a complete document package for committee adjudication.

4. **Documentation for CEC adjudication**

Supporting documentation required for adjudication of an event may include but is not limited to the following:

- Emergency Department records
- Pertinent laboratory reports
- Selected reports from any other test performed to evaluate the patient
- Hospital admission notes and pertinent hospital record notes surrounding the event (physician notes and nursing notes)
- Selected consultant reports and pathology reports
- Hospital or Emergency Department discharge summaries
- Death/autopsy summary
- Selected relevant hospital records
- Narrative summary of the event

The supporting documentation will have all patient identifiers (name, CPR number and information about randomization) removed or completely obscured prior to submission to the study office and supplied with a study number.

Whenever possible, suspected endpoints will be presented to the CEC only when a complete package of information has been obtained (e.g., all appropriate eCRF information and an adequate set of supporting documentation).

Adjudicators will review the documentation as hard copies.

5. **Evaluation of the event by the Committee**

All cases of potential stroke, TIA or systemic embolism (or death) will be reviewed independently by two CEC members. If the committee members agree on the adjudication, the adjudication is considered complete. If the committee members disagree on adjudication, the event will be reviewed during a CEC meeting. If an event cannot be classified due to a lack of supportive documentation, the CEC will ask for additional information from the study coordinator. After all possible information is gathered, the CEC will render a final adjudication on the event. In certain death cases, only limited medical information may be available. CEC members should use their best clinical judgment when

rendering a final decision based on available data and the event definitions within this Charter.

6. **Reporting of the Committee decision**
The completion of the adjudication eCRFs constitutes the committee decision. This decision will not be communicated to the sites before the end of the study.

7. **Definition of events for CEC**
Events requiring adjudication include:

- Stroke (or TIA)
- Systemic embolism
- Death

Source documentation will be reviewed for determination as to whether the event meets the classification criteria established in this charter. Decisions by the committee will be based on the documentation provided and good clinical judgment.
The CEC adjudication categories for study events are as follows. Further information for each is provided below.

ADJUDICATION OF STROKES
Brain imaging is required for all patients with symptoms of stroke or TIA to distinguish hemorrhagic from ischemic stroke.

1: Stroke is defined as the sudden onset of a focal neurologic deficit due to acute infarction of cerebrovascular cause lasting at least 24 hours or interrupted by death, and is categorized as follows:
-1a: Ischemic stroke, defined as stroke caused by lack of blood supply (e.g. thrombosis, embolism, systemic hypoperfusion or cerebral venous sinus thrombosis)
-1b: Hemorrhagic stroke, defined as a stroke caused by cerebral or subarachnoid hemorrhage.
-1c: Unspecified stroke, in case the patient did not undergo brain imaging or autopsy
Explanation: Above definitions have been used in the RE-LY, ARISTOTLE and ROCKET AF studies.

2: Transient ischemic attack (TIA) is defined as a transient episode of neurologic dysfunction caused by focal brain, spinal cord, or retinal ischemia, **without** acute infarction.
Explanation: TIA was originally defined clinically by the temporary nature (<24 hours) of the associated neurologic symptoms. However, the arbitrary nature of the 24-hour time limit and lack of specific pathophysiologic meaning hampered the clinical and research utility of the term "TIA." Recognition of these problems led to a change to a tissue-based definition of TIA. The change was driven by advances in neuroimaging that enabled very early identification of ischemic brain injury and endorsed by 2009 guidelines from the American Heart Association and American Stroke Association (AHA/ASA).

ADJUDICATION OF SYSTEMIC EMBOLISMS (SYSTEMIC EMBOLIC EVENTS; SEE)
SEE include arterial embolic events except those that reach the brain; SEE may reach organs such as kidney, spleen, heart, intestine, eyes and extremities.
Adjudication is based on clinical evaluation of patient data.

ADJUDICATION OF BLEEDING EPISODES
Major bleeding is defined according to the ISTH criteria as bleeding in a critical site (e.g. symptomatic intracranial bleeding (hemorrhagic stroke (see above) or subdural hemorrhage)), or clinically overt bleeding accompanied by a decrease in the hemoglobin level of at least 20 g per deciliter or transfusion of at least 2 units of packed red cells, or bleeding resulting in death. Nonmajor bleeding is defined as clinically overt bleeding that does not satisfy the criteria for major bleeding but leads to hospital admission, physician-guided medical or surgical treatment, or a change in antithrombotic therapy.

ADJUDICATION OF DEATHS
All deaths require adjudication by all committee members and classification as cardiovascular vs. non-cardiovascular and for cardiovascular deaths sudden vs. non-sudden. If the committee finds that a death is in fact caused by stroke, this will be registered as a primary endpoint in the eCRF.
Cardiovascular death includes

- Heart failure/cardiogenic shock (pump failure)
- Sudden cardiovascular death – witnessed
- Sudden cardiovascular death – unwitnessed
- Other cardiovascular causes (Acute coronary syndrome, cerebrovascular accident, Peripheral Vascular Disease, Systemic embolus, Pulmonary embolus)
- Cardiac or vascular procedure complication
- Other cardiovascular death, specify
- Presumed cardiovascular death (includes events classified as “unknown” cause of death)

Non-cardiovascular death includes

- Renal Failure
- Pulmonary (other than pneumonia)
- Pneumonia
- Sepsis
- Other infection
- Gastrointestinal (including GI hemorrhage)
- Seizure (not related to cerebrovascular accident, CVA)
- Other neurologic
- Hematologic
- Malignancy

- Non-cardiovascular procedure complication

Definitions used to classify cardiovascular deaths
Death due to Heart Failure/Cardiogenic Shock (Pump failure):
Death occurring in the context of clinically worsening symptoms and/or signs of heart failure without evidence of another cause of death. New or worsening signs and/or symptoms of congestive heart failure (CHF) may include any of the following:

- New or increasing symptoms and/or signs of heart failure (such as dyspnea, fatigue, edema) requiring the initiation of, or an increase in, treatment directed at heart failure or occurring in a patient already receiving maximal therapy for heart failure
- Heart failure symptoms or signs requiring continuous intravenous therapy or oxygen administration
- Confinement to bed predominantly due to heart failure symptoms
- Pulmonary edema sufficient to cause tachypnea and distress not occurring in the context of an acute myocardial infarction or as the consequence of a primary arrhythmic event
- Cardiogenic shock, manifest as clinical signs and symptoms of hypoperfusion assumed to be secondary to cardiac dysfunction, and not occurring in the context of an acute myocardial infarction or as the consequence of a primary arrhythmic event
- Patients who are hospitalized and are being actively treated for heart failure and who have a sudden death as the terminal event will be classified as having a heart failure related death.

Sudden Presumed Cardiovascular Death:
Death that occurs unexpectedly in a previously stable patient will be adjudicated as witnessed or unwitnessed sudden cardiac deaths:
Witnessed sudden cardiac death:

- Witnessed and instantaneous without new or worsening symptoms
- Witnessed within 60 minutes of the onset of new or worsening cardiac symptoms
- Witnessed and attributed to an identified arrhythmia (e.g., captured on an electrocardiographic (ECG) recording or witnessed on a monitor by either a medic or paramedic or found by interrogation of a device (ILR/ICD/CRT/pacemaker)
- Subjects unsuccessfully resuscitated from cardiac arrest or successfully resuscitated from cardiac arrest but who die without identification of a non-cardiac etiology
  Note that if a witnessed sudden cardiac death occurs as a complication of another primary cardiac process, eg, cardiogenic shock or acute myocardial infarction, the primary process will be adjudicated as the cause of death.

Unwitnessed Sudden Presumed Cardiac Death:

An unwitnessed death is one that occurs in a patient who, when last seen alive, within an observation period of 72 hours:

- Did not manifest another life-threatening non-cardiac disease (e.g., infectious, metabolic disorders);

and/or

- Did not reveal a cause other than cardiovascular (e.g., trauma) at the scene of death;

and/or

- Death was ruled cardiovascular in cause on an autopsy report or death certificate, and occurred in the absence of pre-existing circulatory failure or other modes of death.

If an unwitnessed sudden cardiac death occurs, information regarding the patient's clinical status within the week preceding death should be reviewed for potential insight into the primary cause of death.

Non-Sudden Death due to Other Cardiovascular Causes:
These are defined from medical records and include acute coronary syndrome, cerebrovascular accident (stroke), peripheral vascular disease, systemic embolus, pulmonary embolus, cardiac or vascular procedure complication, other specified cardiovascular death

Non-Sudden Presumed Cardiovascular Death:
All deaths not attributed to the categories of cardiovascular death and not attributed to a non-cardiovascular cause, are presumed cardiovascular deaths and as such are part of the cardiovascular mortality endpoint.

Definitions used to classify non-cardiovascular deaths
Non-cardiovascular death is defined as any death not covered by cardiac death or vascular death and includes the following:

- Renal Failure
- Pulmonary (other than pneumonia)
- Pneumonia
- Sepsis
- Other infection
- Gastrointestinal (including GI hemorrhage)
- Seizure (not related to CVA)
- Other neurologic
- Hematologic
- Malignancy
- Non-cardiovascular procedure complication
- Other (specify)

Non CV death will primarily be reported as yes or no. In cases where patients are admitted to hospital for a cardiovascular reason and dies with an infection the event will be classified as CV death.

ADJUDICATION OF ATRIAL FIBRILLATION EPISODES

Automatically transmitted data from the ILR´s are sent to the central database. Every day an experienced medical doctor reviews the recent transmissions in the database. This monitoring will continue until the end of battery life or until death occurs. Subjects will be contacted if transmissions are not received, and system malfunctions will be corrected. If an AF episode lasting minimum 6 minutes is detected, two senior cardiologists independently evaluate the transmission. In case of disagreement, a third senior cardiologist will independently evaluate the episode. If the two or two of three cardiologists confirm the diagnosis of AF, OAC will be initiated by the local center according to national guidelines, and this will be registered in the eCRF. Additional 12-lead ECG s will be obtained according to standard care regimens in both randomized arms and these investigations will be evaluated according to standard care.

Summary of changes to the protocol

Summary of changes to the protocol approved by the Ethics Committee

LOOP study protocol version 61 vs. version 63
Protocol number in Ethics Committee: H-4-2013-025

PROTOCOL VERSION	Approved by Study Steering Committe	Approved by Ethics Committe
Protocol vs. 61	December 5th, 2013	December 20th, 2013
Protocol vs. 63	August 16th, 2019	August 21st, 2019

SECTION 5: SUBJECT ENROLMENT

It is specified that data are stored in a web-based electronic eCRF protected by encryption (initially described as a physically main database).

SECTION 7: STUDY PROCEDURES

Section 7.2.1 Follow-up Phase main study:
More details about follow up is described.
Information about Echocardiography substudy is given.

SECTION 10: STATISTICAL CONSIDERATIONS

Section 10.2 Study Endpoints and Subsets:
Specification of Secondary and Other endpoints.

Section 10.3 Sample Size Considerations:
The necessary number of adjudicated primary events were recalculated to 279 events (instead of 230) due to a calculation error.

Section 10.6: Planned Methods of Analysis
The preliminary plan for data analysis is described in more details compared with previous protocol version.

Appendix F: Definition of the composite endpoint.
Clarification of definition of the primary endpoint.

Appendix H: Informed consent form
The informed consent form (in Danish) is inserted.

Appendix J: Clinical Endpoint (CEC) Charter version 6 of October 20th, 2016 and updated on March 15th, 2017 is included.

In addition, linguistic improvements and clarifications in the text were done.

Original statistical analysis plan (SAP)

ORIGINAL STATISTICAL ANALYSIS PLAN

This is a copy of the text from study protocol vs. 63 as details for the statistical Analysis Plan were not made until 2020.

10.6 Planned Methods of Analysis:

10.6.1 General Approach/Considerations:

Baseline characteristics, demographics, pre-existing conditions, previous illnesses, and possible prior treatments will be summarized for each treatment group. Subjects disposition and reason for discontinuation will be summarized for each group. The primary and secondary efficacy endpoints are timed to event variables and survival techniques will be utilized to perform statistical estimation and hypothesis testing. Concomitant medications will be summarized for each treatment group and compared between groups to access their potential impact on the primary endpoint.

10.6.2 Analysis of Key Study Endpoints:

10.6.2.1 Primary Endpoint:

The principle analysis for the primary endpoints will employ the intend-to-treat principle, and use a survival analysis. To account for the competing risk of death, the cumulative incidences of the primary endpoint will estimated, plotted and group-wise compared in a multi-state fashion. A co-variate adjusted analysis of the combined primary endpoint using a cause-specific Cox proportional regression model will be performed as a supportive analysis. The hazard ratios and the corresponding 95% confidence intervals will be estimated. Subjects completing the study and not reaching the composite endpoint will be censored.

10.6.2.2 Secondary Endpoints:

See section 10.2.2.

For the time-to-event variables, analysis similar to the one employed for the primary endpoint will be used. The censoring mechanism will also be similar to the one used for the analysis of the primary endpoint. The secondary endpoints will be tested at a significance level of 0.05. Within the first two secondary endpoints the type 1 error rate associated with a multiplicity of comparisons will be controlled according to the Hochberg adjustment. The Quality of Life endpoint will be tested at the 5% significance level. Quality of Life score will be analysed as repeated measures analysis of covariance mixed effects model using baseline score as a covariate.

10.6.2.3 Safety Endpoint:

AEs: Subjects incidence rates of all AEs will be tabulated by system class,
preferred term, severity, and relationship to investigational product. Tables and/or narratives of

“on-study” death, and serious and significant AEs, including those causing early withdrawal, will also be provided.
Complications from ILR implantation will be asked for and registered at all visits.

Final statistical analysis plan (SAP)

Atrial Fibrillation detected by continuous ECG monitoring using implantable loop recorder to prevent stroke in high-risk individuals

The LOOP study

Statistical analysis plan

Version number:	1.10 (final version)
Version date:	2020-Oct-01

Final version approved by the Steering Committee at meeting 2020-Oct-01

This document contains an **overview** of the methodology adherent to the LOOP study (protocol number H-4-2013-025, clinicaltrials.gov identifier NCT02036450), followed by the **statistical analysis plan** for the primary reporting of the study.

The document describes the analyses to be performed for the main outcome paper adhering to the randomized trial. The primary analysis of the study will be conducted when at least 279 primary outcomes have occurred.
The purpose of the document is to clarify the methodology for the analyses and to avoid misleading inference from any post-hoc approach. Therefore, the statistical analysis plan has been completed prior to the availability of outcome data.

STUDY OVERVIEW

The principal research question is the following: *Can screening for atrial fibrillation (AF) with implantable loop recorder and initiation of oral anticoagulation (OAC) if AF is detected reduce the risk of stroke and systemic arterial embolism in persons with risk factors for stroke.* The study is a multi-center randomized controlled trial conducted at four centers located in 3 of Denmark's 5 administrative regions. Study oversight is performed by a Steering Committee consisting of senior cardiologists from all study centers, senior neurologists, health economists, and computer scientists.

Inclusion Criteria:

- Age 70-90 years at the initial screening visit, and
- Previously diagnosed with ≥1 of the following:
    - Diabetes mellitus (type 1 or type 2, with or without medical therapy)
    - Hypertension (with or without medical therapy)
    - Heart failure
    - Stroke (previous transient ischemic attack (TIA) is not an inclusion criterion)

Exclusion Criteria:

- History of atrial fibrillation or flutter irrespective of type
- Cardiac pacemaker or defibrillator (with or without re-synchronization therapy)
- Contraindication to oral anticoagulation therapy
- Anticoagulation therapy; vitamin K antagonists, direct oral anticoagulants, or (low-molecular) heparins. Therapy with platelet inhibitors such as acetyl-salicylic acid, clopidogrel, persantine is not considered an exclusion criterion
- Renal failure treated with permanent dialysis
- Uncorrected congenital heart disease, or severe valvular stenosis, obstructive cardiomyopathy, active myocarditis, or constrictive pericarditis.
- On a waiting list for major surgery (cardiac, thoracic or abdominal)
- Cardiac or thoracic surgery has been performed within 3 months from inclusion
- Any major organ transplant (e.g. lung, liver, heart, or kidney)
- Cytotoxic or cytostatic chemotherapy and/or radiation therapy for treatment of a malignancy within 6 months before randomization or clinical evidence of current malignancy with the following exceptions: Basal or squamous cell carcinoma of the skin, cervical intraepithelial

neoplasia, prostate cancer (if stable, localized disease with a life expectancy of > 2.5 years in the opinion of the investigator)
- Life-expectancy shorter than 6 months
- Known to be human immunodeficiency virus (HIV) positive with an expected survival of less than 5 years due to HIV infection
- Recent (within 3 months) history of alcohol or drug abuse based on self-reporting
- Any condition (e.g. psychiatric illness, dementia) or situation, that in the investigators opinion could put the subject at significant risk, confound the study results, or interfere significantly with the subject participation in the study
- Unwillingness to participate or does not understand Danish language

Enrollment:
Potentially eligible study participants from the general population are identified by random registry draw and receive a letter invitation to an initial screening visit at the relevant study center. The screening visit is performed by study nurses under supervision by a medical doctor and investigator in the trial. At the screening visit, study eligibility will be confirmed and a standard 12-lead ECG will be taken to rule out prevalent atrial fibrillation or flutter. If eligible, informed consent will be obtained and baseline data will be collected. A total of 6000 participants will be enrolled.

Consent
Written and signed informed consent is obtained from all participants prior to randomization.

Randomization
Included participants will be randomized in a ratio 3:1 to control (n=4500) versus implantable loop recorder (ILR) (n=1500). There is no blinding, but data on the primary outcome is only viewed by the Study Monitoring research nurses.

Interventions and follow-up procedures
ILR group:
The intervention in the ILR group is implantation of a Reveal LINQ (Medtronic) device with continuous monitoring and daily automated remote transmissions. New arrhythmia episodes are reviewed daily by an experienced medical doctor. If AF lasting ≥6 minutes is detected and confirmed by at least two senior cardiologists, the patient will be recommended initiation of OAC treatment by the study center. Decision about specific type of OAC, and possible further clinical

work-up or treatment, is left to the treating physician and the patient. The remote monitoring continues until end of service of the device, patient withdrawal or other end-of-study.
Study visits to collect data and outcomes are scheduled annually until the 4th visit, and furthermore, outcomes are collected via lookup in medical records and registries on at least an annual basis until the finalization of the trial. The study visits are performed by study nurses under supervision by a medical doctor and investigator in the trial.

Control group:

The control group is followed according to standard of care, i.e. by their general practitioner. Study visits to collect data and outcomes are scheduled at inclusion and after 3 years. Furthermore, the participants are contacted by telephone after 1 and 2 years of follow-up, and outcomes are collected via lookup in medical records and registries on at least an annual basis until the finalization of the trial. The study visits are performed by study nurses under supervision by a medical doctor and investigator in the trial.

Adjudication of outcomes:

All deaths and all events that could possibly pose a primary outcome will be adjudicated by a clinical Endpoint Committee. The Endpoint Committee consists of specialist with expertise in both cardiology and neurology.

Study variables

At the initial screening visit, screen failures will be registered along with reason for ineligibility.

For randomized participants, the following baseline variables will be collected:

- Verification of informed consent
- Verification of study eligibility
- Age
- Sex
- Medical history:
    - Hypertension, Diabetes mellitus, Heart failure, Stroke, Arterial systemic embolism, Transient ischemic attack, Acute myocardial infarction, Percutaneous coronary intervention, Coronary bypass grafting, Valvular heart disease, Surgically treated peripheral artery disease, Percutaneously treated peripheral artery disease, Peripheral artery disease without intervention, Syncope, Chronic obstructive pulmonary disease, Hyperthyroidism
- Prescribed drugs, dosages and duration of treatment
- Smoking status and smoking pack years
- Weekly alcohol consumption

- Highest education achieved
- Blood biochemistry
    - Creatinine, High-sensitivity C-reactive protein, N-terminal pro–B-type natriuretic peptide (NT-proBNP), High-sensitivity Cardiac Troponin (type I or T)
- Physical examination
    - Height, Body weight, Resting blood pressure, Resting heart rate
- New York Heart Association functional classification
- Electronically stored 12-lead ECG
- Cognitive function assessment (MoCA)
- Quality of Life assessment (EQ-5D-5L)
- Symptom assessment (SF36 and symptom checklist)
- Treatment arm (randomly allocated to control or ILR)

Some participants will undergo further baseline evaluation which will not be part of the primary reporting of the study.

<u>During follow-up, the following variables will be collected:</u>

- Physical examination
    - Height, Body weight, Resting blood pressure, Resting heart rate
- New York Heart Association functional classification
- Electronically stored 12-lead ECG
- Cognitive function assessment (MoCA)
- Quality of Life assessment (EQ-5D-5L)
- Symptom assessment (SF36 and symptom checklist)
- ILR implantation including complications
- Changes in medication
- Outcomes (see below)

Outcome measures

<u>Primary outcome measures:</u>

1. Time to adjudicated stroke or systemic arterial embolism

<u>Secondary outcome measures:</u>

1. Time to adjudicated ischemic stroke/transient ischemic attack/systemic arterial embolism
2. Time to adjudicated stroke, systemic arterial embolism, or cardiovascular death
3. Time to adjudicated cardiovascular death
4. Time to death by any cause

<u>Other outcome measures:</u>

- Time to diagnosis of AF
- Time to initiation of OAC
- Time to adjudicated intracranial hemorrhage not classified as stroke
- Time to adjudicated hemorrhagic stroke
- Time to major bleeding as defined by the International Society on Thrombosis and Hemostasis
- Complications related to loop recorder implantation
- Change from baseline in quality of life

Sample size calculation

The study is event driven. Expected event rates were projected from the literature. In the control group, 3% of the participants are expected to have documented AF and initiate OAC. In the ILR group, 30% of the participants are expected to have documented AF qualifying for OAC during at least 3 years of continuous monitoring. The study population is expected to have a stroke rate of 0.7%/year in persons without AF and 2%/year in persons with AF not treated with OAC. The annual rate of the primary outcome in the control group is expected to be 1%.

To obtain statistical power to analyze an overall reduction in the primary outcome with a hazard ratio of 0.65, 279 events will be needed, and follow-up duration will be adjusted to accommodate this.

Study end

The study continues until at least 279 primary outcomes have occurred. The Steering Committee decides on a final date of study closure, after which all participants who are still followed for the primary outcome will undergo a final assessment of study outcomes, and this date be used as censoring time for the main analysis.

STATISTICAL ANALYSIS PLAN FOR MAIN OUTCOME PAPER

Statistical analyses will be performed using SAS and the R software. A two-sided P-value <0.05 will be considered statistically significant.

CONSORT diagram

A CONSORT diagram will be used to describe flow from the initial screening visit to randomization to end-of-study for all participants. Any participants not included in the analysis will be explained at the necessary number of levels. The diagram will present the number of participants assigned to ILR not receiving ILR or having their ILR turned off/removed prematurely, and participants receiving an ILR when randomized to control.

Baseline descriptions

Baseline summary statistics will be grouped by randomization arm and presented in a tabularized manner. Continuous variables will be presented as mean ± standard deviation for normally distributed variables compared by t-tests, and median (interquartile range) for nonnormally distributed variables compared by Wilcoxon rank-sum tests, while categorical variables will be presented as frequency and percentage compared by chi-squared tests. The number of missing observations will also be reported.

Primary outcome analysis

The primary outcome analysis will be an analysis of time to first stroke or systemic arterial embolism comparing the two randomization arms and adhering to the intention-to-treat principle. Participants enter the analysis on the day of randomization and are followed until the outcome, death, or the defined time of trial closure as indicated by the Steering Committee. Any participants lost to follow-up (not expected) will be censored at the last time known to be alive.
The cumulative incidence will be calculated, plotted, and groupwise compared according to the Aalen-Johansen method to account for the competing risk of death. Cumulative incidence curves will be drawn along with at-risk tables. No. of events (% of all) and HR (95% CI) for difference between the two arms will be reported.

Multivariable analysis of the primary outcome

Cause-specific multivariable Cox proportional hazards regression models (or Poisson regression if assumptions are violated) will be examined as a supplement to the primary analysis. The first model also includes age, sex and center. The second model will further include all baseline

variables that in descriptive statistics display statistically significant differences between the two arms. A final model will include all variables reported in the descriptive statistics. For all models, Schoenfeld and Martingale residuals will be assessed, and the models will be fitted to comply with the assumptions for valid Cox regression. The proportional hazard assumption will be tested using cumulative residuals, interaction by examining importance of interaction variables and linearity by examining importance of also including quartiles.

Interaction and subgroup analyses of the primary outcome

Interaction between effect of randomization arm and a limited number of prespecified baseline variables will be assessed in cause-specific Cox proportional hazards models (or Poisson regression if assumptions are violated). For illustration, a forest plot will be created showing the results of interaction analyses. For the forest plot, continuous variables will be dichotomized at the median, except for age which will be dichotomized at 75 years, and results for the subgroups will be shown. In case of a significant interaction term for other variables than age, the variable will be divided into tertiles to demonstrate any trend. The p-values shown will be those for the interaction between the dichotomized/trichotomized variables and treatment effect. The predefined subgroups to be compared are: age, sex, smoking status, hypertension, diabetes mellitus, heart failure, previous stroke, previous stroke/transient ischemic attack/systemic arterial embolism, CHA_2DS_2-VASc score, platelet inhibitors, beta blockers, renin angiotensin blockers, estimated glomerular filtration rate, high-sensitivity C-reactive protein, body mass index, resting heart rate, resting systolic blood pressure, resting diastolic blood pressure.

Sensitivity analyses of the primary outcome

A sensitivity analysis will include only participants who received the assigned intervention at randomization (ILR or control). A further sensitivity analysis will censor participants crossing over defined as premature termination of remote monitoring in participants assigned to ILR and commencement of ILR monitoring in participants assigned to control, respectively.

Analysis of secondary outcomes / adverse events

The predefined secondary outcomes to be reported in the main outcome paper are listed above. These will be analyzed using the same methodology as for the primary outcome. The exploratory analyses will be performed hierarchically: 1) combined endpoint of ischemic stroke/transient ischemic attack/systemic arterial embolism, 2) combined endpoint of adjudicated stroke, systemic arterial embolism, or cardiovascular death, 3) cardiovascular death, 4) all-cause mortality, and will

be reported as no. of events (% of all) and HR (95% CI) for difference between randomization arms in a tabularized manner.

Analysis of other outcomes / adverse events

The predefined other outcomes are listed above. These will be analyzed as for the primary outcome, but without sensitivity analyses and will be reported in a tabularized manner. Time to event outcomes will be reported as no. of events (% of all) and HR (95% CI) for difference across randomization arms. Other endpoints will be reported as no. (%), median (quartiles 1;3), or mean (standard deviation) for each randomization arm as appropriate.

Handling of missing data

Any missing data will be reported. Multiple imputation will be used to increase precision of the estimates and to accommodate potential biases from a complete case analysis. Missingness occurring at a rate above 5% and below 40% in any variable will be imputed in the outcome analyses and sensitivity analyses with/without imputation will be conducted to assess the impact of imputation.

Overview of Figures and Tables

The following Figures and Tables will be included in the main outcome paper. The methodology is described above.

- Figure 1: CONSORT flow diagram
- Figure 2a-d: Cumulative incidence curves for the primary outcome and selected secondary outcomes grouped by randomization arm
    - a) Adjudicated stroke or systemic arterial embolism (primary outcome),
    - b) Adjudicated ischemic stroke/transient ischemic attack/systemic arterial embolism,
    - c) Adjudicated stroke, systemic arterial embolism, or cardiovascular death
    - d) All-cause mortality
- Figure 3: Forrest plot with HR for the primary outcome across predefined subgroups
- Table 1: Baseline variables grouped by ILR vs control with test for statistical difference
- Table 2: Additional outcomes in this document (Primary, Secondary, and Other) grouped by randomization arm reported as no. of events (% of all) and HR (95% CI)

Additional Figures and Tables will be included as Supplementary Material.

Summary of changes to the statistical analysis plan (SAP)

STATISTICAL ANALYSIS PLAN (SAP)

Date	Change	Explanation
October 1st, 2020	Final version, vs. 1.1<br>Not relevant	Not relevant

The statistical analysis plan was only briefly described in protocol vs. 63 (protocol version approved by the Steering Committee on August 16th, 2019 and approved by Ethics Committee on August 21st, 2019). The final statistical analysis plan was written in 2020 and approved by the Steering Committee October 1st, 2020.
