# Supplementary material for: Severity and Etiology of Incident Stroke in Patients Screened for Atrial Fibrillation vs Usual Care and the Impact of Prior Stroke: A Post Hoc Analysis of the LOOP Randomized Clinical Trial
Source: JAMA Neurol. 2022 Aug 29;79(10):997–1004. doi: 10.1001/jamaneurol.2022.3031 (PMC9425290; doi:10.1001/jamaneurol.2022.3031)
Supplement: Supplement 2. — eFigure 1. Trial Flow Chart eFigure 2. Cumulative Incidence of Recurrent Stroke on Patients With Incident Stroke eFigure 3. Stroke Hospital Stay Duration and Discharge Outcome Grouped by Randomization Group eFigure 4. Cumulative Incidence of Mild Stroke eFigure 5. Cumulative Incidence of Ischemic Stroke eFigure 6. Cumulative Incidence of Cardioembolic Stroke or ESUS eFigure 7. Cumulative Incidence of Severe Cardioembolic Stroke or ESUS eFigure 8. Cumulative Incidence of Mild Stroke by Stroke History eFigure 9. Cumulative Incidence of Ischemic Stroke by Stroke History eFigure 10. Cumulative Incidence of Cardioembolic Stroke or ESUS by Stroke History eFigure 11. Cumulative Incidence of Severe Cardioembolic Stroke or ESUS by Stroke History eFigure 12. Stroke Severity Grouped by AF Diagnosis and Randomization Group [file jamaneurol-e223031-s002.pdf]

Supplementary Online Content

Diederichsen SZ, Frederiksen KS, Xing LY, et al. Severity and etiology of incident stroke in patients screened for atrial fibrillation vs usual care and the impact of prior stroke: a post hoc analysis of the LOOP randomized clinical trial. *JAMA Neurol*. Published online August 29, 2022. doi:10.1001/jamaneurol.2022.3031

eFigure 1. Trial Flow Chart

eFigure 2. Cumulative Incidence of Recurrent Stroke on Patients With Incident Stroke

eFigure 3. Stroke Hospital Stay Duration and Discharge Outcome Grouped by Randomization Group

eFigure 4. Cumulative Incidence of Mild Stroke

eFigure 5. Cumulative Incidence of Ischemic Stroke

eFigure 6. Cumulative Incidence of Cardioembolic Stroke or ESUS

eFigure 7. Cumulative Incidence of Severe Cardioembolic Stroke or ESUS

eFigure 8. Cumulative Incidence of Mild Stroke by Stroke History

eFigure 9. Cumulative Incidence of Ischemic Stroke by Stroke History

eFigure 10. Cumulative Incidence of Cardioembolic Stroke or ESUS by Stroke History

eFigure 11. Cumulative Incidence of Severe Cardioembolic Stroke or ESUS by Stroke History

eFigure 12. Stroke Severity Grouped by AF Diagnosis and Randomization Group

This supplementary material has been provided by the authors to give readers additional information about their work.

© 2022 Diederichsen SZ et al. *JAMA Neurology.*

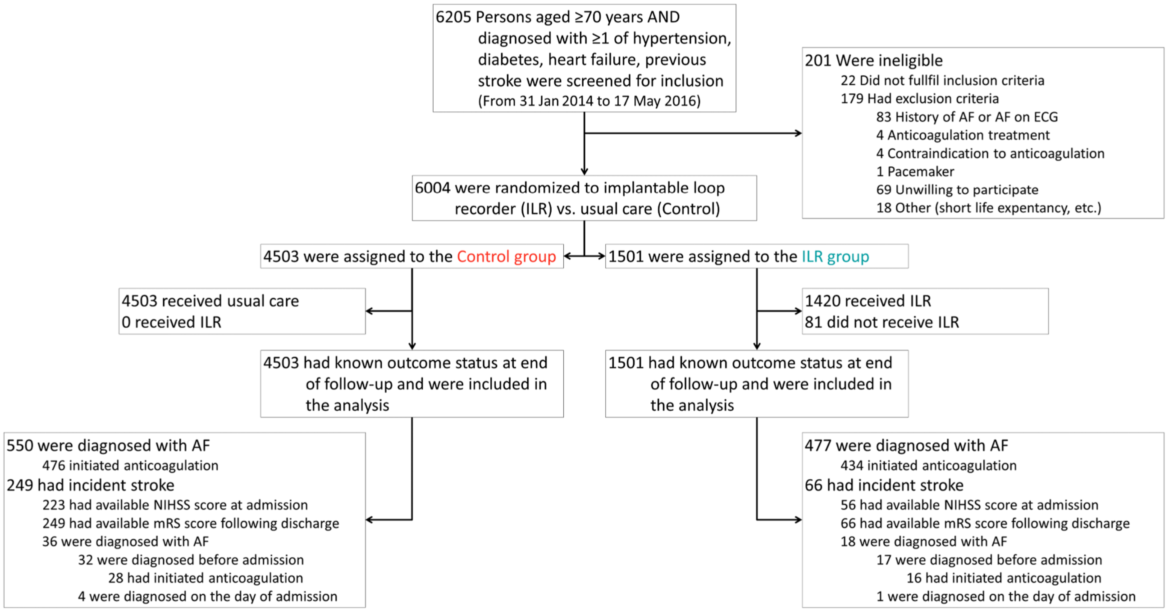

eFigure 1. Trial Flow Chart

The figure shows the flow of and diagnoses of included participants. AF, atrial fibrillation; ECG, electrocardiogram; ILR, implantable loop recorder; mRS, modified Rankin scale; NIHSS, National Institutes of Health Stroke Scale.

© 2022 Diederichsen SZ et al. *JAMA Neurology.*

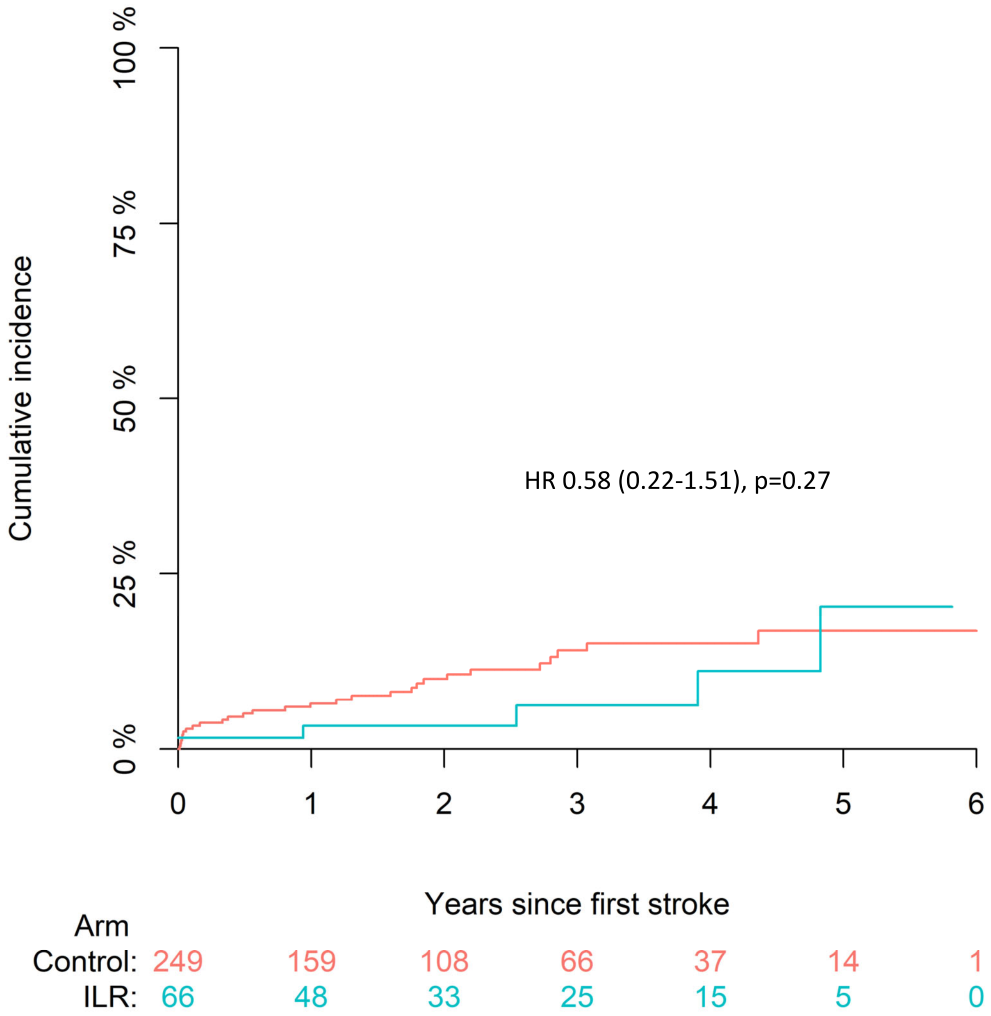

eFigure 2. Cumulative Incidence of Recurrent Stroke on Patients With Incident Stroke

The figure shows the cumulative incidence curves for recurrent stroke comparing the Control and ILR group. HR, hazard ratio; ILR, implantable loop recorder.

© 2022 Diederichsen SZ et al. *JAMA Neurology.*

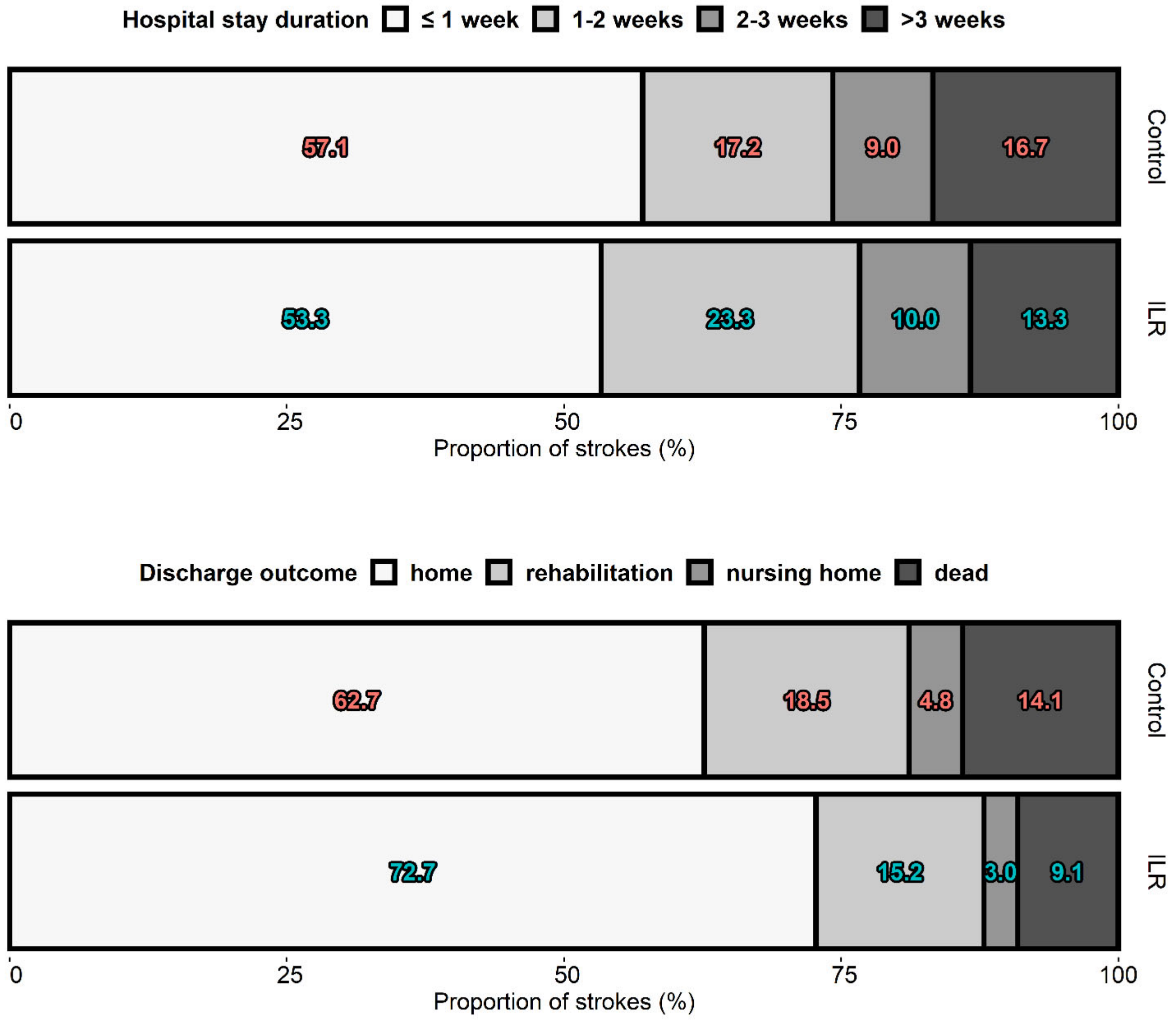

eFigure 3. Stroke Hospital Stay Duration and Discharge Outcome Grouped by Randomization Group

The figure shows the distribution of hospital stay duration (**upper panel**) and discharge outcome (**lower panel**) across stroke admissions in the Control and ILR group, respectively. A total of 18 stroke admissions (5.7%) lasted longer than 40 days. Wilcoxon rank sum test revealed no difference in hospital stay duration (p=0.86), and chi-squared tests revealed no significant difference in discharge strategy (p=0.47) or in discharge to home or not (p=0.12). ILR, implantable loop recorder.

© 2022 Diederichsen SZ et al. *JAMA Neurology.*

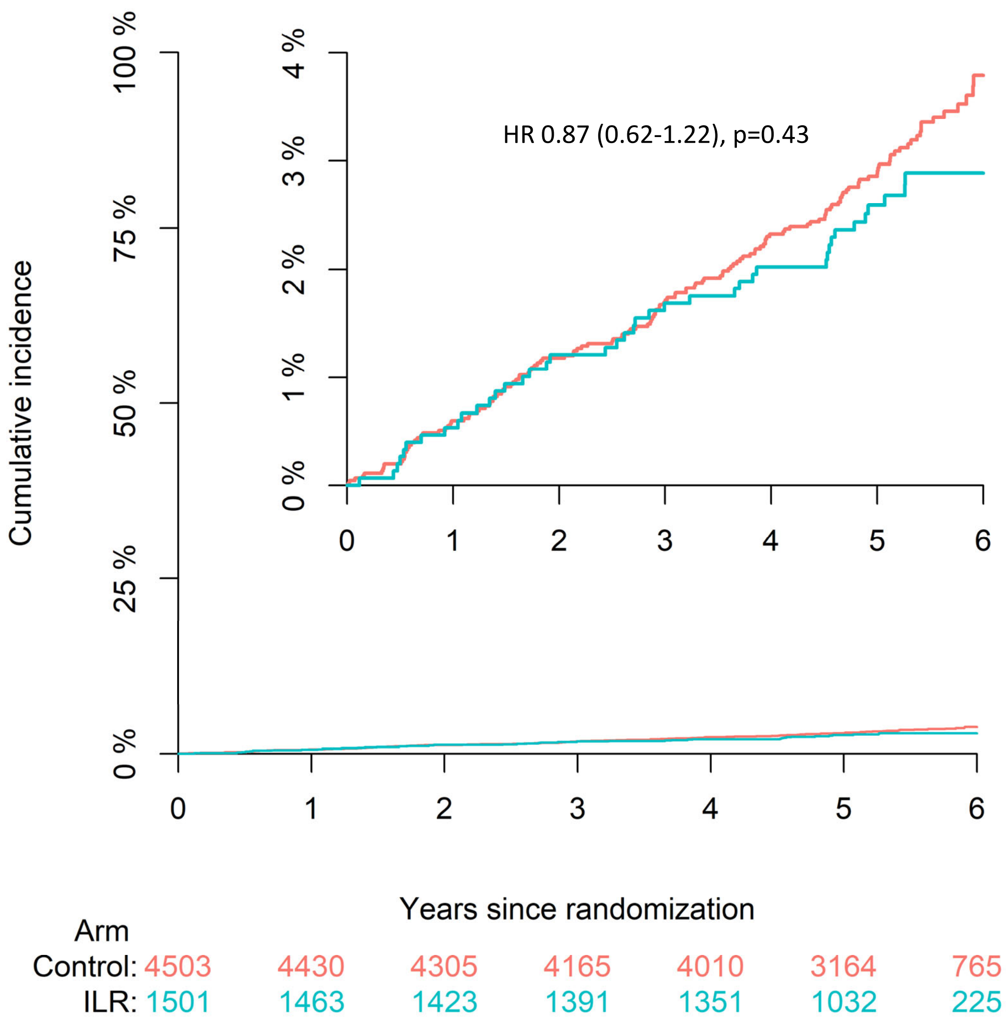

eFigure 4. Cumulative Incidence of Mild Stroke

The figure shows the cumulative incidence curves for mild (mRS<3) stroke comparing the Control and ILR group. HR, hazard ratio; ILR, implantable loop recorder; mRS, modified Rankin Scale.

© 2022 Diederichsen SZ et al. *JAMA Neurology.*

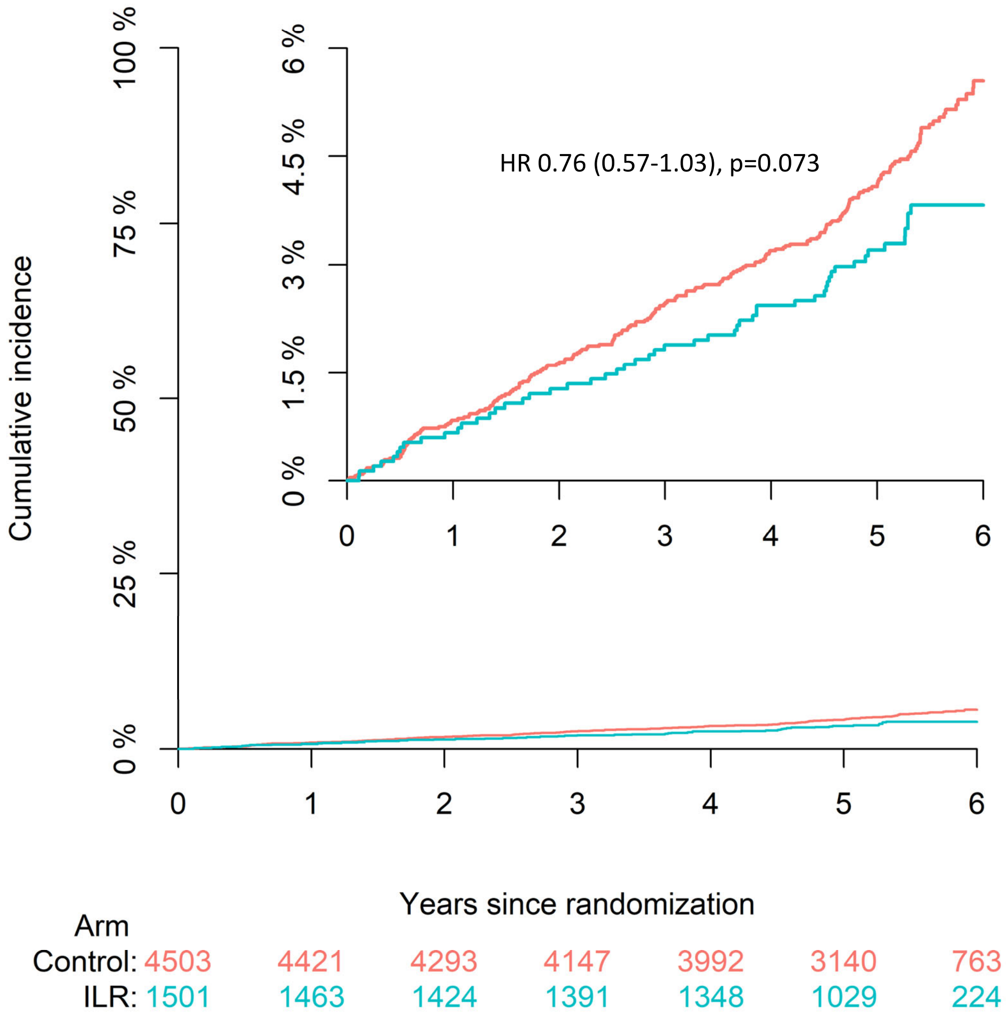

eFigure 5. Cumulative Incidence of Ischemic Stroke

The figure shows the cumulative incidence curves for ischemic stroke comparing the Control and ILR group. HR, hazard ratio; ILR, implantable loop recorder.

© 2022 Diederichsen SZ et al. *JAMA Neurology.*

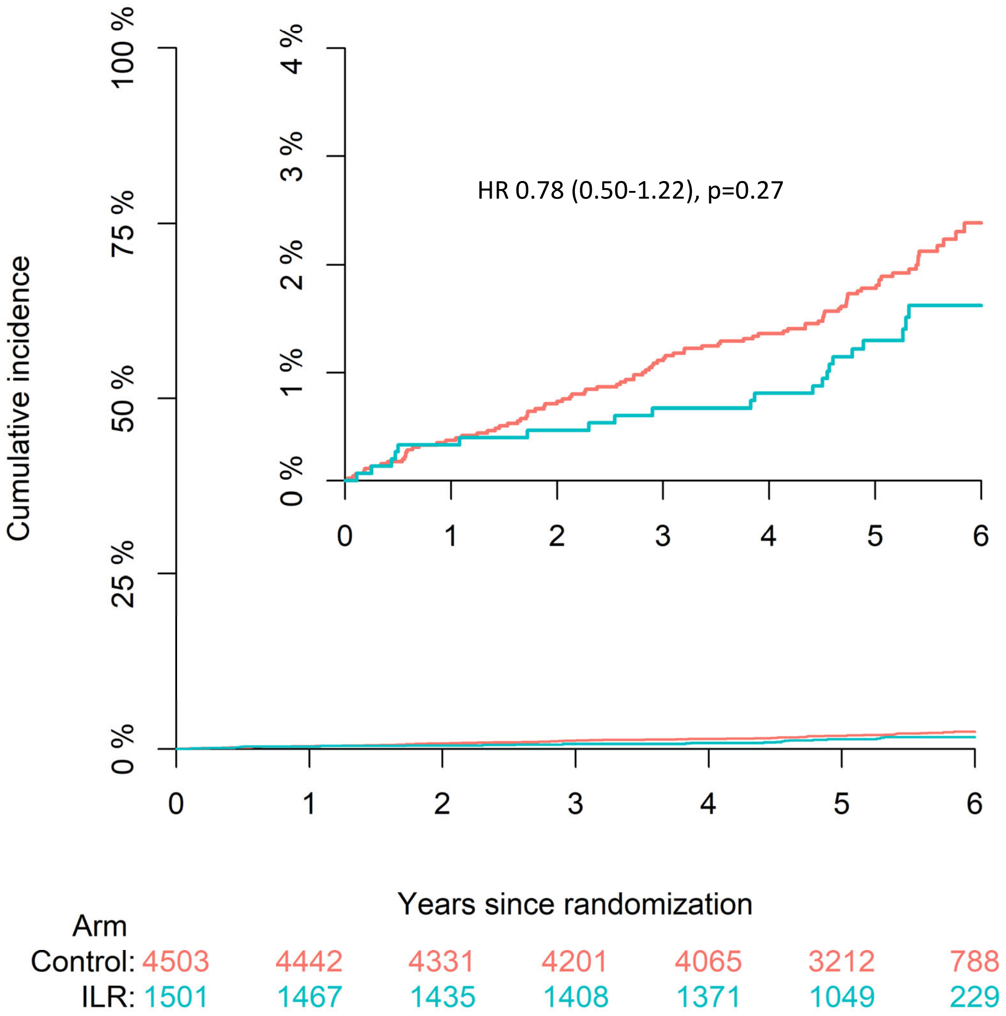

eFigure 6. Cumulative Incidence of Cardioembolic Stroke or ESUS

The figure shows the cumulative incidence curves for cardioembolic stroke or ESUS comparing the Control and ILR group. ESUS, embolic stroke of undetermined source HR, hazard ratio; ILR, implantable loop recorder.

© 2022 Diederichsen SZ et al. *JAMA Neurology.*

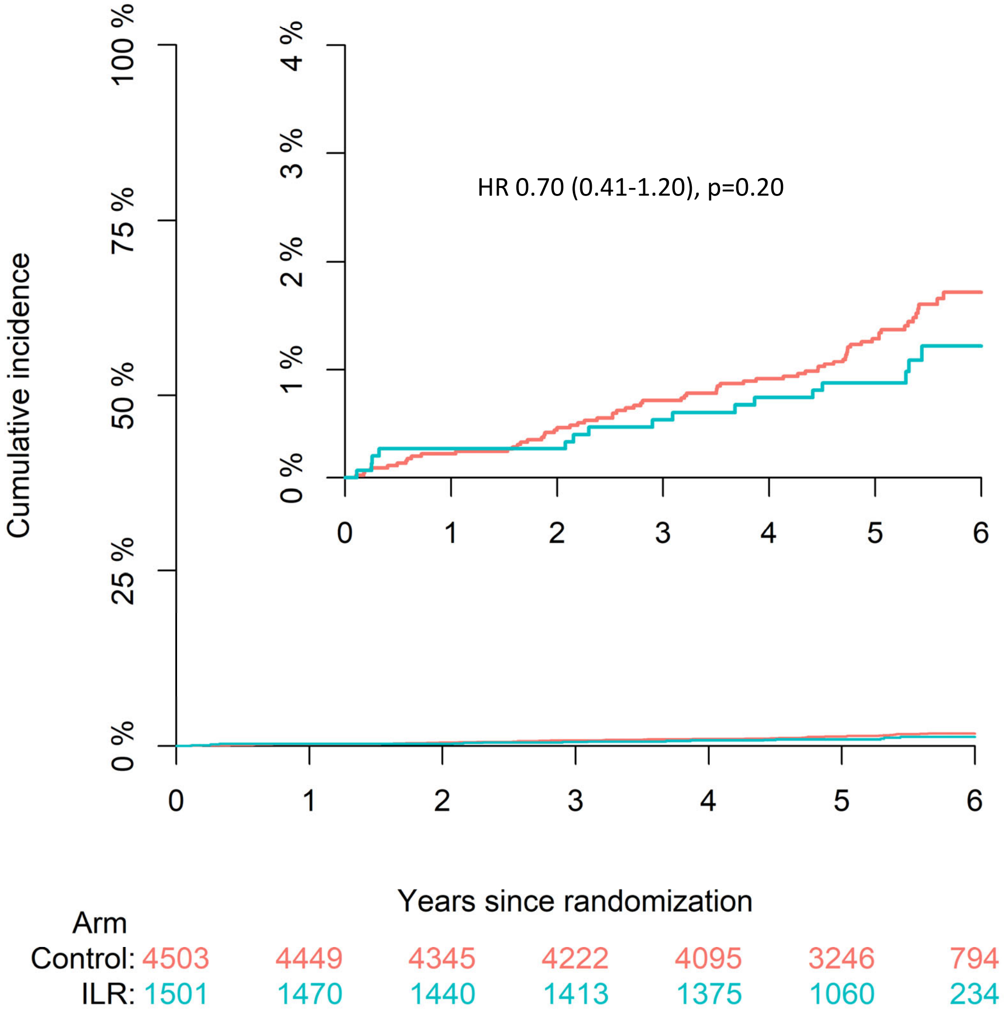

eFigure 7. Cumulative Incidence of Severe Cardioembolic Stroke or ESUS

The figure shows the cumulative incidence curves for disabling or lethal (mRS≥3) cardioembolic stroke or embolic stroke of undetermined source comparing the Control and ILR group. HR, hazard ratio; ILR, implantable loop recorder; mRS, modified Rankin Scale.

© 2022 Diederichsen SZ et al. *JAMA Neurology.*

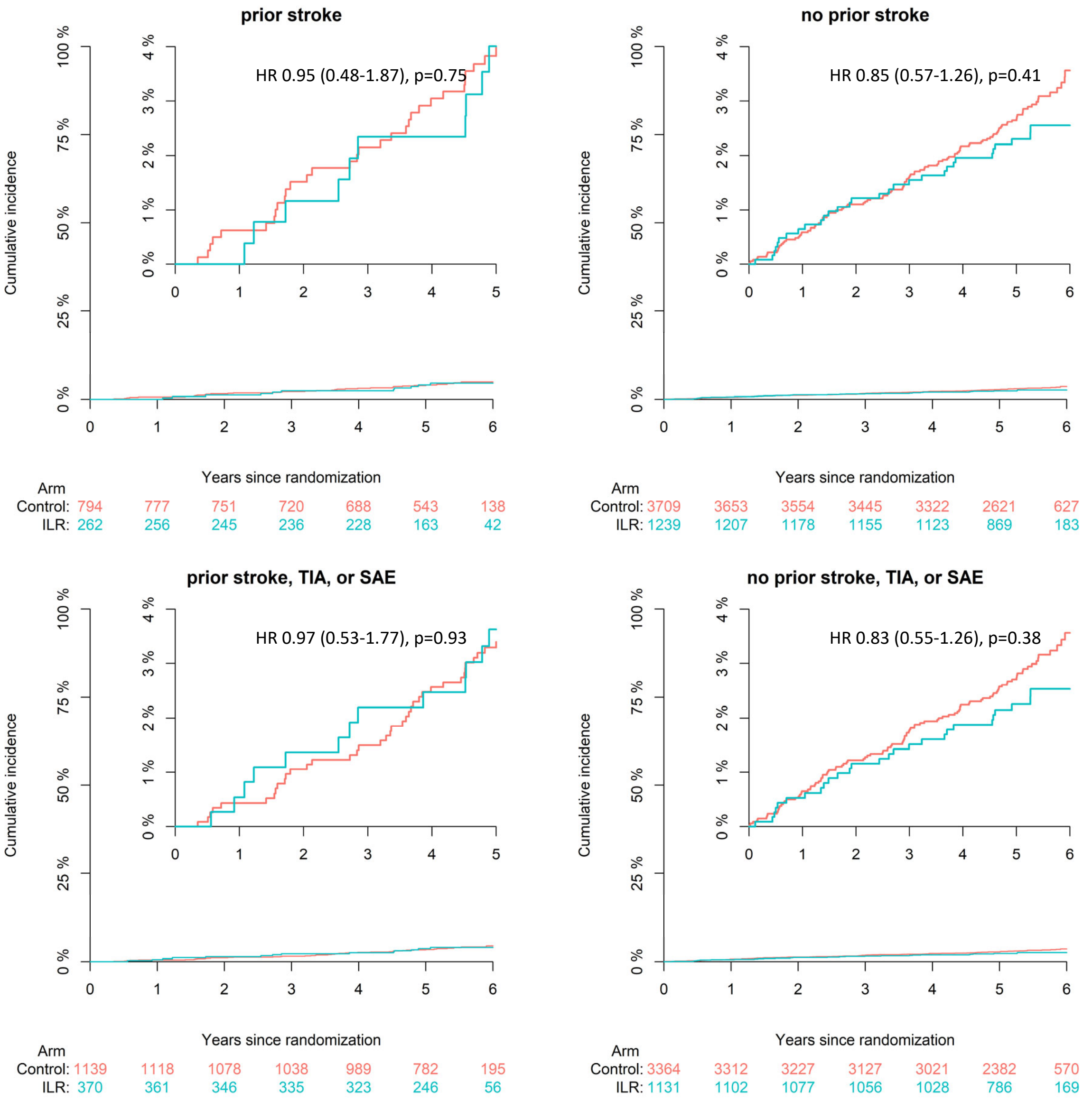

eFigure 8. Cumulative Incidence of Mild Stroke by Stroke History

The figure shows the cumulative incidence curves for mild (mRS<3) stroke comparing the Control and ILR group, stratified by prior stroke (**upper panels**) and prior stroke, TIA, or SAE (**lower panels**), respectively. HR, hazard ratio; ILR, implantable loop recorder; mRS, modified Rankin Scale; SAE, systemic arterial embolism; TIA, transient ischemic attack.

© 2022 Diederichsen SZ et al. *JAMA Neurology.*

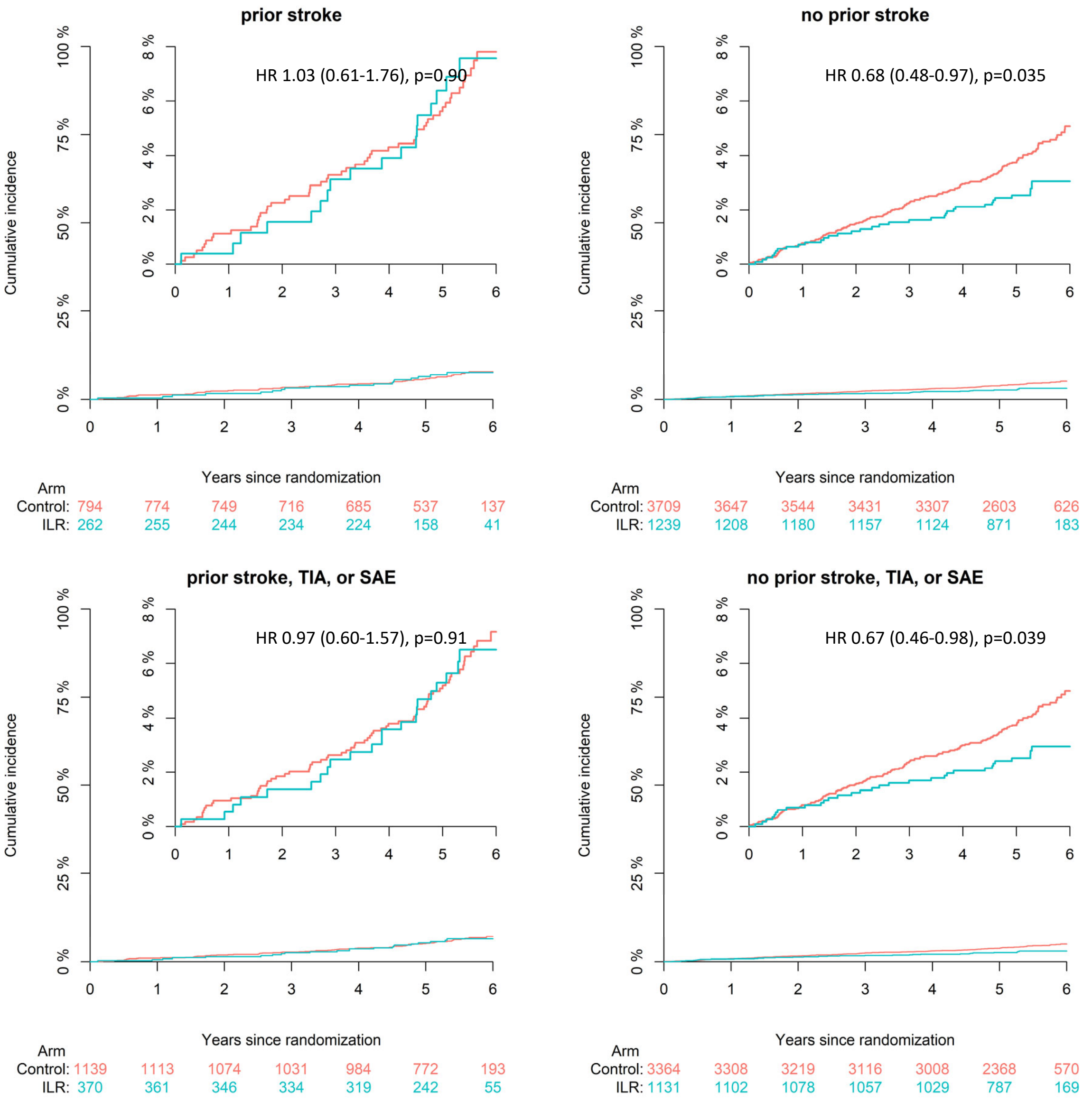

eFigure 9. Cumulative Incidence of Ischemic Stroke by Stroke History

The figure shows the cumulative incidence curves for ischemic stroke comparing the Control and ILR group, stratified by prior stroke (**upper panels**) and prior stroke, TIA, or SAE (**lower panels**), respectively. HR, hazard ratio; ILR, implantable loop recorder; SAE, systemic arterial embolism; TIA, transient ischemic attack.

© 2022 Diederichsen SZ et al. *JAMA Neurology.*

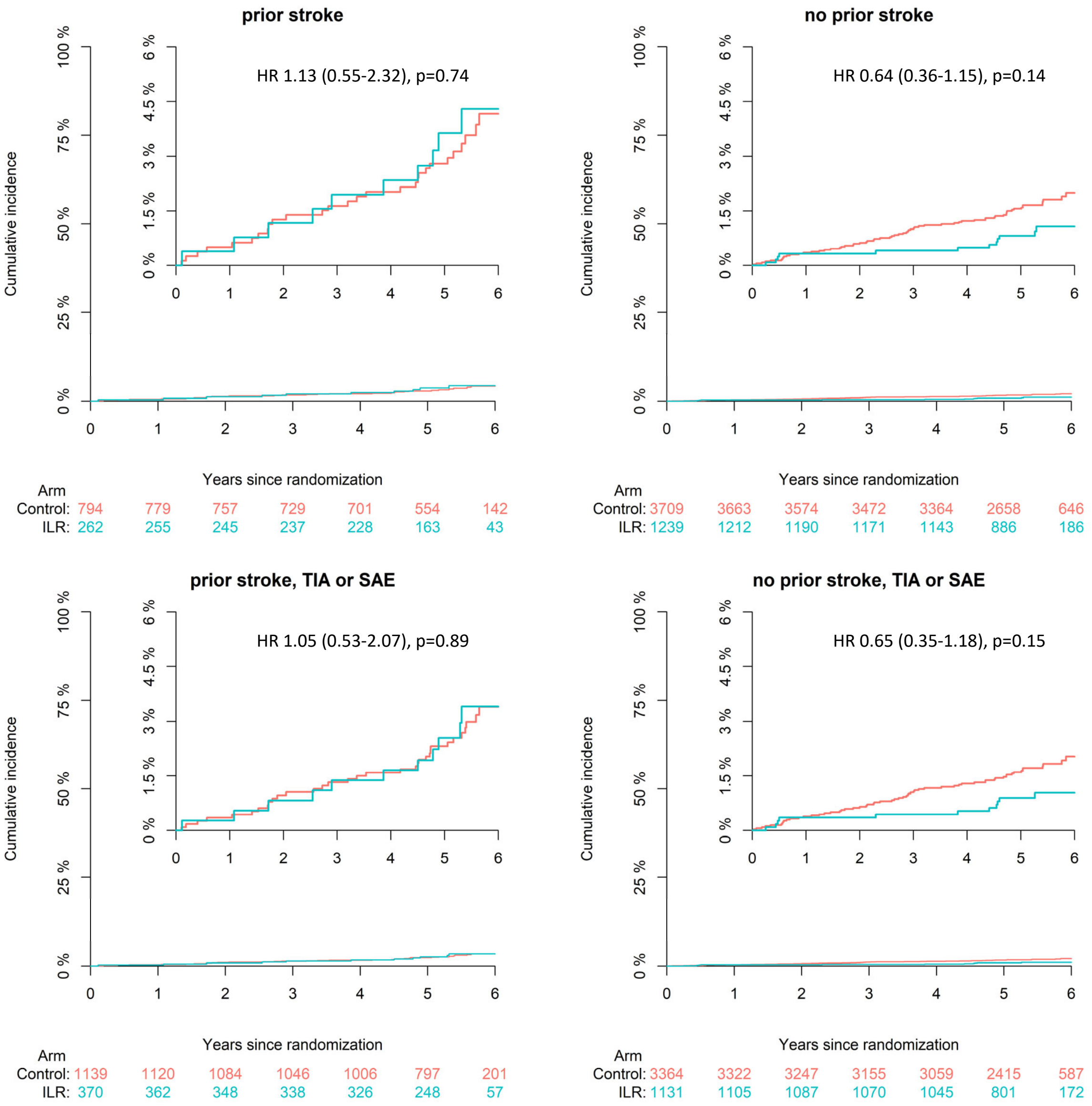

eFigure 10. Cumulative Incidence of Cardioembolic Stroke or ESUS by Stroke History

The figure shows the cumulative incidence curves for cardioembolic stroke or ESUS comparing the Control and ILR group, stratified by prior stroke (**upper panels**) and prior stroke, TIA, or SAE (**lower panels**), respectively. ESUS, embolic stroke of undetermined source; HR, hazard ratio; ILR, implantable loop recorder; SAE, systemic arterial embolism; TIA, transient ischemic attack.

© 2022 Diederichsen SZ et al. *JAMA Neurology.*

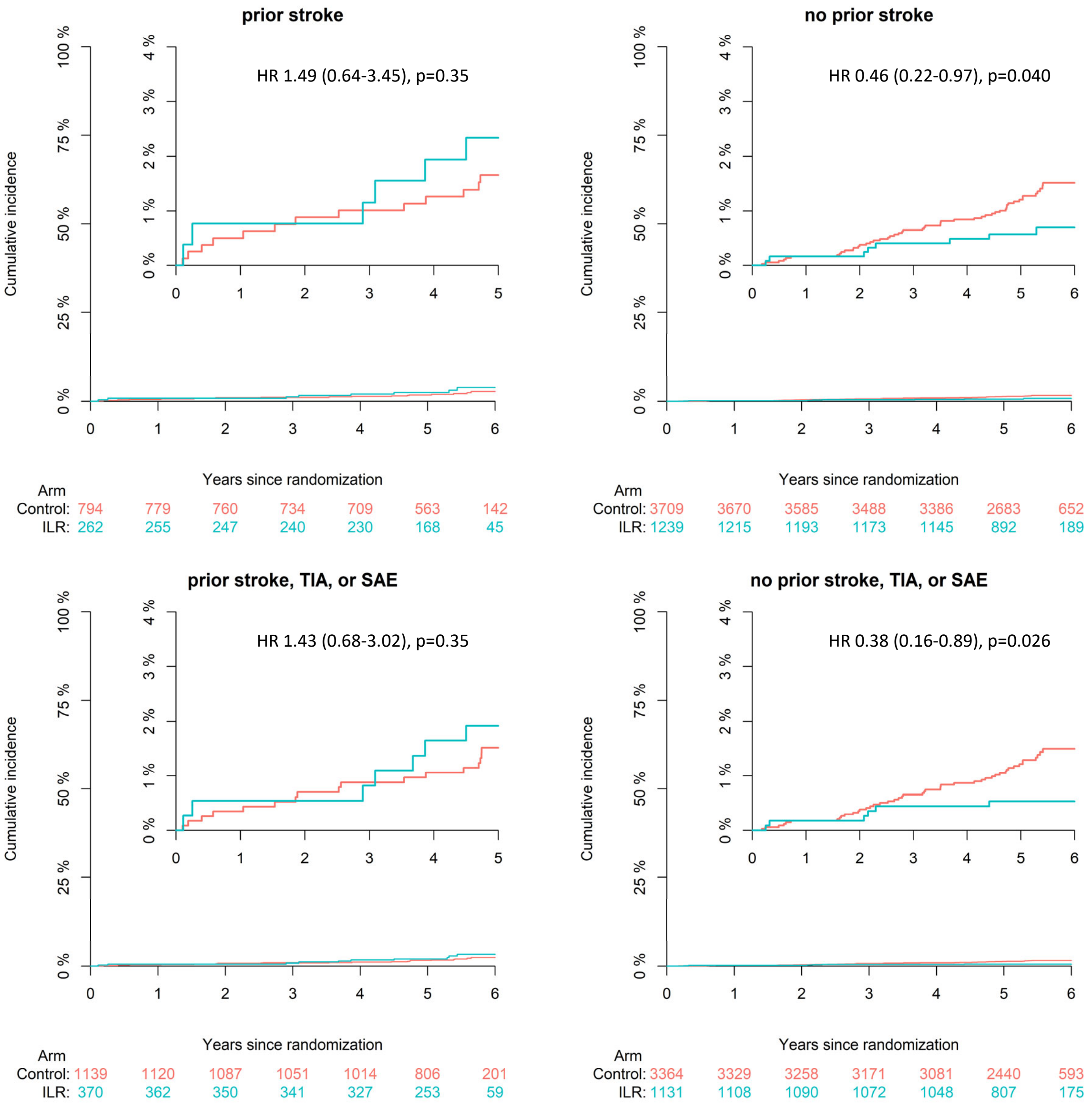

eFigure 11. Cumulative Incidence of Severe Cardioembolic Stroke or ESUS by Stroke History

The figure shows the cumulative incidence curves for disabling or lethal (mRS≥3) cardioembolic stroke or ESUS comparing the Control and ILR group, stratified by prior stroke (**upper panels**) and prior stroke, TIA, or SAE (**lower panels**), respectively. ESUS, embolic stroke of undetermined source; HR, hazard ratio; ILR, implantable loop recorder; mRS, modified Rankin Scale; SAE, systemic arterial embolism; TIA, transient ischemic attack.

© 2022 Diederichsen SZ et al. *JAMA Neurology.*

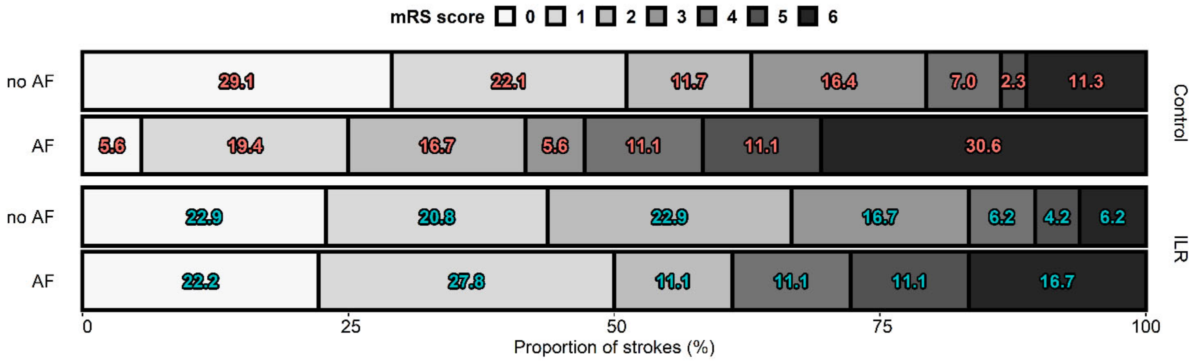

eFigure 12. Stroke Severity Grouped by AF Diagnosis and Randomization Group

The figure shows the distribution of mRS scores following discharge from stroke admission according to diagnosis of AF before or on the day of admission vs no AF in the Control and ILR group, respectively. Wilcoxon rank sum test revealed a significantly higher mRS score among AF-patients compared to patients without AF within the Control group ($p<0.0001$), but not the ILR group ($p=0.63$). AF, atrial fibrillation; ILR, implantable loop recorder; mRS, modified Rankin Scale.

© 2022 Diederichsen SZ et al. *JAMA Neurology.*
