# Supplementary material for: Severity and Etiology of Incident Stroke in Patients Screened for Atrial Fibrillation vs Usual Care and the Impact of Prior Stroke: A Post Hoc Analysis of the LOOP Randomized Clinical Trial
Source: JAMA Neurol. 2022 Aug 29;79(10):997–1004. doi: 10.1001/jamaneurol.2022.3031 (PMC9425290; doi:10.1001/jamaneurol.2022.3031)
Supplement: Supplement 3. — Data Sharing Statement [file jamaneurol-e223031-s003.pdf]

Data Sharing Statement

Diederichsen. Severity and Etiology of Incident Stroke in Patients Screened for Atrial Fibrillation vs Usual Care and the Impact of Prior Stroke. *JAMA Neurol*. Published August 29, 2022. doi:10.1001/jamaneurol.2022.3031

Data
Data available: Yes
Data types: Deidentified participant data
How to access data: The data will be part of a consortium within AFFECT-EU and afscreen.org
When available: beginning date: 01-01-2023

Supporting Documents
Document types: None

Additional Information
Who can access the data: Data will be available to researchers in the consortium, fulfilling legal requirements. Protocol and Informed consent forms can be accessed via the primary publication in Lancet (https://www.thelancet.com/journals/lancet/article/PIIS0140-6736(21)01698-6/ppt)
Types of analyses: Data, Protocol and Informed consent forms
Mechanisms of data availability: Wiithout investigator support
